# Structural and Functional Perturbation of *Giardia lamblia* Triosephosphate Isomerase by Modification of a Non-Catalytic, Non-Conserved Region

**DOI:** 10.1371/journal.pone.0069031

**Published:** 2013-07-22

**Authors:** Gloria Hernández-Alcántara, Alfredo Torres-Larios, Sergio Enríquez-Flores, Itzhel García-Torres, Adriana Castillo-Villanueva, Sara T. Méndez, Ignacio de la Mora-de la Mora, Saúl Gómez-Manzo, Angélica Torres-Arroyo, Gabriel López-Velázquez, Horacio Reyes-Vivas, Jesús Oria-Hernández

**Affiliations:** 1 Laboratorio de Bioquímica-Genética, Instituto Nacional de Pediatría, Secretaría de Salud, Mexico City, Mexico; 2 Instituto de Fisiología Celular, Universidad Nacional Autónoma de México, Mexico City, Mexico; National Research Council of Italy (CNR), Italy

## Abstract

**Background:**

We have previously proposed triosephosphate isomerase of *Giardia lamblia* (GlTIM) as a target for rational drug design against giardiasis, one of the most common parasitic infections in humans. Since the enzyme exists in the parasite and the host, selective inhibition is a major challenge because essential regions that could be considered molecular targets are highly conserved. Previous biochemical evidence showed that chemical modification of the non-conserved non-catalytic cysteine 222 (C222) inactivates specifically GlTIM. The inactivation correlates with the physicochemical properties of the modifying agent: addition of a non-polar, small chemical group at C222 reduces the enzyme activity by one half, whereas negatively charged, large chemical groups cause full inactivation.

**Results:**

In this work we used mutagenesis to extend our understanding of the functional and structural effects triggered by modification of C222. To this end, six GlTIM C222 mutants with side chains having diverse physicochemical characteristics were characterized. We found that the polarity, charge and volume of the side chain in the mutant amino acid differentially alter the activity, the affinity, the stability and the structure of the enzyme. The data show that mutagenesis of C222 mimics the effects of chemical modification. The crystallographic structure of C222D GlTIM shows the disruptive effects of introducing a negative charge at position 222: the mutation perturbs loop 7, a region of the enzyme whose interactions with the catalytic loop 6 are essential for TIM stability, ligand binding and catalysis. The amino acid sequence of TIM in phylogenetic diverse groups indicates that C222 and its surrounding residues are poorly conserved, supporting the proposal that this region is a good target for specific drug design.

**Conclusions:**

The results demonstrate that it is possible to inhibit species-specifically a ubiquitous, structurally highly conserved enzyme by modification of a non-conserved, non-catalytic residue through long-range perturbation of essential regions.

## Introduction

In infectious diseases, the goal of rational drug design is to identify an essential biomolecule for the pathogen that can be used as pharmacological target. This biomolecule can then be challenged with chemical compounds that impair its function and which thereafter are used as molecular scaffolds to develop selective drugs [1]. A first and obvious choice is to target a biomolecule that is exclusively present in the parasite. In principle, this approach reduces the possibility that the drug affects the host; however, it has the drawback that the major essential cellular processes are highly conserved through evolution [2], [3]. On the other hand, if the selected target is an orthologous biomolecule found in both, the parasite and the host, the selectivity of the potential drug is a major concern. Indeed, in the case of orthologous enzymes, where essential structural and functional amino acids are highly conserved, the specific inhibition of the enzyme from the pathogen is a major challenge. In this connection, it is noteworthy that triosephosphate isomerase (TIM) has been extensively proposed as a plausible antiparasitic target. TIM is a ubiquitous glycolytic enzyme whose active site and overall α8/β8 barrel domain structure are highly conserved; even so, structural differences in its interface region have been used to achieve specific inhibition of enzymes in parasites [4]-[7].

Among human parasites, *Giardia lamblia* stands out as one of the most common intestinal pathogens; around 280 million people, especially children, are infected around the world [8], [9]. Although current therapeutic options are diverse and effective [9], [10], it has been documented that they have considerable side effects and that clinical and *in vitro* resistance to the commonly used drugs has appeared [11]-[14], thus, there is a need of new antigiardasic therapies. Since *Giardia* lacks oxidative phosphorylation and depends on glycolysis as its major ATP source [15], it has been proposed that disrupting this pathway could hinder the survival of the parasite; therefore, glycolytic enzymes have been considered potential pharmacological targets [16]-[18]. Along this line, we have previously shown that TIM of *G. lamblia* (GlTIM) may be considered a good potential target for antigiardiasic drug design [18]-[21].

**Figure 1:**
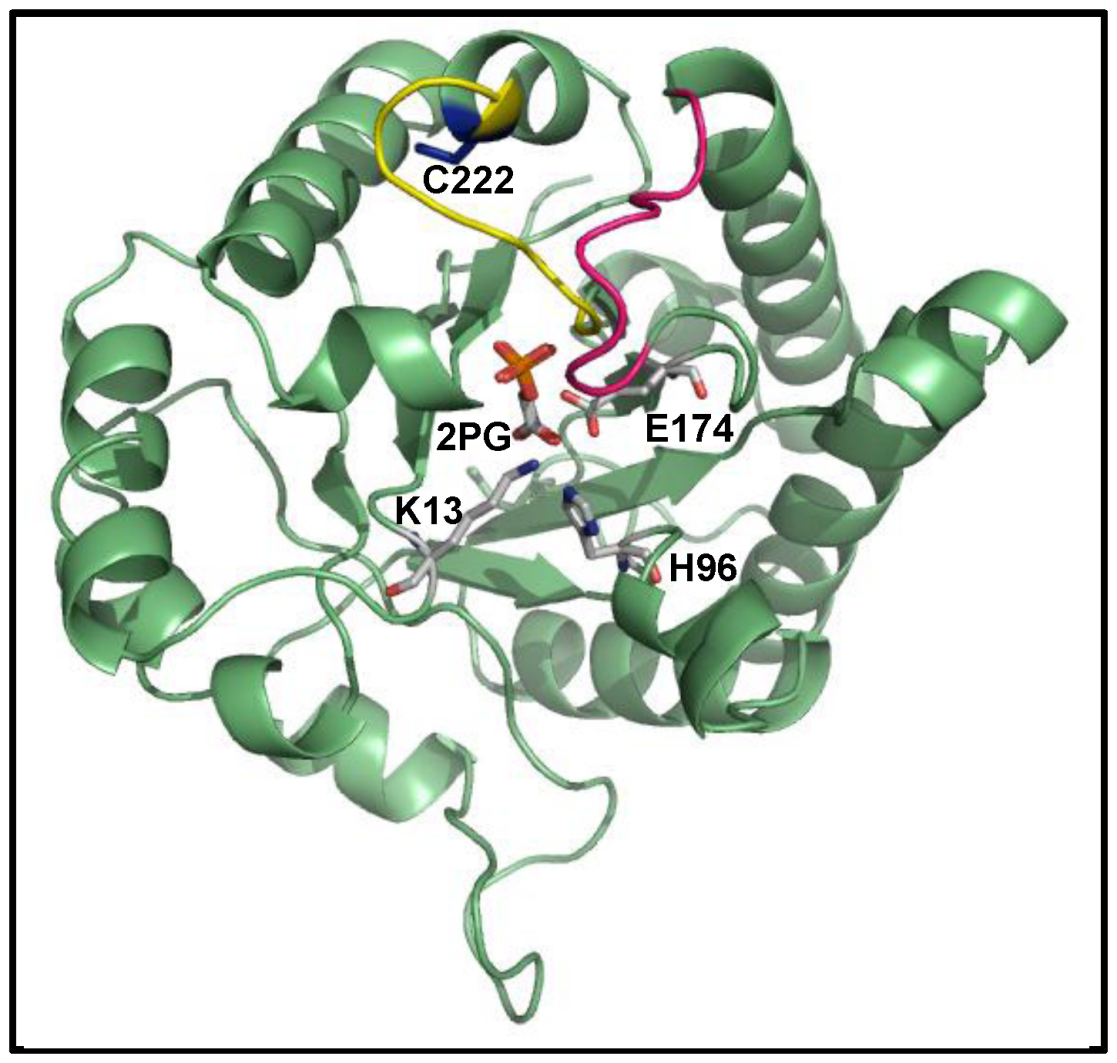
Ribbon representation of WT GlTIM. The catalytic residues (K13, H96, E174) and the substrate analog 2-PG are shown as stick models; loop 6 is depicted in magenta and loop 7 in yellow. C222 is shown as dark blue sticks.

Chemical modification assays of cysteine residues with thiol reactive compounds (cysteine derivatization) were used to identify regions whose modification affects the catalytic activity of GlTIM [20], [21]. Our results indicated that derivatization of C222, a non-interfacial residue located near the surface of the protein and 10-15 Å away from the active site (Fig. 1), specifically inactivates the enzyme. Mutagenesis assays demonstrated that notwithstanding the fact that GlTIM has four other cysteine residues (C14, C127, C202, and C228), only the modification of C222 brings about the inactivation of the enzyme [21]. In addition, the inactivation is species-specific because, even though human TIM (HuTIM) has an equivalent cysteine (C217), its activity is practically unaffected by thiol reagents [20]. Structural analysis of GlTIM and HuTIM showed that there are marked differences in the neighboring regions of C222 of GlTIM and C217 of HuTIM; only one out of seven residues surrounding both cysteines is common [20]. These observations support the proposal that the C222-region can be used as a specific target for rational drug design [20], [21].

**Figure 2:**
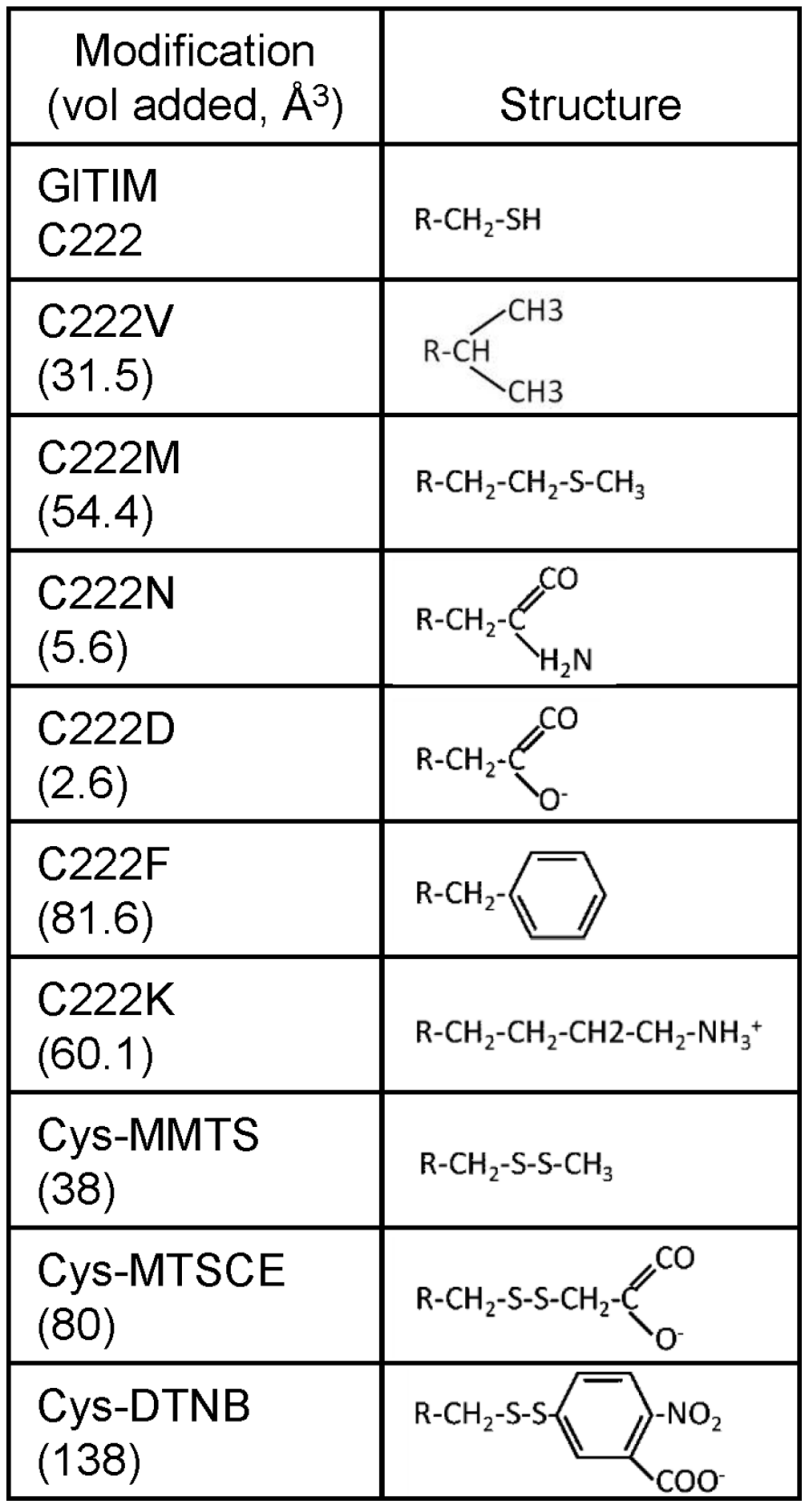
Chemical structure of the modifications of C222 introduced by site directed mutagenesis or chemical modification. Values in parentheses indicate the volume added by the modification (volume of the lateral chain of the amino acid or chemical group introduced minus the volume of the lateral chain of the cysteine residue). For simplicity, only the structure of the lateral chain is shown; R indicates the alpha carbon.

Regarding the inactivation mechanism of GlTIM by chemical modification of C222, it is relevant to point out that derivatization of this residue with the small non-polar thiomethyl group of methylmethane thiosulfonate (MMTS) (Fig. 2), reduced the catalytic activity of GlTIM by half [21]. In contrast, the enzyme activity was completely abolished by the introduction of bulky, negatively charged thiocarboxyethyl or thionitrobenzoate groups added by derivatization with 2-carboxyethyl methanethiosulfonate (MTSCE) or 5,5′-dithio-bis (2-nitrobenzoic acid) (DTNB) (Fig. 2). These observations suggested that inactivation correlated with the physicochemical properties of the derivatizing agent. However, in the data, it was not clear if the steric or electrostatic factors were determinant for the inactivation of GlTIM [21]. Moreover, the crystal structure of GlTIM derivatized with MMTS at positions C14, C222 and C228, did not show alterations that could explain the effect of derivatization on the activity of GlTIM [21].

In this work, we use site directed mutagenesis to extend our understanding of the functional and structural consequences induced by modification of C222. We hypothesized that the effect of the mutation of C222 by amino acids with side chains with different characteristics (Fig. 2) could mimic the effect of the derivatizing agents, and thus reveal how a given molecular group at this position disturbs GlTIM. Our results show that modification of volume, polarity and charge in the side chain of residue 222 differentially affect the catalytic properties, the stability and the structure of GlTIM. Thus, it was possible to decode the contribution of specific physicochemical determinants to the molecular mechanism of GlTIM perturbation by modification of C222.

## Materials and Methods

### General Procedures

Glyceraldehyde 3-phosphate (GAP), buffers, salts and all the other analytical grade reagents were acquired from Sigma–Aldrich; α-glycerol-3-phosphate dehydrogenase (α-GDH) was from Roche, crystallization screen kits were obtained from Hampton Research. Restriction enzymes, DNA polymerase and T4 DNA ligase were purchased from New England BioLabs and Invitrogene. Oligonucleotide synthesis and DNA sequencing was performed at the Unidad de Biología Molecular, Instituto de Fisiología Celular, UNAM. Protein concentration was determined by the bicinchoninic acid method or by absorbance at 280 nm for pure GlTIM (ε_280_ = 26600 M^-1^cm^-1^) [18]. The TIM activity was determined at 25°C by a coupled assay, following the decrease in absorbance at 340 nm [22]. The standard reaction mixture contained 100 mM triethanolamine/10 mM EDTA, pH 7.4 (TE buffer), 1 mM GAP, 0.2 mM NADH, and 0.9 units of α-GDH (20 μg/mL); the reaction was initiated by addition of 5 ng/mL of WT, C222V or C222M GlTIM, 10 ng/mL of C222N, 20 ng/mL of C222D or C222F, and 200 ng/mL of C222K. Kinetic, binding and stability data were analyzed by non-linear regression calculations with Origin software.

### Construction, Expression and Purification of WT and Mutated GlTIM

GlTIM mutants C222V, C222M, C222N, C222D, C222F and C222K, were constructed by site-directed PCR mutagenesis using as template the previously isolated *gltim* gene cloned in the pET3a vector [18]. The mutagenic oligonucleotides were, for C222V Forward (Fw) 5′-GGAAGCAACGTGGAGAAG-3′ and 5′-CTTCTCCACGTTGCTTCC-3′ Reverse (RV); for C222M Fw 5′-GGAAGCAACATGGAGAAGC-3′ and Rv 5′-GCTTCTCCATGTTGCTTCC-3′; for C222N Fw 5′-GGAAGCAACAACGAGAAGC-3′ and Rv 5′-GCTTCTCGTTGTTGCTTCC-3′; for C222D Fw 5′-GGAAGCAACGATGAGAAGC-3′ and Rv 5′-GCTTCTCATCGTTGCTTCC-3′; for C222F Fw 5′-GGAAGCAACTTTGAGAAGCT-3′ and Rv 5′- AGCTTCTCAAAGTTGCTTCC-3′; and for C222K Fw 5′-GGAAGCAACAAAGAGAAGCT-3′ and Rv 5′-AGCTTCTCTTTGTTGCTTCC-3′. In all cases, external T7 promoter and terminator oligonucleotides (Novagen) were used. Mutagenesis was performed using the following PCR conditions: 94°C for 4 min, 25 cycles for 1 min at 94°C, 1 min at 55°C, 1 min at 72°C and 10 min at 72°C. Successful mutagenesis was confirmed by automated DNA sequencing of the complete genes. The PCR products were cloned into the pET-HisTEVP plasmid after digestion with *Nde*I and *Bam*HI; this vector introduces a (His)6-tag and a tobacco etch virus protease recognition sequence at the NH-terminus of the protein [21]. Expression in BL21(DE3)pLys cells and purification of recombinant GlTIM by immobilized metal ion affinity chromatography were performed as recently reported [21]. For all proteins, purity was higher than 95% as determined by SDS-PAGE and MALDI-ToF mass spectrometry at linear mode (data not shown).

### Kinetic Assays

Determination of K_m_ and *V_max_* was performed by fitting initial velocity data at GAP concentrations ranging from 0.3 to 3 mM to the Michaelis-Menten equation (v = Vmax•S/Km+S) by non-linear regression calculations. The *k_cat_* was derived from *V_max_* by considering a molecular mass for the monomer of 27.7 kDa. The K_i_ for 2-phosphoglycolate (2-PG) was calculated by global fit of initial velocity curves (GAP from 0.3 to 3 mM) obtained at different fixed concentrations of the inhibitor. Prior to fitting collectively the data to the simple competitive inhibition equation (v = Vmax•S/Km(1+I/Ki)+S), individual data were plotted as double reciprocal plots to confirm competitive inhibition.

### Structural Spectroscopic Assays

Spectroscopic assays were essentially performed as reported [20], [21]. Circular dichroism assays were performed using a Jasco J-810 spectropolarimeter equipped with a thermostated Peltier-controlled cell holder in a quartz cell with a path length of 0.1 cm. Spectral scans at 25°C were performed from 200 to 260 nm at 1nm intervals with 100 μg/mL of protein, previously dialyzed against 25 mM phosphate pH 7.4. Protein stability was evaluated by the change in the circular dichroism signal at 222 nm in temperature scans from 25 to 70°C with increments of 1°C/min in TED buffer pH 7.4 (100 mM triethanolamine, 10 mM EDTA and 1mM DTT). The fraction of unfolded protein and melting temperature (Tm) values were calculated as previously indicated [20]. Fluorescence assays were conducted at 25°C in TE buffer with 0.4 mg/mL GlTIM in a Perkin-Elmer LS-55 fluorescence spectrometer. Emission fluorescence spectra from 310 to 500 nm were recorded after excitation at 280 nm; bandwidths for excitation and emission were 13.2 and 3.6 nm, respectively. In all spectroscopic assays, the spectra of samples without protein were subtracted from those that contained the enzyme.

### Binding of 2-phosphoglycolate to GlTIM

The change in the intrinsic fluorescence of GlTIM in response to the addition of the transition state analog 2-PG was used to determine the affinity of the enzyme for this ligand, as previously reported [21]. Intrinsic fluorescence spectra were collected before and after each addition of 1 or 2 μL aliquots of a 3 or 30 mM stock solution of 2-PG. The final volume added to the fluorescence cell was always less than 5% of the total volume; there was no effect on the absorbance of the protein solution at 280 nm at the highest 2-PG concentration assayed. In all cases, the spectra of samples without protein were subtracted from those that contained the enzyme. For each mutant, the maximal fluorescence intensity at 332 nm, for every 2-PG concentration, was plotted and fitted to y = (α/2Et)(Et+x+Kd) - √ (Et+x+Kd)^2^– (4xEt), where y = 1 - (Fi/F) (Fi, initial fluorescence; F, fluorescence intensity at each 2-PG concentration); α is the maximal fluorescence change attained at saturating concentration of the ligand, E_t_ represents the concentration of binding sites, K_d_ is the equilibrium dissociation constant and x is the concentration of 2-PG [21], [23].

### Crystallization, Data Collection, Structure Determination and Refinement of WT GlTIM and C222 Mutants

As previously reported, the crystallization assays of GlTIM are conducted with the C202A mutant, which forms stable dimers that allows obtaining crystals suitable for diffraction [19]. The C202A mutation does not have appreciable effects on the catalytic properties or the susceptibility to cysteine derivatizants of GlTIM [19]. The C222 mutants used in crystallization experiments were constructed as double mutants using the GlTIM C202A as scaffold. For simplicity, proteins used in crystallography experiments are designed hereafter as: WT^C^ (C202A), C222N^C^ (C202A/C222N), C222D^C^ (C202A/C222D), C222K^C^ (C202A/C222K) and C222F^C^ (C202A/C222F). Crystallization assays were performed with the sitting drop vapor diffusion method in 96 well plates. For all proteins, one microliter of reservoir solution (Crystal Screen kits, Hampton Research) was mixed with 1 μl of protein solution (WT^C^ 30 mg/mL, C222N^C^ 18 mg/mL, C222D^C^ 20 mg/mL, C222K^C^ 34 mg/mL and C222F^C^ 35 mg/mL) supplemented with 5 mM of 2-PG. Crystals were obtained in the following conditions: WT^C^, F10-Crystal Screen I; C222N^C^, F4-Crystal Screen II; C222D^C^, F12-Crystal Screen II and C222K^C^, H11-Crystal Screen II. The C222F^C^ mutant did not crystallize in any assayed condition. Prior to freezing in liquid nitrogen, crystals were cryoprotected by adding gradually increased concentrations of cryoprotectants as follows: for WT^C^, PEG-3350 up to 35%; for C222N^C^, glycerol up to 20%; for C222D^C^, ethylene glycol up to 15% and for C222K^C^ no cryoprotectant was added. Diffraction data were collected at the Life Sciences Collaborative Access Team (LS-CAT) 21-10-F and 21-ID-F beam lines at the Advanced Photon Source (Argonne National Laboratory), using a CCD detector. Crystals of C222K^C^ did not render interpretable diffraction patterns and were discarded from further analysis. Diffraction data were processed with MOSFLM [24], and reduced with SCALA [25]. Crystal structures were solved by the molecular replacement method with the PHASER software [26], using the coordinates of the previously reported WT^C^ GlTIM [19] (PDB code 2DP3) as the starting model. Refinement was made with the program REFMAC 5 [27], followed by model building with COOT [28]. Five per cent of the data were used to validate the refinement. Water molecules were added to the model near the end of refinement based on difference maps (peak observed above 3σ on a difference map and above 1.5σ on a double difference map) and bond distance criteria (at least one polar contact between 2.6 and 3.5 Å). Model validation was performed with PROCHECK [29]; σA-weighted 2F_0_-2F_c_ and F_0_-2F_c_ simulated annealing omit maps were used to further validate the quality of the model maps. Crystallographic contacts were analyzed with CryCo [30]; figures were prepared with PyMOL (www.pymol.org). Data collection and refinement statistics are given in Table 1. The atomic coordinates and structure factors have been deposited in the Protein Data Bank (PDB) with accession numbers 4BI7 for WT^C^, 4BI6 for C222N^C^ and 4BI5 for C222D^C^.

**Table 1:** Data collection, refinement statistics and quality of the models.

DATA COLLECTION STATISTICS			
PARAMETERS	WT^C^ (C202A)	C222N^C^ (C202A/C222N)	C222D^C^ (C202A/C222D)
Space group	I222	I222	P1
Monomers per asymmetric unit	1	1	20
Unit cell: a,b,c (Å)	55.5,102.0,118.7 90,90,90	55.5,100.4,118.1 90,90,90	105.2,131.5,132.5 115.7,89.8,90.2
Resolution range (Å)	38.6-1.6	38.3-1.5	79.0-2.7
Unique reflections	43,653	58,919	147,201
Average multiplicity	3.7(3.7)	6.8(6.8)	1.6(1.5)
Completeness (%)	99.3(100)	99.8(100)	82.0(84.7)
*I/σ(I)*	5.9 (2.8)	3.7(2.1)	6.2 (2.5)
*Mn(I)/sd*	12.5(4.3)	13.0(4.7)	5.7(1.4)
R_merge_ (%)	6.0(27.0)	9.1(35.7)	6.7(29.1)
R_work_/R_free_ (%)	18.0/19.8	18.9/20.6	23.9/27.2
Water molecules per asymmetric unit	192	220	0
RMSD from ideal: bond lengths (Å)	0.009	0.006	0.008
RMSD from ideal: bond angles (^o^)	1.2	1.0	0.94
Mean overall B value (Å^2^)	23.1	18.7	48.1
Ramachandran plot (%): Allowed/not allowed	97.02/2.98	97.3/2.7	95.77/4.23
PDB code	4BI7	4BI6	4BI5

Values in parentheses are for the last resolution shell.

### Sequence Conservation Analysis

Amino acid sequences from TIM were retrieved from the Reference Sequence collection (RefSeq) at the National Center for Biotechnology Information [31]. In order to cover a broad spectrum of phylogenetic groups, at least a sequence from main taxon orders was selected. The final number of sequences used for alignment was 207. Progressive multiple sequence alignment was calculated with the Clustal_X package [32], using the Gonnet 250 matrix [33]. The complete alignment is included in Alignment S1.

## Results and Discussion

### Purification and Spectroscopic Characterization of Recombinant Proteins

WT GlTIM and the C222 mutants were purified to homogeneity; the (His)6-tag and TEV cleavage systems yielded around 50-60 mg of pure recombinant protein per liter of cell culture. The spectroscopic structural characterization showed that the far-UV circular dichroism spectra of all mutants resemble the spectrum of the WT protein (Fig. S1A). Similar results were obtained in the intrinsic fluorescence studies; the spectra of all the mutants showed only minor differences with respect to the WT GlTIM spectrum (Fig. S1B). All together, the spectroscopic studies indicated that in all the C222 mutants, the global structure of GlTIM is preserved.

### Kinetic Characterization of WT GlTIM and C222 Mutants

We have previously studied the effect of chemical modification of cysteine residues on the activity and structure of GlTIM [20], [21]; it was shown that inactivation of GlTIM by derivatization of C222 with thiol reactive reagents seems to correlate with the physicochemical properties of the modifying agent. In consonance with these observations, we have now found that the kinetics of the six C222 variants of GlTIM was affected to different extents by residues that have different physicochemical properties in their side chains (Table 2). The introduction of valine, a non-polar and slightly larger residue than cysteine (+31.5 Å^3^), marginally affected the kinetic properties of GlTIM. This result is consistent with previous data that showed that the change of C222 by alanine, a non-polar and smaller residue than cysteine, (−19.9 Å^3^), did not affect the activity of GlTIM [19]. In contrast, the change of C222 for methionine, a non-polar larger residue than cysteine (+54.4 Å^3^), induced an eight-fold decrease in the catalytic efficiency of the enzyme (Table 2). The C222M mutation resembles structurally the chemical modification of C222 with MMTS which introduces a non-polar thiomethyl group that increases the volume by 38 Å^3^ (Fig. 2.), and produces a three-fold decrease of the *k_cat_*/K_m_ ratio [20]. Collectively, the results indicate that there is a close correlation between the change of volume of the side chain at position 222 and the inactivation of GlTIM. Changes of 20-30 Å^3^ (alanine and valine for cysteine) are well tolerated, whereas the introduction of a group of 38 Å^3^and 54.4 Å^3^ (MMTS and methionine, respectively) induce important decreases in the catalytic activity of GlTIM.

**Table 2:** Kinetic constants for WT GlTIM and the C222 mutants.

Protein	*V_max_* (μmol min^-1^ mg^-1^)	K_m_ (mM)	*k_cat_* x 10^5^ (min^-1^)	*k_cat_*/K_m_ x 10^5^(min^-1^mM^ -1^)	K_i_ (µM)	K_d_ (µM)
GlTIM	8050±444	0.62±0.22	4.4	7	87±15	26.4±2.6
C222V	9560±467	0.96±0.12	5.2	5.4	98±10	57.4±7.1
C222M	3100±170	1.85±0.46	1.68	0.91	325±50	79.4±8.5
C222N	3653±107	2.79±0.73	1.99	0.71	560±59	113.3±41.7
C222D	3717±421	4.88±0.81	2	0.415	1479±168	n.a
C222F	1665±163	3.9±0.46	0.9	0.23	7280±2010	n.a
C222K	255±33	3.1±0.64	0.13	0.044	n.a	n.a

n.a. not available; experimental data could not be fitted.

Initial velocity rates at GAP concentrations ranging from 0.3 to 3 mM were fitted to the Michaelis-Menten equation to obtain *V_max_* and *K_m_*. The *k_cat_* values were calculated from *V_max_* considering a molecular mass of 27.7 kDa. The K_i_ values for 2-PG were calculated from inhibition assays; initial velocity rates from 0.3 to 3 mM at fixed variable concentrations of 2-PG were globally fitted to a simple competitive inhibition model. K_d_ values for 2-PG were obtained from the change in the intrinsic fluorescence of GlTIM in response to the addition of this ligand as indicated in Material and Methods. For all experiments, the calculated values are the average (± standard error) of two independent experiments.

The relevance of polarity or charge in the inactivation of GlTIM by modification of C222 became apparent when cysteine was replaced by asparagine or aspartic acid (their volumes are similar to that of cysteine, but the former is polar and the other is negatively charged). C222N and C222D respectively exhibited a catalytic efficiency 10-fold and 17-fold lower than that of WT GlTIM (Table 2), indicating that electrostatic factors in the region of C222 are central in the expression of catalytic activity of GlTIM.

We also examined the C222F mutant. The mutant exhibited a 30-fold decrease in the catalytic efficiency of GlTIM. Since the effect of phenylalanine or aspartic acid is less drastic than that induced by the negatively charged aromatic ring of DTNB which induced total inactivation [20], it seems reasonable to assume a synergy of the steric and electrostatic contributions in the inactivation of GlTIM. In this regard, the effect of lysine is illustrative: even though its volume is similar to methionine, the C222K mutant exhibited a higher impairment of catalytic efficiency (159-fold reduction), indicating that the introduction of a positive charge in this region has drastic effects. It is relevant to highlight that we were previously unable to evaluate the effect of cysteine derivatization with positively charged derivatizating agents. Therefore, the C222K mutant provided valuable information on the physicochemical characteristics that an agent should have in order to induce enzyme inactivation. Altogether, the results with the mutant enzymes allowed a dissection of the contributions of steric and electrostatic factors to the inactivation of GlTIM by agents that perturb the region of C222.

### Binding of 2-PG to WT GlTIM and C222 Mutants

As suggested by the kinetic data, a relevant characteristic of the inactivation process caused by modification of C222 is the reduction of the affinity of GlTIM for glyceraldehyde 3-phosphate. To delve into this phenomenon, the binding of the transition state analog 2-PG to WT GlTIM and the C222 mutants was assessed by kinetic and spectroscopic methods (Table 2). The kinetic assays showed that for all enzymes, except C222K where inhibition was hardly observed, 2-PG behaved as a competitive inhibitor (Fig. S2). The K_i_ values for 2-PG for the WT and C222 mutants are shown in Table 2. In consonance with the catalytic data, the affinity of C222 mutants for 2-PG depended on the characteristics of the side chain of the amino acid introduced. The K_i_ of C222V matched the K_i_ for the WT enzyme, whereas the K_i_ values for C222M and C222N were, respectively, 3.7 and 6.4 fold higher; C222F showed a K_i_ that is 17-fold higher than that of WT GlTIM. The most severe changes in the K_i_ values were when negatively and positively charged residues were introduced; for the C222D mutant, an 83-fold increase in the K_i_ was observed. The reduction in affinity was so drastic for C222K that only minimal inhibition was observed even at the highest concentrations of 2-PG that could be assayed, therefore, a K_i_ value could not be estimated. The results indicated that the introduction of a charge in position 222 is a strong factor in the reduction of the affinity of the enzyme for its ligands.

**Figure 3:**
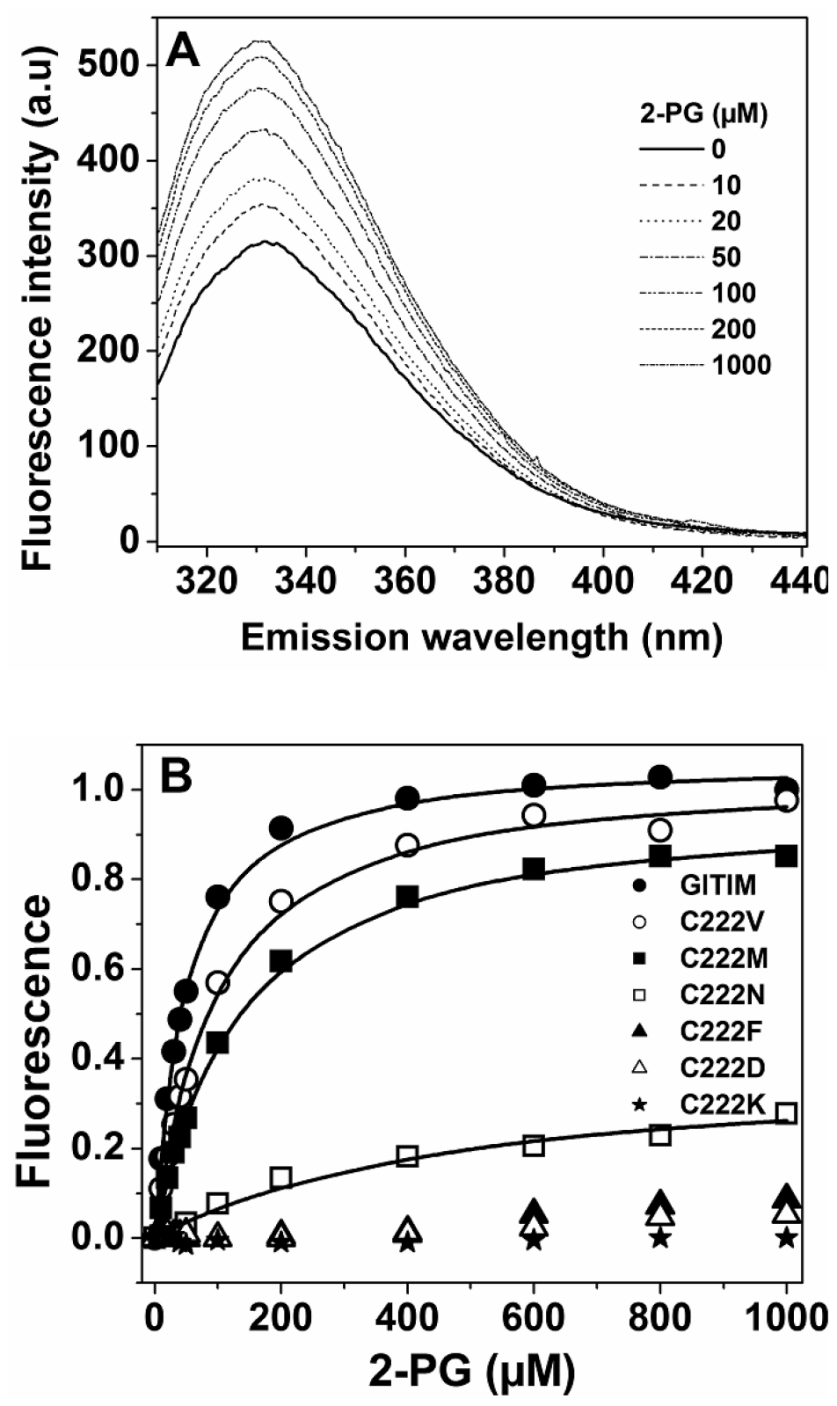
Binding of 2-PG to WT GlTIM and C222 mutants. (A) Fluorescence emission spectra of WT GlTIM in the absence and in the presence of increasing concentrations of 2-PG; for clarity, not all the spectra obtained in the experiment are shown. (B) Plot of maximal fluorescence intensity at 332 nm as a function of the 2-PG concentration for each mutant. For WT GlTIM, C222V, C222M and C222N, solid lines represent the fit of the data to equation y = (α/2Et)(Et+x+Kd) - √ (Et+x+Kd)^2^– (4xEt). For C222F, C222D and C222K the changes of fluorescence were minimal and could not be reasonably fitted.

The reduction of the enzyme affinity, induced by C222 mutations, was confirmed by measuring the changes in the fluorescence of GlTIM induced by 2-PG (Fig. 3 and Fig. S3). In WT GlTIM, the ligand induced an increase of its intrinsic fluorescence intensity (Fig. 3A). The C222 mutants behave similarly to the WT enzyme, except that fluorescence increments varied according to the mutation introduced (Fig. S3). Maximal fluorescence intensities, at each 2-PG concentration for the WT and C222 mutants, are shown in Fig. 3B. From the data on the WT and on the C222V, C222M and C222N mutants, the K_d_ values for 2-PG were calculated [21]. In the C222F, C222D and C222K mutants, the changes in fluorescence induced by 2-PG were minimal and could not be analyzed. The results in Table 2 indicate that the K_d_ values are slightly lower than the K_i_ values obtained in the inhibition assays, which is probably due to the higher sensitivity of the spectroscopic method. However, it is important to note that notwithstanding the differences in the absolute values, the data clearly show the same tendency. The K_i_^MUT^/K_i_^WT^ ratios for C222V, C222M and C222N are 1.13, 3.74 and 6.44, respectively, whereas the corresponding values of K_d_^MUT^/K_d_^WT^ are 2.17, 3.01 and 4.29, indicating that there is agreement between the kinetic and binding data.

Collectively, the kinetic and binding assays confirmed that modification of C222 with polar, charged or bulky lateral chains rendered an incompetent active site with low capacity to bind ligands (substrate or analog) and perform catalysis. Moreover, a comparison of the data in the different C222 mutants with the previous results obtained by chemical modification of GlTIM [21] showed that the action of chemical agents can be mimicked by suitable mutations of C222.

### Stability of WT GlTIM and C222 Mutants

**Figure 4:**
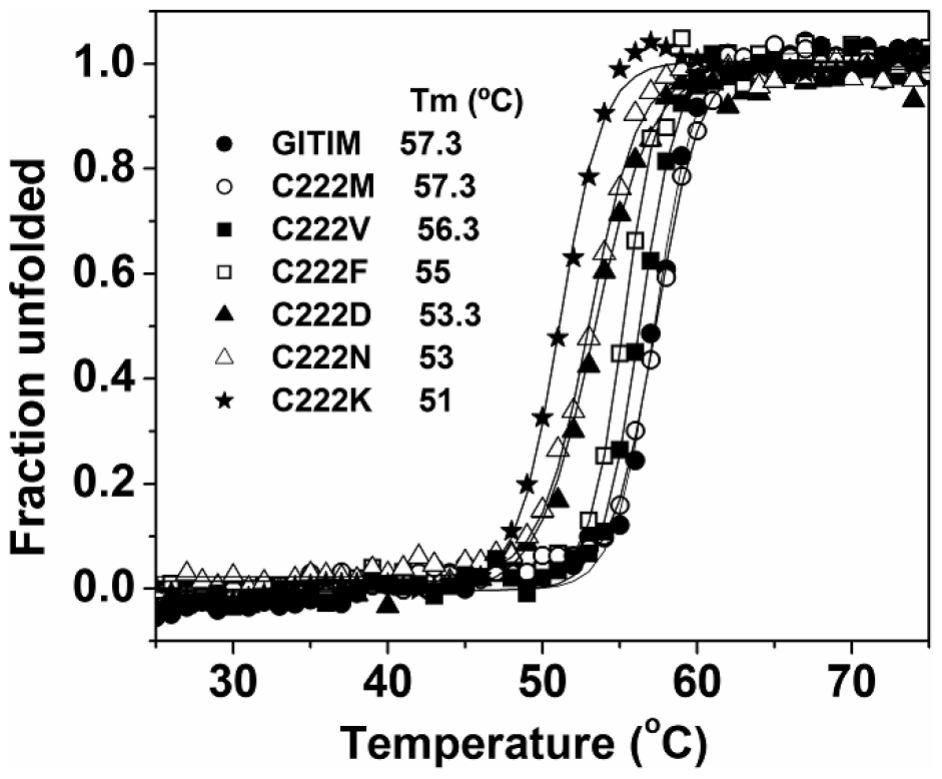
Thermostability of WT GlTIM and C222 mutants. The thermal unfolding of 0.1 mg/ml GlTIM in TED buffer was monitored by recording the change of the circular dichroism signal at 222 nm in a scanning from 25 to 70°C, at a rate of 1°C/min. The fraction of unfolded protein and the Tm values (inset) were calculated as previously described [20]. Experiments were performed by duplicate; in all cases standard errors were less than 5%.

The kinetic and binding data indicated that modification of C222 induces conformational changes that are transmitted as far as 10-15 Å from position 222 to the buried active site (as measured from the sulfur atom of C222 to the 2-PG molecule in the crystal structure of WT GlTIM). To asses if these conformational changes reflect on the protein stability of GlTIM, the thermal stability of the various mutants was determined (Fig. 4). The mutations of C222 for methionine or valine did not affect the stability of GlTIM (ΔTm of 0 and 1°C, respectively). In contrast, mutations that alter the physicochemical character of the side chain brought about decreases in the Tm that ranged from 2.3 to 6.3°C. In this regard, a change in polarity seems to be more important than volume variations; i.e in C222D and C222N, ΔTm values of 4.0 and 4.3°C respectively, were observed, whereas in C222F, the ΔTm was 2.3°C. It is noted that the higher destabilization was induced by the C222K mutation (ΔTm of 6.3°C), which is coincident with the kinetic and binding studies that show that this mutant is the most severely affected protein. Since mutations induce kinetic and stability alterations that follow the same trend, it seems reasonable to assume that the structural modifications that destabilize the protein structure are related to the modification of the catalytic properties of GlTIM (and see below).

### Crystal Structure of WT, C222N, and C222D GlTIM

**Figure 5:**
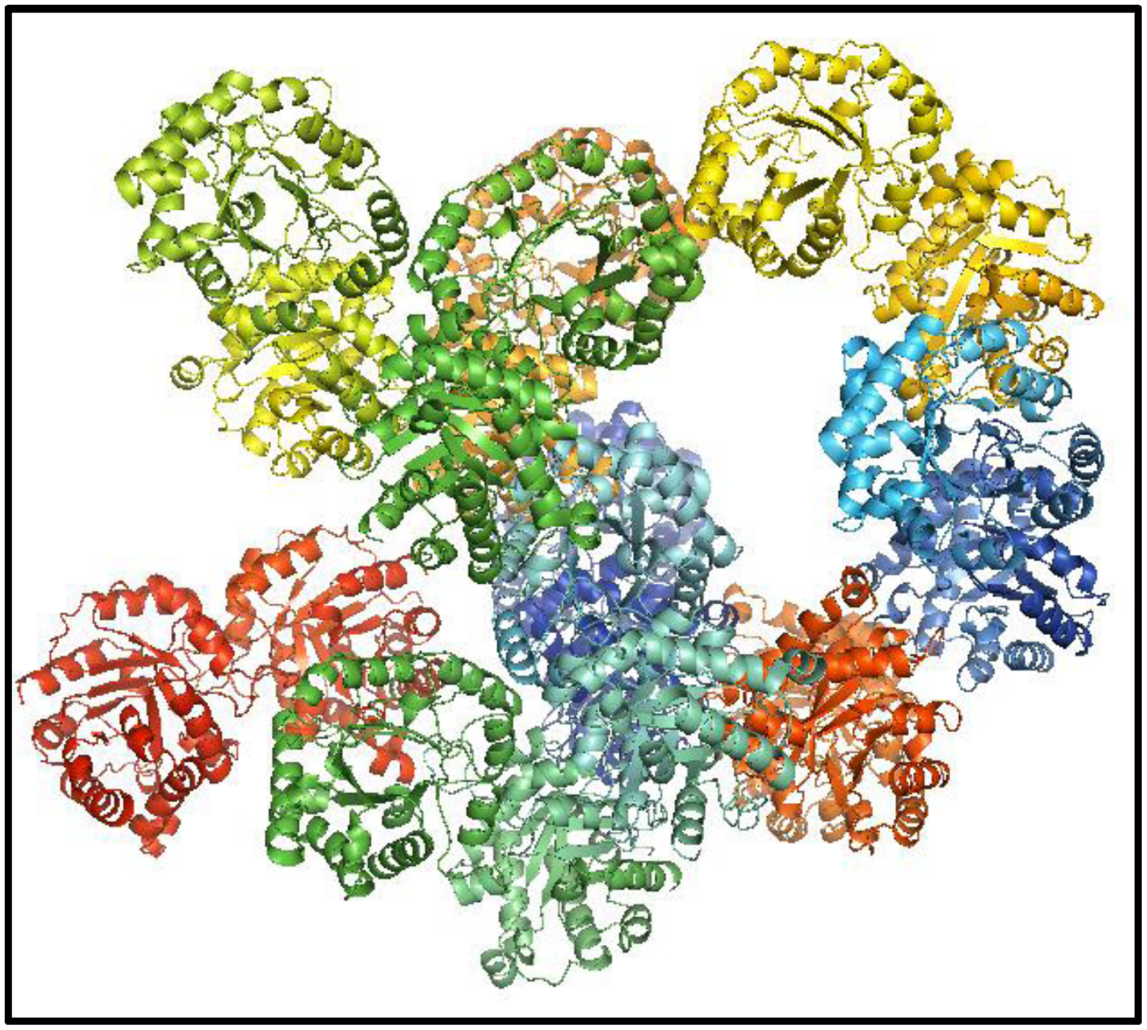
Asymmetric unit of C222D^C^. Each subunit in the asymmetric unit of the GlTIM C222D^C^ crystal is shown in different color.

A central question on the present and previous data [20], [21] is how the modification of C222 is structurally linked to its detrimental effects on GlTIM. To address this question, crystallization trials of WT and C222 mutants were set up. WT^C^, C222N^C^, C222D^C^ and C222K^C^ rendered crystals suitable for diffraction which, with the exception of C222K^C^, generated good quality crystallographic data (Table 1). Similarly to the previously reported structure of WT GlTIM crystalized in absence of ligands (2DP3), WT^C^ and C222N^C^ grew as orthorhombic crystals with space group I222, showing one monomer per asymmetric unit. On the other hand, the C222D^C^ mutant produced triclinic crystals with a P1 symmetry that contains 10 dimers per asymmetric unit (Fig. 5). WT^C^ and C222N^C^ produced high-resolution structures (1.6Å and 1.45Å, respectively), whereas C222D^C^ was solved at a resolution of 2.7Å. In all cases the final maps were of good quality, as indicated by inspection of double difference maps (Fig. S4). Remarkably, initial refinement stages yielded immediate interesting results; even though the three proteins were crystallized in presence of 2-PG 5 mM, only the WT^C^ and C222N^C^ structures displayed electronic densities that corresponded to bound ligand in the catalytic site. The C222D^C^ structure had an empty active site (Fig. S5), which is consistent with the binding data that indicate the low affinity of C222D for 2-PG.

**Figure 6:**
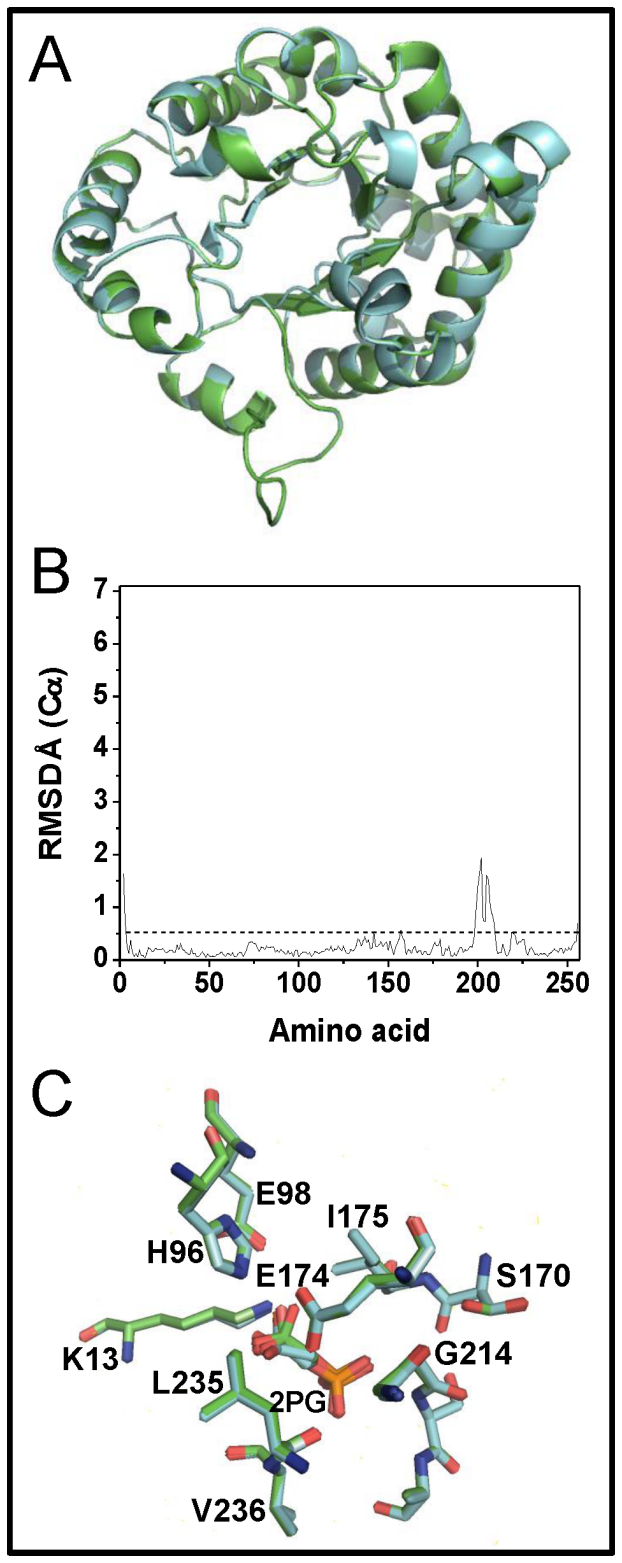
Structural comparison of GlTIM WT^C^ and C222N^C^. (A) Structural alignment of GlTIM WT^C^ (green) and C222N^C^ (cyan); the overall RMSD for these structures is 0.38 Å^2^. (B) Per residue Cα RMSD of GlTIM WT^C^ *versus* C222N^C^; for comparison, the same scale is used in Fig. 7C. (C) Active site comparison of GlTIM WT^C^ (green) and C222N^C^ (cyan).

The structural comparison between WT^C^ and C222N^C^ showed that these structures are almost identical with a Cα RMSD of 0.38 Å^2^ (Fig. 6A). The larger differences were observed in the region of residues 198-207 (Fig. 6B), which corresponds to a small mobile loop in the middle of helix 6. In both structures the architecture of the catalytic site is conserved; the ligand is positioned correctly and the geometry of the active site residues is preserved (Fig. 6C). The result is intriguing because the perturbations in catalysis and stability of C222N do not reflect on discernible structural changes in the crystallographic structure. Nonetheless, the result is consistent with the previously reported crystal structure of GlTIM derivatized with MMTS (PDB code 3PF3), where no conformational changes associated to C222 derivatization were observed [21]. Thus, it is possible that the presence of bound ligand stabilizes the catalytically competent conformation of the active site, masking or preventing the conformational perturbations induced by modification of C222 with MMTS.

**Figure 7:**
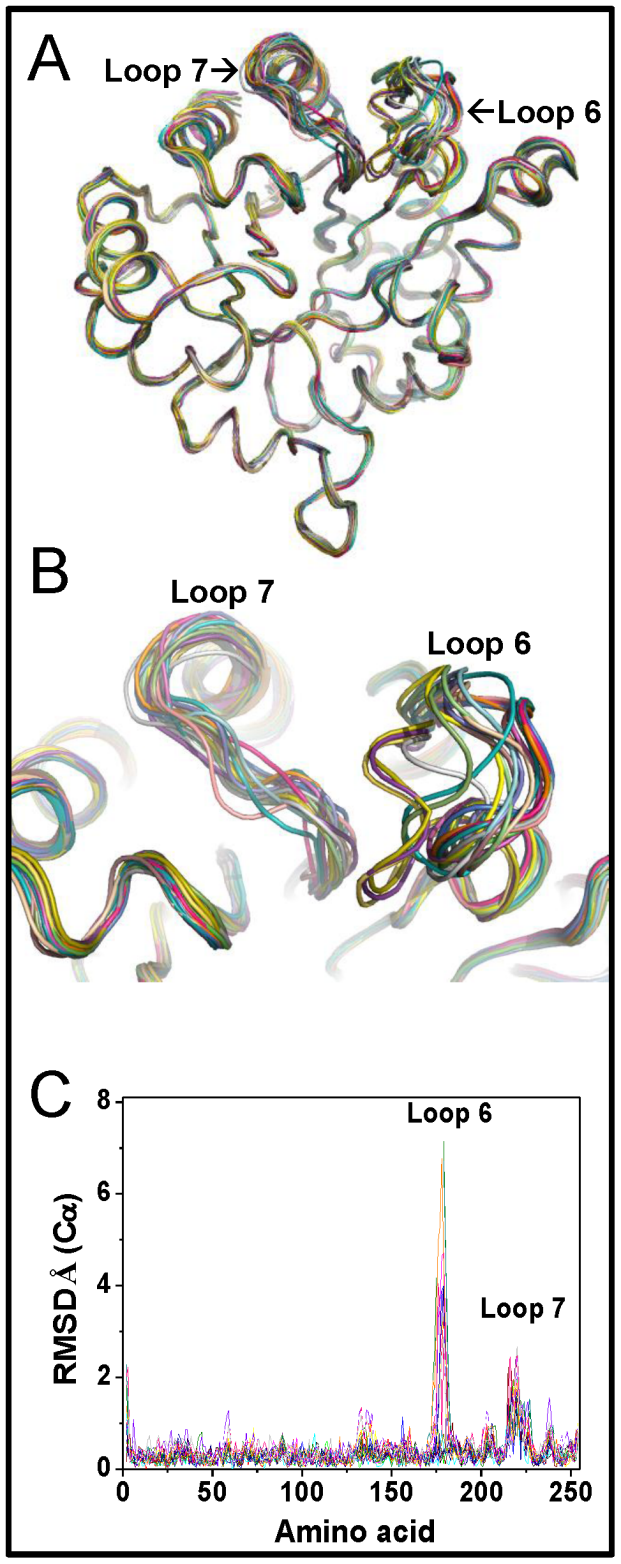
Structural analysis of GlTIM C222D^C^. (A) Structural superposition of the 20 monomers in the crystallographic structure of GlTIM C222D^C^; each chain is shown in different color. (B) Close-up of the superposed loop 6 and 7 regions in GlTIM C222D^C^, which show the major conformational differences between the different chains; the orientation is the same as in panel A. (C) *Per* residue Cα RMSD values of the 20 monomers present in the crystallographic structure of GlTIM C222D^C^; each chain is shown in a different color.

**Figure 8:**
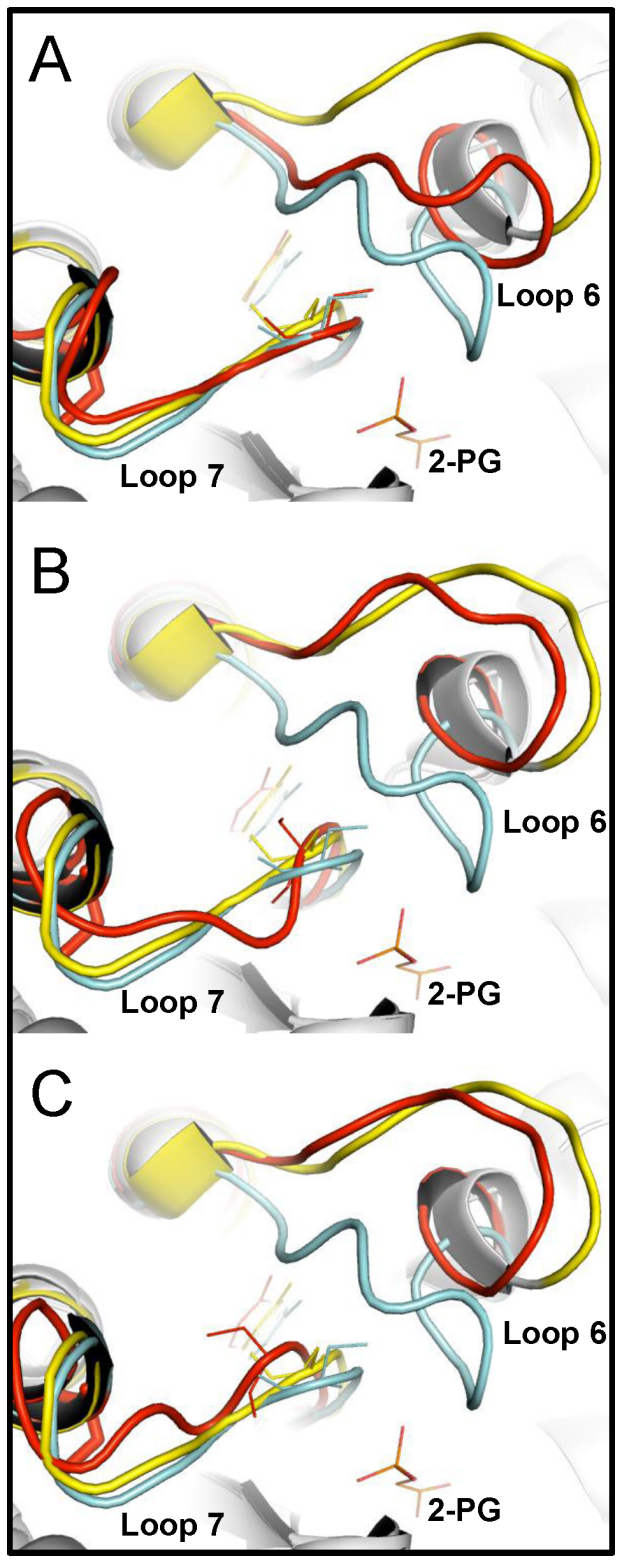
Structural divergence of loops 6 and 7 in the crystal structure of GlTIM C222D^C^. (A) Detailed view of the structural differences occurring in loops 6 and 7 in the chain F of C222D^C^ (red), in comparison with canonical closed (cyan) and open (yellow) states. For the closed state, WT GlTIM crystalized with 2-PG was chosen (4BI7). As GlTIM structure in the open conformation has not been obtained, the closely related structure of *T. vaginalis* TIM (3QST) was used as representative of the TIM-open state. In panels (B) and (C), the same comparison is shown for GlTIM C222D^C^ chains E and H, respectively. The substrate analog 2-PG and lateral chains of the YGGS motif are shown as stick models.

The crystal structure of C222D^C^ provided a clear atomic description of the structural changes induced by modification of C222 that explain the observed detrimental effects on GlTIM (Fig. 7 and Fig. 8). In this structure, it is relevant that the 20 monomers of the asymmetric unit display structural variations in two specific and localized regions of the protein. The structural alignment of all monomers showed that loop 6 (residues 175 to 182) and loop 7 (residues 214 to 221) exhibit a clear heterogeneity of conformational states (Fig. 7A-B), which is evidenced in the per residue α-carbon RMSD values of all chains (Fig. 7C). The conformation variability of loop 6 is not unexpected; in solution, regardless of the occupancy of the active site, this loop switches between open and closed conformations [34]-[36]. These well-defined open and closed conformations have been extensively described in various crystal structures of TIM from different species [37], [38]. In fact, the flexibility of loop 6 is essential for the proper function of TIM [39]. Unexpectedly, however, in some subunits of the C222D^C^ crystal structure, loop 6 is found in an intermediate, non-canonical open or close conformation (Fig. 8). In regard to loop 7 it is important to highlight that even though slight conformational changes in this loop had been previously noted in response to ligand binding [40], the relatively large conformational differences of loop 7 in the C222D^C^ structure has not been previously observed; its main chain atoms move as far as 2.7 Å from their original position (Fig. 7 and 8). It is important to emphasize that neither loops 6 or 7 are implicated in crystallographic contacts in the C222D^C^ structure, excluding the possibility that the observed loop perturbations are due to crystal packing. The result indicates that modification of C222 alters two fundamental loops whose relevance for the proper structure and for the function of TIM has been extensively established.

Loop 7 contains the 213-YGGS-216 motif (GlTIM numbering). In TIM from *Eukaryota* and *Bacteria*, this sequence is highly conserved, whereas in *Archaea* the respective sequence is 213-xGAG-216 (x is mainly C, T, or A) [40]. The importance of the YGGS motif for the function and structure of TIM has been demonstrated [40]-[43]. Y213 and S216 of the YGGS motif interacts via hydrogen bonding with main chain atoms of loop 6 stabilizing the closed conformation of TIM [40], [42]. In the closed conformation, the peptide bond between G214 and G215 in loop 7 rotates by 90°, whereas the peptide bond of G215 and S216 flips the phi/psi angles from (–80°, 120°) in the open form, to (65°, 30°) in the closed form. The former motion places the catalytic E170 into the catalytically competent conformation, whereas the latter motion allows to S216 to interact with the phosphate group of the substrate [40], [44]-[46]. Site directed mutagenesis of the YGGS motif has illustrated the consequences of loop 7 alteration. In yeast TIM [42], the change of tyrosine by phenylalanine in the YGGS motif induced a 2400-fold decrease in the catalytic efficiency of the enzyme, which was accompanied by a 200-fold increase in the K_d_ of the intermediate analogue phosphoglycolohydroxamate (PGH). When serine was replaced by alanine, the catalytic efficiency of the enzyme dropped 30-fold whereas the affinity by PGH increased 5-fold [42]. In chicken TIM, the mutation of the complete YGGS motif for the corresponding *Archaea* motif TGAG decreased the catalytic efficiency 240-fold and reduced the stability of the mutant by 11 K [41]. TROSY-Hahn-Echo and TROSY-selected R1 ρ experiments indicated that mutation of loop 7 nearly doubled the chemical exchange rate for active site loop motion, and reduced the coordinated motion of loop 6 relative to the WT enzyme [41]. The results suggest that in addition to the maintenance of the proper chemical context in the active site, loop 7 also plays an important role in modulating the concerted dynamics of loop 6 hinges, keeping a proper rhythm for the chemical events that take place at the active site of TIM with maximum efficiency [41].

C222 is at the end of loop 7, with its lateral chain positioned in a hydrophobic pocket formed mainly by carbon atoms of residues N218, G219, E223, F234, M248, and I251 (Fig. S6A). Therefore, it is reasonable to assume that the introduction of a negatively charged side chain in the low dielectric environment of this hydrophobic pocket is a thermodynamically unfavorable process that destabilizes the entire loop 7, and alters the structure of the critical YGGS motif. In the light of the critical interactions between the residues of loop 7 and loop 6, it is possible to discern how the modifications of C222 bring about detrimental functional and structural effects on GlTIM. We propose that the well-defined cavity around C222 (Fig. S6B) can be exploited with pharmacological purposes; in fact, the experimental evidence demonstrate that this pocket is large enough to accommodate a molecule with up to 138 Å^3^ as the thionitrobenzoate group added by derivatization with DTNB, inactivating GlTIM.

### Conservation Sequence Analysis

Based on the structural dissimilarity of the C222 region of the *Giardia* and human enzymes, we decided to evaluate the conservation pattern of the C222 region in 207 TIM sequences that span a wide range of taxon orders. The analysis of the multiple sequence alignment indicated that C222 and the six surrounding amino acids are poorly conserved. Each of the seven residues that conforms this region has 7 to 16 natural substitutions in the 207 sequences studied (Table 3); the substitutions include hydrophobic, polar and charged residues (except for position 234, where charged amino acids are not found). Position 222, for example, has preferentially aliphatic amino acids, but aromatic, polar and even negative residues are found at this position. This indicates that there is a complex correlation between amino acid 222 and its surroundings. Along this line, it is interesting to ask if perturbation of the equivalent C222 in a TIM from another species brings about the negative effects that we have observed in GlTIM. In this connection, it is noteworthy and surprising that the C217D mutation in HuTIM (equivalent to the C222D GlTIM mutant) has no appreciable effects on the catalytic properties of the human enzyme (data not shown). These findings support the notion that the C222 region is a species-specific target to inactivate GlTIM.

**Table 3:** Conservation sequence analysis of the C222 region.

GlTIM residue	Naturally occurring substitutions
N218	TKSDQEV
G219	PEAVIDHSY
C222	IAVFTSD
E223	TKSDQARKSVLPGNH
F234	ALVGTCPI
M248	ILVFASK
I251	SYKQFNARVTGMLCED

Naturally occurring substitutions for each amino acid in the C222 region.

The complexity of the C222 region suggests an additional potential benefit as pharmacological target. In regard to drug resistance, it would seem that appearance of resistance will probably need several simultaneous mutations which would certainly decrease the possibility of emergence of drug resistance. In this respect, our data on enzyme inactivation by chemical or mutagenesis modification of C217 in human TIM, along with the structural dissimilarity of the C222 surrounding region of the human and parasite enzymes, suggest that indeed several amino acid changes will be required to decrease the susceptibility of GlTIM to modification of the C222 region. Altogether, the results reinforce the proposal of the C222 pocket of GlTIM as a promising target for drug design.

### Concluding Remarks

Through site directed mutagenesis we assessed the impact of the physicochemical alteration of residue C222 on the catalytic properties, thermostability and structure of GlTIM. The overall data indicate that the introduction of a small polar or charged chemical group in the hydrophobic environment of C222 destabilizes and decreases the affinity and activity of GlTIM. The detrimental effects produced by modification of C222 involve the perturbation of loop 7, a fundamental region in TIM whose interactions with loop 6 are essential for stability, ligand binding and catalysis. We propose that this information can be translated to the design of target-specific molecules that contain the desired physicochemical characteristics.

## Supporting Information

Figure S1 Spectroscopic characterization of WT GlTIM and C222 mutants. (A) Far-UV circular dichroism spectra of WT GlTIM and the C222 mutants. For each protein, the spectral scan of 0.1 mg/ml GlTIM (previously dialyzed against 25 mM phosphate pH 7.4), was performed from 200 to 260 nm at 1 nm intervals. (B) Emission fluorescence spectra of WT GlTIM and C222 mutants. The intrinsic fluorescence spectra of 0.4 mg/ml WT GlTIM and mutants in TE buffer were recorded from 310 to 500 nm after excitation at 280 nm; excitation and emission slits were 13.2 and 3.6 nm, respectively. For all assays, the spectra of the blanks were subtracted from each sample. Each spectrum is the average of three replicated scans.
(TIF)

Figure S2 Inhibition assays of WT GlTIM and the C222 mutants with 2-PG. Initial velocity data at GAP concentrations ranging from 0.3 to 3 mM in the presence of fixed variable concentrations of 2-PG were plotted as double reciprocal plots to confirm competitive inhibition; the Ki values were calculated by global fit of the original data to a simple competitive inhibition model by nonlinear regression calculations.
(TIF)

Figure S3 Fluorescence emission spectra of the C222 mutants in response to 2-PG. The spectra of mutants (0.4 mg/ml) were recorded in TE buffer from 310 to 500 nm, with an excitation wavelength of 280 nm, in the absence and in the presence of increasing concentrations of 2-PG. The experimental conditions were the same as in Figure 3A. For clarity, not all the spectra obtained in each experiment are shown.
(TIF)

Figure S4 Electron density maps around residue 222 in WT GlTIM and two mutants. Double difference (2Fo-Fc) electron density maps contoured at 1.5σ around C222 in GlTIM WT^C^ (A), Asn222 in C222N^C^ (B), and Asp222 in C222D^C^ (C).
(TIF)

Figure S5 Electron density maps of the active site in WT GlTIM and two mutants. Double difference (2Fo-Fc) electron density maps contoured at 1.5σ around the active site of GlTIM WT^C^ (A), C222N^C^ (B), and C222D^C^ (C).
(TIF)

Figure S6 Neighboring region around Cys222. (A) The distances between the sulfur atom of C222 and atoms of adjacent residues (cutoff 5Å) were obtained from the WT^C^ GlTIM structure (PDB code 4BI7); close atoms are shown in yellow (sulfur), gray (carbon), red (oxygen) and blue (nitrogen). (B) Stereo view of the surrounding cavity around C222. The cavity, as calculated by the CASTp server [47], is depicted in orange; C222 is shown in stick model.
(TIFF)

Alignment S1 Aminoacid sequence alignment of triosephosphate isomerase. Progressive multiple sequence alignment of 207 TIM sequences from main taxon orders was performed with Clustal_X as indicated in the Material and Methods section.
(PDF)
