# Supplementary figures and images for: Structural and Functional Perturbation of Giardia lamblia Triosephosphate Isomerase by Modification of a Non-Catalytic, Non-Conserved Region
Source: PLoS One. 2013 Jul 22;8(7):e69031. doi: 10.1371/journal.pone.0069031 (PMC3718800; doi:10.1371/journal.pone.0069031)

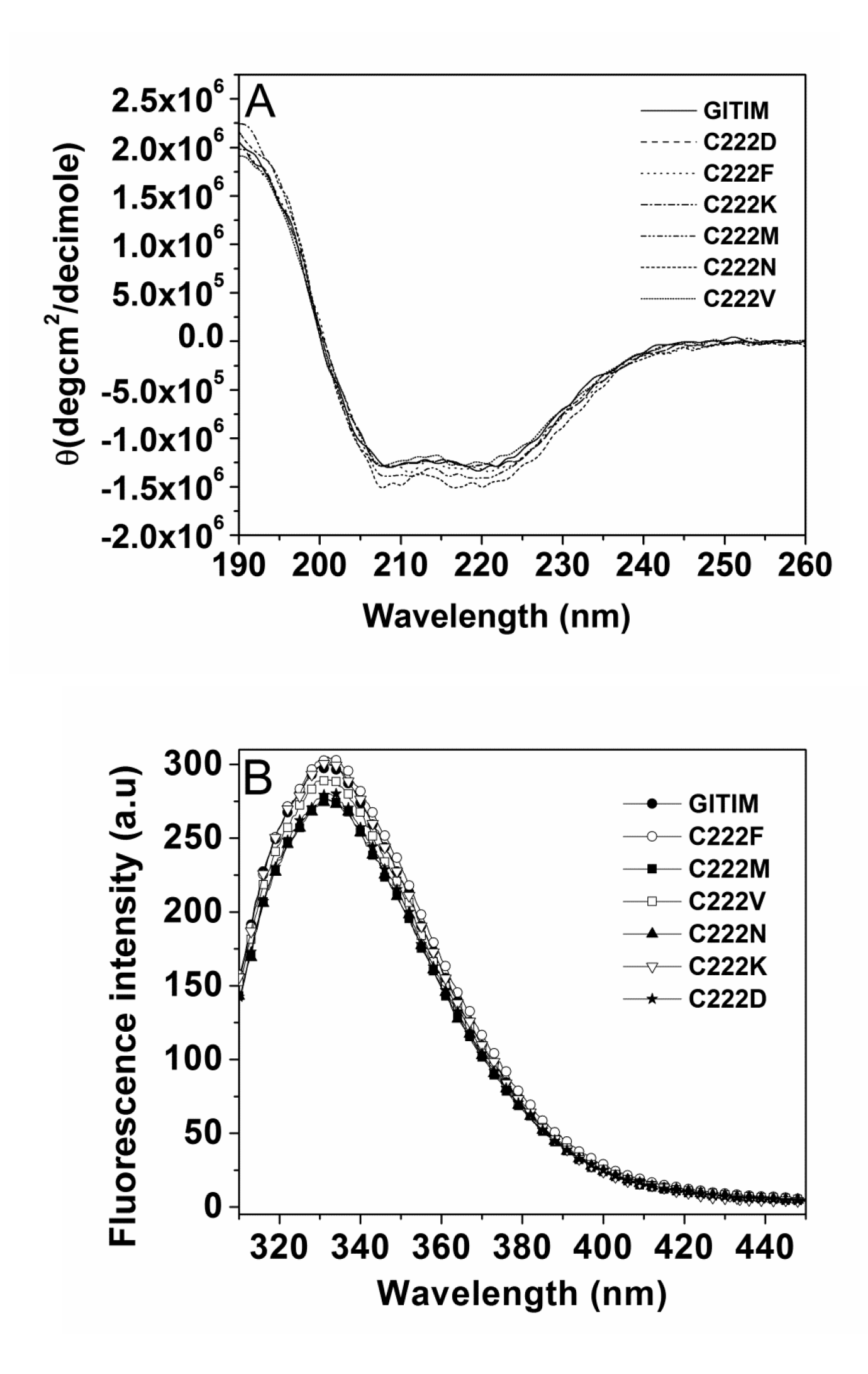

Supplement: Figure S1 — Spectroscopic characterization of WT GlTIM and C222 mutants. (A) Far-UV circular dichroism spectra of WT GlTIM and the C222 mutants. For each protein, the spectral scan of 0.1 mg/ml GlTIM (previously dialyzed against 25 mM phosphate pH 7.4), was performed from 200 to 260 nm at 1 nm intervals. (B) Emission fluorescence spectra of WT GlTIM and C222 mutants. The intrinsic fluorescence spectra of 0.4 mg/ml WT GlTIM and mutants in TE buffer were recorded from 310 to 500 nm after excitation at 280 nm; excitation and emission slits were 13.2 and 3.6 nm, respectively. For all assays, the spectra of the blanks were subtracted from each sample. Each spectrum is the average of three replicated scans. (TIF) [file pone.0069031.s001.tif]

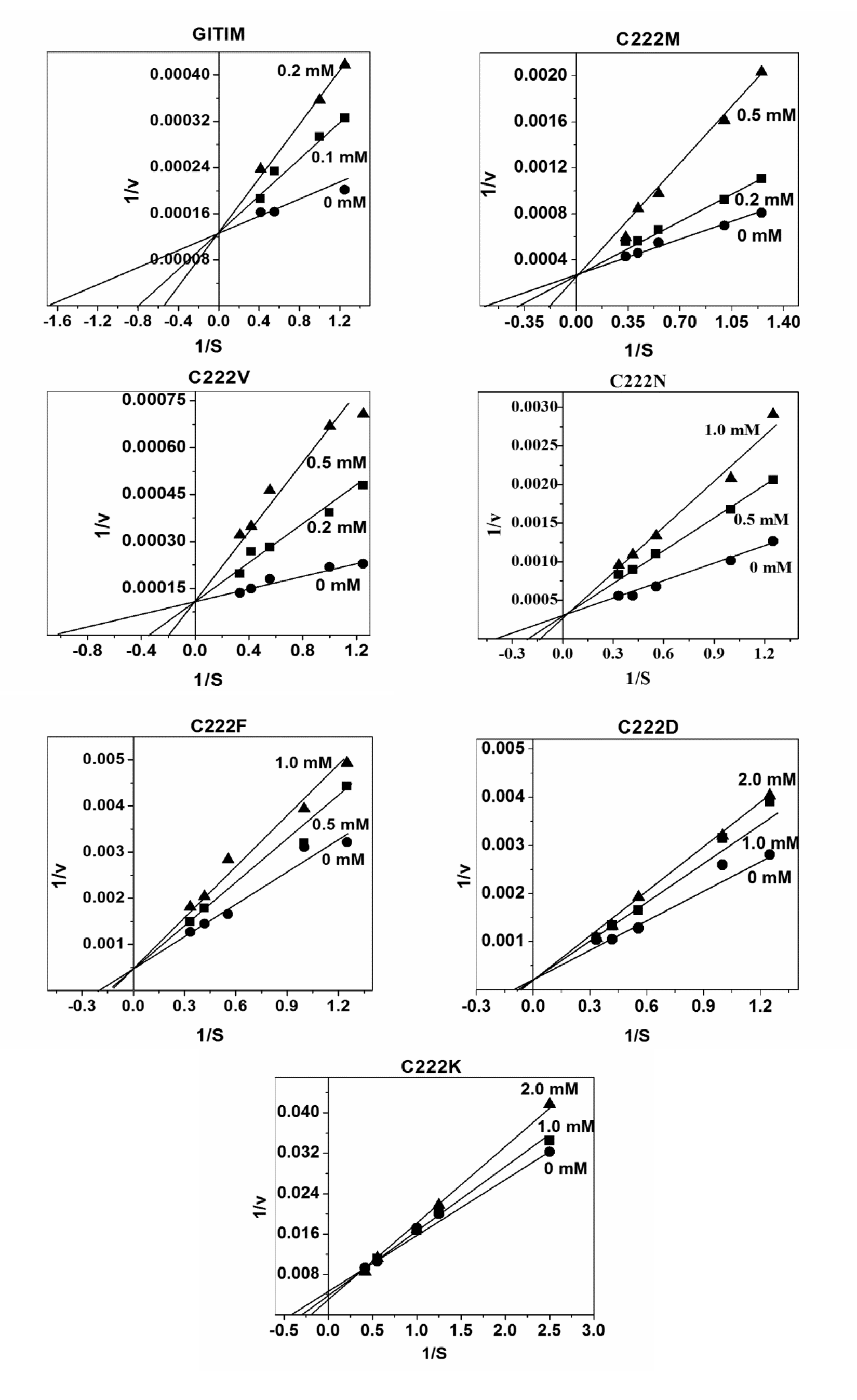

Supplement: Figure S2 — Inhibition assays of WT GlTIM and the C222 mutants with 2-PG. Initial velocity data at GAP concentrations ranging from 0.3 to 3 mM in the presence of fixed variable concentrations of 2-PG were plotted as double reciprocal plots to confirm competitive inhibition; the Ki values were calculated by global fit of the original data to a simple competitive inhibition model by nonlinear regression calculations. (TIF) [file pone.0069031.s002.tif]

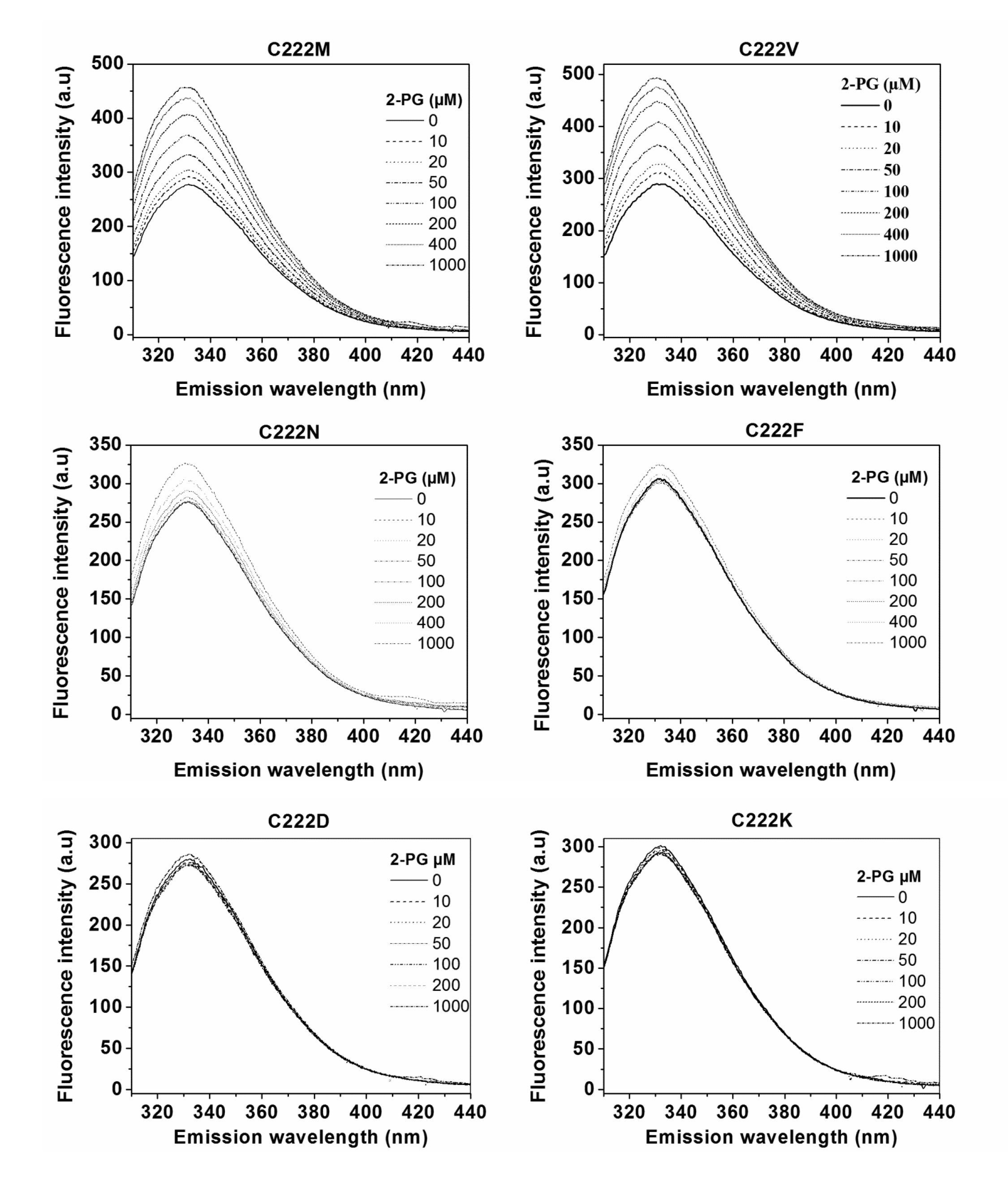

Supplement: Figure S3 — Fluorescence emission spectra of the C222 mutants in response to 2-PG. The spectra of mutants (0.4 mg/ml) were recorded in TE buffer from 310 to 500 nm, with an excitation wavelength of 280 nm, in the absence and in the presence of increasing concentrations of 2-PG. The experimental conditions were the same as in Figure 3A. For clarity, not all the spectra obtained in each experiment are shown. (TIF) [file pone.0069031.s003.tif]

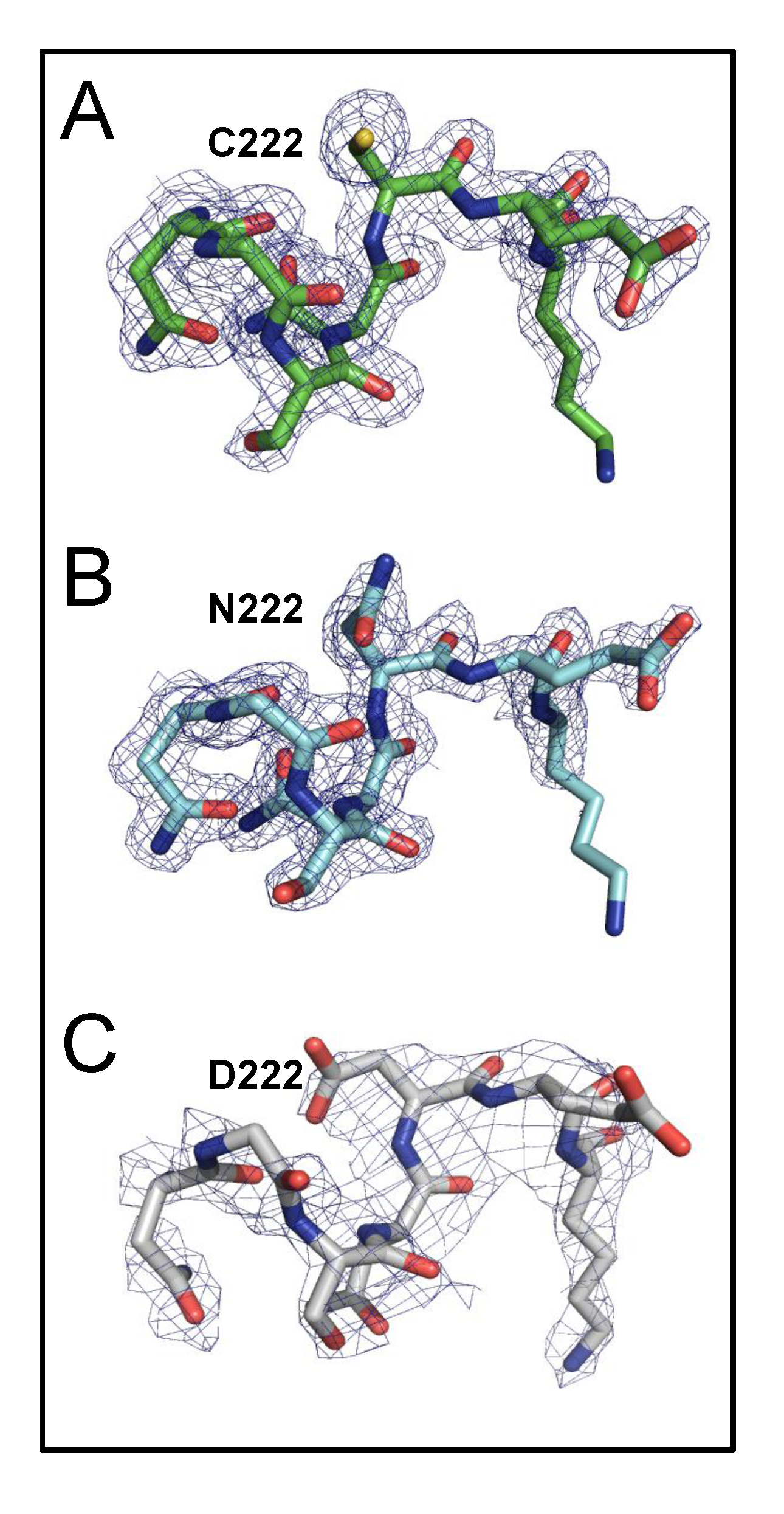

Supplement: Figure S4 — Electron density maps around residue 222 in WT GlTIM and two mutants. Double difference (2Fo-Fc) electron density maps contoured at 1.5σ around C222 in GlTIM WTC (A), Asn222 in C222NC (B), and Asp222 in C222DC (C). (TIF) [file pone.0069031.s004.tif]

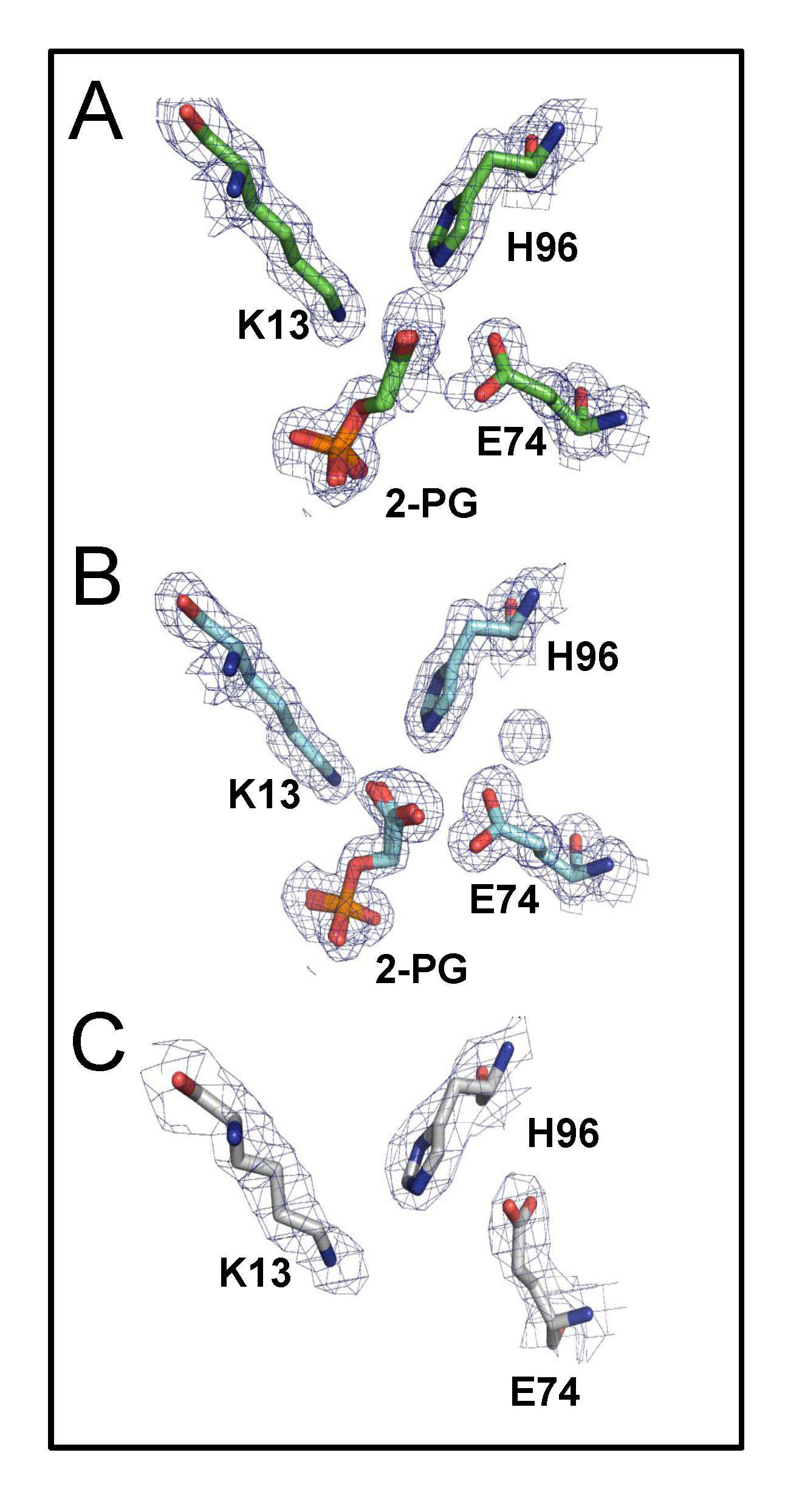

Supplement: Figure S5 — Electron density maps of the active site in WT GlTIM and two mutants. Double difference (2Fo-Fc) electron density maps contoured at 1.5σ around the active site of GlTIM WTC (A), C222NC (B), and C222DC (C). (TIF) [file pone.0069031.s005.tif]

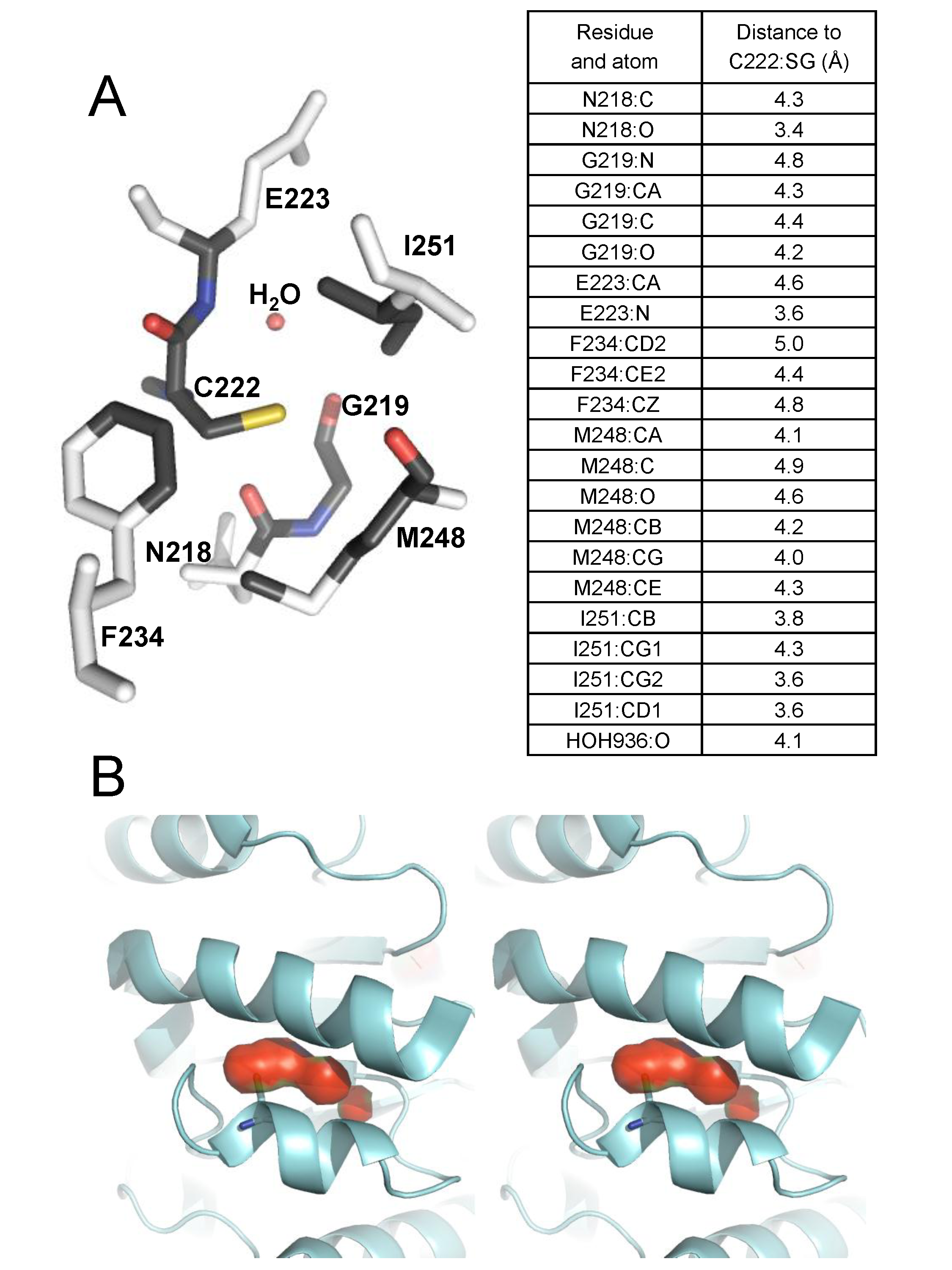

Supplement: Figure S6 — Neighboring region around Cys222. (A) The distances between the sulfur atom of C222 and atoms of adjacent residues (cutoff 5Å) were obtained from the WTC GlTIM structure (PDB code 4BI7); close atoms are shown in yellow (sulfur), gray (carbon), red (oxygen) and blue (nitrogen). (B) Stereo view of the surrounding cavity around C222. The cavity, as calculated by the CASTp server [47], is depicted in orange; C222 is shown in stick model. (TIFF) [file pone.0069031.s006.tiff]
