# Supplementary material for: Structural and Functional Perturbation of Giardia lamblia Triosephosphate Isomerase by Modification of a Non-Catalytic, Non-Conserved Region
Source: PLoS One. 2013 Jul 22;8(7):e69031. doi: 10.1371/journal.pone.0069031 (PMC3718800; doi:10.1371/journal.pone.0069031)
Supplement: Alignment S1 — Aminoacid sequence alignment of triosephosphate isomerase. Progressive multiple sequence alignment of 207 TIM sequences from main taxon orders was performed with Clustal_X as indicated in the Material and Methods section. (PDF) [file pone.0069031.s007.pdf]

PROGRESSIVE MULTIPLE SEQUENCE ALIGNMENT OF TIM FROM MAIN TAXON ORDERS

```
                                           10        20        30        40        50        60        70        80        90       100
                                   ....|....|....|....|....|....|....|....|....|....|....|....|....|....|....|....|....|....|....|....|
Dictyoglomus_thermophilum          -----------------------------MRRKIIAANWKMYKTCAE----TESFIKEFIELSKG-Y--EEKEIVICPPFTSLYVASKLLK-DT---A-I
Aquifex_aeolicus                   -----------------------------MRR-LIAANWKMNKTVKE----TEEYINTFLKFVEH-P--ESREILICPPFTSLYVAGKMLQ-GT---G-V
Thermodesulfovibrio_yellowston     -----------------------------MPKKFIVANWKMHKTVRE----ALAFLDEFIPITKG-L--NGREIGIAPTFICIESVGKVLI-NT---S-I
Dehalogenimonas_lykanthroporep     ---------------------------MSERPTVIAGNWKMHTTVDE----AVELVGEMSSALAE-F--ENVEVIICPPFIALCKVKELLG-QA---NRI
Dehalococcoides_sp                 -----------------------------MRQIIIAGNWKMNTTLSE----ACTLVQSMKCELER-I--EGIEKIICPPFISLYPIKTILE-NS---S-I
Nostoc_punctiforme                 -----------------------------MRKIVIAGNWKMFKTQAE----TQEFLQGFLPHLEE-TP-QGREVILCPPFTDLSVLSKTLH-GS---L-I
Anabaena_variabilis                -----------------------------MRKIVIAGNWKMFKTQAE----SQEFLKEFLPALEE-TP-QEREVLLCVPFTDLAILSQSLH-GS---L-V
Trichodesmium_erythraeum           -----------------------------MRKTIIAGNWKMYKTQLE----AKEFLEGLMSQLTK-TP-EEREVVLCTPFTALDFMSKILH-GS---L-I
Crocosphaera_watsonii              ---------------------------MAVGKIIIAGNWKMHKTQAE----ALEFLTVLKSKVEE-TN-ETREIVLCVPFTALSILSKSLH-GG---K-V
Synechocystis_sp                   -----------------------------MRKIIIAGNWKMHKTQAE----AQAFLQGFKPLIED-AA-ESREVVLCVPFTDLSGMSQQLH-GG---R-V
Synechococcus_sp                   -----------------------------MRKVVIAGNWKMHKTQAD----ASAFVQQLMPQLES-VP-EEREIVLCAPFTTLGLLVDKLQ-SS---R-V
Cyanobacterium_UCYN                -----------------------------MRKKIIAGNWKMHKTQAE----ALEFLSVLKLNLAN-TT-EKADIVLCVPFTLLSLLSKSLH-GG---N-I
Prochlorococcus_marinus            -----------------------------MSKTVIAGNWKMHMTCAQ----ARDFISAYLPLIKN-VP-KGRELVLAPPFTAISTLSEILK-GS---N-V
Chlamydophila_pneumoniae           ----------------------------MTRQSYVLGNWKMHKTIQE----AKEYVQTLASLLQG-EP-LSCTIGIASPFTSLRAIHEMINTTG---AFL
Desulfovibrio_vulgaris             ------------------------------MKKLIAANWKMYKTIDE----ARATGRELVSAVAGSLP-ADREVLVCPPFTALHALHDTFKDVE---GFA
Mesorhizobium_opportunistum        ------------------------------MVYWVGTSWKMNKTLAE----AMDFAAILAGFVPG-FD-DRIQPFVIPPFTAVRQVKQALSSTR---VKV
Geobacter_metallireducens          -----------------------------MRTPIIAGNWKLFKKSSE----AQDFVAELIPLVQK--T-TDVEIVIAPVFTVLSAVKRAIA-DC---N-I
Desulfobacter_postgatei            ----------------------------MTRIPLIAGNWKMYKTGTQ----AVAAAKQLAHLSKG--V-DGVDIMIAPTTLSLPLVAAALG-KS---SPI
Planctomyces_brasiliensis          -----------------------------MRRYLIAGNWKMNTSLET----GTALASGLADHVRGRD--LPVDVLVCPPFPYLAAVKATAGEA-----GI
Spirochaeta_coccoides              -----------------------------MRRYYIAGNWKMNLTPSE----GALYAAELAKAVQKGK--PDAKVMIAPAFVAIPAVLEAVKGS-----PI
Denitrovibrio_acetiphilus          -----------------------------MRKPYIYGNWKMNLDMHG----SSDLISSILS-VKIDY--TKVDVGVAPDFSSLYKVSQTIKQMG---YPI
Prosthecochloris_aestuarii         -----------------------------MRKKIVVGNWKMNKTIAE----AVELSSAIIDQLGEVS--ASCEVGIAPAFPALSGVHGVISGT-----GI
Chlorobium_phaeobacteroides        -----------------------------MRKKIVVGNWKMNKNVAE----GVALASSILENLDGGK--MACEVGIAPAYPVLSEVGRVIEGS-----DI
Chlorobium_ferrooxidans            -----------------------------MRQKIVVGNWKMNNTIAE----SSALASELVAALGKGF--SGCEVGIAPTYVALQEAGKIIAGS-----AV
Chloroherpeton_thalassium          -----------------------------MRKKFVAGNWKMNKDLLG----AVSLATEILQLLGDEA--PTCEVAIAPTFLCQQAVFQVIDES-----AI
Treponema_pallidum                 -----------------------------MRGYFIAGNWKMHKTCAE----AVALAQELVRELRGG----PHTYMIAPSFTALDAVGKVLRGS-----NV
Leptospira_interrogans             -----------------------------MRKTIIAGNWKMNLSLKE----AVFLAHSIREKIPSIS--KDKVSMVFPSTLHLENVSKILEGS-----SV
Borrelia_turicatae                 -----------------------------MRKIFLAGNWKMHYTSME----AAGVAKQIVDGVKNIK--DDVVVMITPPFTSLCKVCEVTRGS-----NV
Thermotoga_maritima                ----------------------------ITRKLILAGNWKMHKTISE----AKKFVSLLVNELHDV---KEFEIVVCPPFTALSEVGEILSGR-----NI
Fusobacterium_nucleatum            -----------------------------MRRLVIAGNWKMYKNNKE----AVETLTQLKDLTRDV---KNVDIVIGAPFTCLSDAVKIVEGS-----NV
Selenomonas_sputigena              ----------------------------MARRPIIAGNWKMNNTIAE----GVALVKELMPLVKDA---KDVDVVACPTATALAAVAEAVKGS-----NI
Ruminococcus_sp                    ----------------------------MARKKIIAGNWKMNMTPSE----AVKLVETLKPLVVN----DEVDVVFCVPAIDIIPVVEATKGT-----NI
Clostridium_botulinum              -----------------------------MRTAIIAGNWKMNKTVKE----AVELVKELKPLVKD----AKCDVVVCPTYVCLPAVLEEVKGS-----NI
Clostridium_perfringens            -----------------------------MRTPIIAGNWKMHYTIDE----AVKLVEELKPLVKD----AKCEVVVCPTFVCLDAVKKAVEGT-----NI
Thermoanaerobacter_wiegelii        ---------------------MISKGVFVLRRPIIAGNWKMHMTPSE----AVKLVEELIPQVKD----AKAEVVVIPPFVDLTEVNKVIQGT-----NI
Brachyspira_murdochii              ----------------------------MARRKLIAGNWKMNNSNKE----ALELVNQLKNLVKDV---KDRDIMIAPTFTCLSDVHNAVKGS-----NI
Bartonella_henselae                --------------------------MSPNIRPFIAGNWKMNGTGES-LGELRAIAAGISSDLGRL-----FEALICVPATLLSRAFDILGGEN-----I
Brucella_abortus                   --------------------------MTPGIRPLVAGNWKMNGKGES-LTELRAIAAGLSSDLGRK-----LDAVICVPATLLSRAAETLEGET-----V
Rhizobium_etli                     --------------------------MTPDVRPLVAGNWKMNGTRAS-LDQIKAIAEGVRPPLADK-----VEALICPPATLLYVATALCTDSP-----L
Agrobacterium_tumefaciens          --------------------------MTPNVRPLVAGNWKMNGTRAS-LDQIKAMAEGVKGALSEK-----VDALICPPSTLLYVATALCDDSP-----L
Rhodobacter_sphaeroides            ------------------------------MRKLAAGNWKMNGTEAD-LGEIDALLA---AHPAPR-----CEMLICPPATLVARMAGR-AAQG-----L
Ruegeria_sp                        -----------------------------MARKLAAGNWKMNGTGEH-LAELENLAQ---GDLPEG-----VDVLICPPATLISRAADR-AGD------I
Bradyrhizobium_japonicum           --------------------------MTDAIRPLIAGNWKMNGLKAS-AAEFDAMLNGA-ADVATK-----ADLLVCPPATLIAAFADKARGKK-----V
Rhodopseudomonas_palustris         -------------------------MPVPAVRSLIAGNWKMNGLKAA-SSEFDAMLEGA-TELSSG-----IDLLVCPPATLIAAFADRLAANEKGALAV
Methylobacterium_radiotolerans     -------------------------MTQSGRRPLVAGNWKMNGTRSS-IQVVEAIRDGLSPDLASR-----IDVLICPPATLIGSCVAAAAGSP-----I
Rhodospirillum_rubrum              ---------------------------MTVRRPLIAGNWKMNGLKAEGQGLARDLAQRLNKAGGVD-----FDLLLCPPFPLISTVVGAVAGSG-----I
Zymomonas_mobilis                  ----------------------------MTIRKLIAGNWKMNG-SLGMLHELDGIAKAT-FNHPE------LDVVICPSFTLIAPA--IVRQPD-----M
Gluconacetobacter_hansenii         ------------------------------MKQIIVGNWKMHGMTADAAKAADDIGAAMGADVSG------AQVVICPPFTQLALLGPKLREHG-----I
Thermus_thermophilus               -----------------------------MRRVLVAGNWKMHKTPSEARVWFAELKRLLPPLQS--------EAAVLPAFPILPVAKEVLAE-----TQV
Thermus_aquaticus                  -----------------------------MRRVLVAGNWKMHKVPSEARVWLAELKRLLPPLDS--------EAAVLPAFPMLPVAKEVLSG-----TPI
Oceanithermus_profundus            -----------------------------MRTPLLAGNWKMHKTPSEARVWFAEFLEANPTLQSGGQ--AAVEVALLPSFPLVPVAAEQLAG-----SGV
Deinococcus_deserti                -----------------------------MPNNLLALNWKMNKTPSEAQAWAAELSSALSPGEA--------ELAVMAPAIMLPALAAGLP------AGV
Bifidobacterium_bifidum            --------------------------MAS-RIPLLAGNWKMNLDHLEATYFVQKLVWLLRDARFDY---SRCEIALMPSFTSLRSVQVLVEADK---LKI
```

```
Bifidobacterium_longum            --------------------------MASKRIPLVAGNWKMNFDHLEATYFVQKLVWLLRDAHFDF---KRCEVALFPSFTSLRSVQVLVEADK---LHV
Gardnerella_vaginalis             --------------------------MVVNRKPLVAGNWKMNFNHLEATHFIQKFWWRLCDSHFDC---KKCDIALMPSFTSLRSVQVLVESDN---LPI
Nocardia_farcinica                ----------------------------MARKPLIAGNWKMNLNHLEAIALVQKIAFALPEKYFE-----KVDVAVIPPFVDIRSVQTLVEGDK---LLL
Mycobacterium_tuberculosis        ----------------------------MSRKPLIAGNWKMNLNHYEAIALVQKIAFSLPDKYYD-----RVDVAVIPPFTDLRSVQTLVDGDK---LRL
Corynebacterium_glutamicum        ----------------------------MARKPLIAGNWKMNLDHQQAIGTVQKLAFALPKEYFE-----KVDVAVTVPFTDIRSVQTLVEGDK---LEV
Propionibacterium_acnes           ----------------------------MSRTPIMAGNWKMNLDHVAATSLVQDLGEALKAAGYDS---SKSEAVVIPPFTDIRPVAIIIDGDK---LPI
Actinomyces_odontolyticus         ----------------------------------MAGNWKMNLDHLEANHLVQGLAMALSDAGHDY---SKCEVLVIPPFTDIRTVQTIVDADE---LGI
Microbacterium_testaceum          --------------------------MGVTRTPLIAGNWKMNLDHLQAVAFVQKLHWTLKEAKHEN---GSVEVAVFPPFTDLRTVQTLLDADK---IEF
Moritella_marina                  -----------------------------MRHPVVMGNWKLNGSKEM----VVDLLNGLNAELEG-V--TGVDVAVAPPALFVDLAERTLTEA---GSAI
Photobacterium_profundum          -----------------------------MRHPVVMGNWKLNGSKEM----VVNLLNGLNAELEG-V--TGVDVAVAPPALFIDLAEHTLTEA---GSAI
Vibrio_cholerae                   ---------------------MIRYGDIPMRRPVVMGNWKLNGSKAM----VTDLLNGLNAELEG-V--EGVDVVVAPPAMYLDLAERLIKEG---GNKL
Shigella_flexneri                 -----------------------------MRHPLVMGNWKLNGSRHM----VHELVSNLRKELAG-V--AGCAVAIAPPEMYIDMAKREAEG-----SHI
Escherichia_coli                  -----------------------------MRHPLVMGNWKLNGSRHM----VHELVSNLRKELAG-V--AGCAVAIAPPEMYIDMAKREAEG-----SHI
Salmonella_enterica               -----------------------------MRHPLVMGNWKLNGSRHM----VNELVANLRKELTG-V--AGCDVAIAPPEMYIDLAKRAAAG-----SHI
Klebsiella_pneumoniae             -----------------------------MRHPLVMGNWKLNGSRHM----VNELVANLRTELAG-V--SGCAVAIAPPEMYLDLAKRAAEG-----SHI
Enterobacter_aerogenes            -----------------------------MRHPLVMGNWKLNGSRHM----VNELVANLRTELAG-V--SGCAVAIAPPEMYLDLAKRAAEG-----SHI
Serratia_symbiotica               -----------------------------MRHPLVMGNWKLKGSTHM----VSELIAGLRNALSR-V--DGCGVAIAPPVMYLDQARHALAG-----SRI
Yersinia_pestis                   -----------------------------MRHPLVMGNWKLNGSTHM----VNELIAGLRKELST-V--DGCGVAIAPPAIYLNQAKHELAG-----SRI
Actinobacillus_pleuropneumonia    ----------------------------MARRPLVMGNWKLNGSKAF----TKELIAGLKAELAD-V--KGCDVAIAPPVMYLAEAEAALAGQ----SVI
Pseudomonas_aeruginosa            -----------------------------MRRPLVAGNWKMHGTHSS----VAELIKGLR-QLAL-P--SGVDVAVMPPCLFISQVIQGLAG-----KAI
Marinomonas_posidonica            ----------------------------MIRQKIVAGNWKMNGSKAS----ISSLVNGLLEMSE-----SSAEVVVTPPFPYLSQVADVIAQQ----SGL
Xylella_fastidiosa                -----------------------------MRPKIVAGNWKLHGSHAF----AQALVAQVAAGLP--L--LGVSVIILPPLLYLSDLAQRFKG-----EGL
Xanthomonas_campestris            -----------------------------MRRKIVAGNWKLHGTRAF----ATELVAQVAAHMP--L--AGVDVVILPPLPYLGDLIEDFEA-----HHL
Marinobacter_algicola             -----------------------------MRRKIVAGNWKMNGSKGL----VSNLVGQVRSELASFD--NGVEAVIIPPALFVDDVVEQA-G-----KKL
Aeromonas_caviae                  ---------------------------MGHRKPFVAANWKLHGSRSQ----LEQFTSQCAERI---D--EDVDVVLCPSHVHLGFTAELLGEQG----RV
Pseudoalteromonas_haloplanktis    ---------------------------MAARKAMVAGNWKMNGSLEL----VKQMSDAINNVK---S--NEIDIVLFP---PFPLVSAMIASG------V
Legionella_pneumophila            -----------------------------MRQKIVAGNWKMNGQIQQ----VTELVSQIEELIGF-D--CAAQVAVMPPSIYIPKVRDCLRTG-----RI
Ralstonia_solanacearum            -------------------------MS--ARPKLVVGNWKLHGSLNG----NAELLEKIKAAG---Q--ARAALAVCAPFPYLAQCQSLLAGS-----AV
Burkholderia_sp                   -------------------------MSNQQRAKLVVGNWKMHGRLAA----NAALLQAVVQGANELP--ADVRVGVCVPSLYLAQTQSLLEGT-----SV
Bordetella_pertussis              ------------------------MTTAENRARLVLGNWKMHGNLAE----NAALLAELRAAD--AA--AHCEMGVCVPFPYLAQTAAALQGS-----AI
Nitrosococcus_watsonii            -----------------------------MRTSLVVGNWKMNGSRAA----NRALLESMRKEM-EAG--VVAEVAVCPPFVYLADMESLLQGS-----VI
Coxiella_burnetii                 ----------------------------MQRRPLVAGNWKMHGSRES----VGQLLRALKHGC-ERL--ETAELAVFPPFVFLQQCEEALMRT-----QI
Variovorax_paradoxus              -------------------MVTTTNNAMTTKKKLIAGNWKMNGGLAV----NEALVKALQQGLAASP--AACDVALCAPAPYFAQLQSLLAGTP----SL
Halomonas_elongata                -----------------------------MPTPLIAGNWKMNGSLDL----IESFGRALADAE--LP--SRLEVALMVPFPYLEAASRALTGS-----RA
Sphaerobacter_thermophilus        -----------------------------MRKPIVAGNWKMNTSLEEARALAGELRSTL-SAY-ST-----VERVLIPPFPWIVPLADILDG-----SDI
Anaerolinea_thermophila           -----------------------------MRKKFVAGNWKMNKTAEEARLLLNELIPAL-EPF-SH-----VERAVCPPFPYLMMVRHMLEG-----TNI
Myxococcus_xanthus                -------------------------MATARRRKIVAGNWKMNKSVPEALALVRDLRGQV-ASLGDT-----VEVVVAPPFVALQPLHVALEG-----APL
Roseiflexus_castenholzii          -----------------------------MRTPLLAGNWKMYKTTGEARELVEGLLHGLGDVGDR-------KVLVCPPFTALQTVHDLVQG-----TPI
Chloroflexus_aurantiacus          -----------------------------MRIPLIAGNWKMYKTVGEATTLVRDLLAGLGELSDR-------EAIVCPPFTALAAVAALVAD-----SPL
Collinsella_tanakaei              -------------------------MTQNNRTLLIAGNWKMNNDVQAAATLADELVAALDGVDNG------VEVLVCPPTIDLTTVAAKLDG-----SAI
Atopobium_vaginae                 -----------------------------MRNTMIAGNWKMNKTYGEAAELAQGLVRELPGSTGN------VDVVVCPPTIDLKAVAATLEQSD---SSI
Eggerthella_sp                    -----------------------------MRKPMMAGNWKMNNTVAEAVVLTQELSNRYTDAWADR-----ADVVICPPFVDLKPAKTVLEFDK---TRI
Ostreococcus_lucimarinus          ----------------------------------MAGNWKLNPKTLDEARTLAALVGAAARDGGIGTNKSKTEVFVCPPAPFAAEVARTLEGSG-----V
Lentisphaera_araneosa             ----------------------------MKRRLFIAGNWKLNKTAAETTETVADLKAKLADFNGS------LDVALFPPFLSIAAGQAAAAGSP-----I
Acholeplasma_laidlawii            ---------------------------MAKRIPVIAANWKMFKTKDE----ALEFIFAVNAAVPSRD---EVESIICAPAILLNLLVKREGE------HI
Lactobacillus_reuteri             -----------------------------MRKPFIAANWKMHKNVQE----SVEFVDAIKGKLPDPQ---EVEVGIAAQAFALPSMVQAADDS-----GL
Bacillus_subtilis                 -----------------------------MRKPIIAGNWKMNKTLGE----AVSFVEEVKSSIPAAD---KAEAVVCAPALFLEKLASAVKG-----TDL
Bacillus_thuringiensis            -----------------------------MRKPIIAGNWKMNKTLSE----AVSFVEEVKGQIPAAS---AVDAVVCSPALFLERLVAATEG-----TDL
Listeria_monocytogenes            -----------------------------MRKPIIAGNWKMNKTAAK----AGQFAEDVKNNVPSSD---AVESVVAAPALFLQELVRLTEG-----TNL
Geobacillus_stearothermophilus    -----------------------------MRKPIIAGNWKMHKTLAE----AVQFVEDVKGHVPPAD---EVISVVCAPFLFLDRLVQAADG-----TDL
Staphylococcus_aureus             -----------------------------MRTPIIAGNWKMNKTVQE----AKDFVNALP-TLPDSK---EVESVICAPAIQLDALTTAVKEGK--AQGL
Enterococcus_faecalis             -----------------------------MRKPIIAGNWKMNKTLSE----AQSFAEAVKNAVPSND---VVDAVIGSPALFLAPLAWNLKD-----SEV
Lactococcus_lactis                ----------------------------MSRKPIIAGNWKMNKTLSE----AQAFVEAVKNNLPSSD---NVESVIGAPALFLAPMAYLRQG-----SEL
Streptococcus_pneumoniae          ----------------------------MSRKPFIAGNWKMNKNPEE----AKAFVEAVVSKLPSSD---LVEAGIAAPALDLTTVLAVAKG-----SNL
Erysipelotrichaceae_bacterium     -----------------------------MRKPIIVGNWKMNKTMKE----TKEFMEAVDAAAASEN---AVFG-IGAPYTALSAAVAGAKN-------L
Tenebrio_Molitor                  ----------------------------MARKFVVGGNWKMNGDKKQ----INEIIG-FLKSGPLNQD---TEVVVGVPAIYLELVRTCVP------ASI
Bombyx_mori                       ----------------------------MGRKFVVGGNWKMNGDKNQ----INEIVN-NLKKGPLDPN---VEVIVGVPAIYLSYVKTIIP------DNV
```

```
Apis_mellifera                  ----------------------------MGRKFFVGGNWKMNGTKSE----INDIVG-FLKKGPLDSN---VEVVVGVPSIYLTYAKNILP------NNI
Aedes_aegypti                   ----------------------------MGRKFCVGGNWKMNGDKAS----ITDLCK-TLTTGPLSAD---TEVIVGCPAPYLTLARSQLP------DSV
Anopheles_gambiae               ----------------------------MGRKFCVGGNWKMNGDKAS----ITELCK-TLSAGPLDPN---TEVVVGCPAPYLSLARSLLP------ETI
Drosophila_melanogaster         ----------------------------MSRKFCVGGNWKMNGDQKS----IAEIAK-TLSSAALDPN---TEVVIGCPAIYLMYARNLLP------CEL
Rhipicephalus_microplus         ---------------------------MAARRFCVGGNWKMHGSKNS----IRDICN-TLKGASLDPN---VEVIVACPAPYLDYCRSLLP------PSV
Ixodes_scapularis               ---------------------------MSGRKFCVGGNWKMNGNKSS----IKEICD-MLKTAKLDPN---TEVVLGCPAPYLDYVRRILP------AAI
Anolis_carolinensis             --------------------------MAP-RKFFVGGNWKMNGDKKS----LGELIQ-TLNGAKVSGD---VEVVCGAPTIYLDFARQKLD------AKF
Gallus_gallus                   --------------------------MAP-RKFFVGGNWKMNGDKKS----LGELIH-TLNGAKLSAD---TEVVCGAPSIYLDFARQKLD------AKI
Canis_lupusfamiliaris           --------------------------MAPSRKFFVGGNWKMNGRKKN----LGELIT-TLNAAKVPAD---TEVVCAPPTAYIDFARQKLD------AKI
Oryctolagus_cuniculus           ---------------------------APSRKFFVGGNWKMNGRKKN----LGELIT-TLNAAKVPAD---TEVVCAPPTAYIDFARQKLD------PKI
Pan_troglodytes                 --------------------------MAPSRKFFVGGNWKMNGRKQS----LGELIG-TLNAAKVPAD---TEVVCAPPTAYIDFARQKLD------PKI
Homo_sapiens                    --------------------------MAPSRKFFVGGNWKMNGRKQS----LGELIG-TLNAAKVPAD---TEVVCAPPTAYIDFARQKLD------PKI
Nomascus_leucogenys             --------------------------MAPSRKFFVGGNWKMNGRKQS----LGELIG-TLNAAKVPAD---TEVVCAPPTAYIDFARQKLD------PKI
Pongo_abelii                    --------------------------MAPSRKFFVGGNWKMNGRKQS----LGELIG-TLNAAKVPAD---TEVVCAPPTAYIDFARQKLD------PKI
Macaca_mulatta                  --------------------------MAPSRKFFVGGNWKMNGRKQN----LGELIG-TLNAAKVPAD---TEVVCAPPTAYIDFARQKLD------PKI
Bos_taurus                      --------------------------MAPSRKFFVGGNWKMNGRKNN----LGELIN-TLNAAKVPAD---TEVVCAPPTAYIDFARQKLD------PKI
Sus_scrofa                      --------------------------MAPARKFFVGGNWKMNGRKNN----LGELIN-TLNAAKLPAD---TEVVCAPPTAYIDFARQKLD------PKI
Mus_musculus                    --------------------------MAPTRKFFVGGNWKMNGRKKC----LGELIC-TLNAANVPAG---TEVVCAPPTAYIDFARQKLD------PKI
Rattus_norvegicus               --------------------------MAPSRKFFVGGNWKMNGRKKC----LGELIC-TLNAAKLPAD---TEVVCAPPTAYIDFARQKLD------PKI
Xenopus_laevis                  --------------------------MSP-RKFFVGGNWKMNGDKKS----LGELIN-TLNSGKMNAD---TEVVCGAPAIYLDFARQKLD------AKI
Danio_rerio                     ---------------------------MSGRKFFVGGNWKMNGDKKS----IEELAN-TLNSAKLNPD---TEVVCGAPTIYLDYARSKLN------PNI
Ictalurus_punctatus             ---------------------------MTARKFFVGGNWKMNGDKKS----LGELIN-TLNGAKLNAD---TDVVCGAPSIYLDFARSKLD------AKI
Oreochromis_niloticus           ---------------------------MT-RKFFVGGNWKMNGDKKS----LGELIQ-TMNGAKVDPN---VEVVCGAPSIYLDFVRSKLD------PKF
Nematostella_vectensis          --------------------------MG--RKFFVGGNWKMNGSLVQ----IDGILK-NLHDSSLSDD---TEIVVSPPALYLSYVRKAAK------QNI
Schistosoma_mansoni             --------------------------MSGSRKFFVGGNWKMNGSRDD----NDKLLK-LLSEAHFDDN---TEVLIAPPSVFLHEIRKSLK------KEI
Strongylocentrotus_purpuratus   --------------------------MASGRRFWVGGNYKMNGSKAS----IDGLLK-MLEESQIPGN---TDVVVAPPTVYLDYMNSKIP------AGV
Caenorhabditis_elegans          ----------------------------MTRKFFVGGNWKMNGDYAS----VDGIVT-FLNASADNSS---VDVVVAPPAPYLAYAKSKLK------AGV
Brugia_malayi                   ----------------------------MSRKFLVGGNWKMNGNKAS----VDNIIK-FLNDGAVVPN---VDVVVAPPAPYLSYVKEKVK------NGI
Ricinus_communis                ----------------------------MARKFFVGGNWKCNGTSEE----VKKIVS-TLNEGHVPSSD-VVEVVISPPFVFLPLVKDSLK------PDF
Vitis_vinifera                  ----------------------------MGRKFFVGGNWKCNGTGEE----VKKIVS-TLNAGEVPSGD-VVEVVVSPPFVFLPLVKSTLR------PDF
Arabidopsis_thaliana            ----------------------------MARKFFVGGNWKCNGTAEE----VKKIVN-TLNEAQVPSQD-VVEVVVSPPYVFLPLVKSTLR------SDF
Zea_mays                        ----------------------------MGRKFFVGGNWKCNGTTDQ----VEKIVK-TLNEGQVPPSD-VVEVVVSPPYVFLPVVKSQLR------QEF
Oryza_sativa                    ----------------------------MGRKFFVGGNWKCNGTTDQ----VDKIVK-ILNEGQIASTD-VVEVVVSPPYVFLPVVKSQLR------PEI
Glycine_max                     ----------------------------MGRKFFVGGNWKCNGTTEE----VKKIVT-TLNEAKVPGED-VVEVVVSPPFVFLPFVKSLLR------PDF
Perkinsus_marinus               ----------------------------MSPKPFVGGNWKSNGSVES----IKKLAP-ALVAGEATYNKDAVDVVIAPTAIHLPLAMEQFKG-----SNI
Chlamydomonas_reinhardtii       MQLCKVQRASAARSSRASRSQRVEVVCASSAKFFVGGNWKCNGSVAN----VAKLVD-ELNAGTIPRG---VDVVVAPPFIYIDYVMQHLDR-----DKY
Trypanosoma_brucei              ---------------------------MSKPQPIAAANWKCNGSQQS----LSELID-LFNSTSINHD---VQCVVASTFVHLAMTKERLSH-----PKF
Trypanosoma_cruzi               --------------------------MASKPQPIAAANWKCNGSESL----LVPLIE-TLNAATFDHD---VQCVVAPTFLHIPMTKARLTN-----PKF
Leishmania_mexicana             --------------------------MSAKPQPIAAANWKCNGTTAS----IEKLVQ-VFNEHTISHD---VQCVVAPTFVHIPLVQAKLRN-----PKY
Entamoeba_histolytica           ---------------------------MSARKFVVGGNWKCNGTLAS----IETLTK-GVAASVDAELAKKVEVIVGVPFIYIPKVQQILAGEAN-GANI
Plasmodium_falciparum           ----------------------------MARKYFVAANWKCNGTLES----IKSLTN-SFNNLDFDPS--KLDVVVFPVSVHYDHTRKLL------QSKF
Toxoplasma_gondii               ----------------------------MVRTPWVGGNWKCNGTVGS----ITDLCG-EFGKTEFDPK--TIDVVIFPPALHAPLTREKL------PKKY
Cryptosporidium_parvum          ----------------------------MSRKYFVGGNFKCNGTKES----LKTLID-SFKQVESSNS----EVYVFPTSLHISLVKEFFGNDH--PGVF
Paramecium_tetraurelia          -----------------------------MRRYFVGGNWKCNNTIAQ----TQSLINTVINKLVFDVN--KVEVAVAPIFLHVPWVQANI------QKNV
Aspergillus_niger               ----------------------------MPRQFFVGGNFKMNGTADS----ITSIIK-NLNAAKLDE---SAEVVVSPPTLYLLPARQAAG------EKI
Paracoccidioides_brasiliensis   ----------------------------MPRKFFVGGNFKMNGTAKS----ITHIIT-NLNSAKLDP---STEIVIAPPAIYLVLARQLAN------GQV
Schizosaccharomyces_pombe       ----------------------------MARKFFVGGNFKMNGSLES----MKTIIE-GLNTTKLNVG--DVETVIFPQNMYLITTRQQVK------KDI
Saccharomyces_cerevisiae        ----------------------------MARTFFVGGNFKLNGSKQS----IKEIVE-RLNTASIPE---NVEVVICPPATYLDYSVSLVKK-----PQV
Kluyveromyces_lactis            ----------------------------MARTFFIGGNFKMNGSKAS----IKEIVD-RLNGASIPS---NVEVVIAPPAIYLDHAVALNKR-----KEV
Candida_dubliniensis            ----------------------------MARQFFVGGNFKANGTKQQ----ITSIID-NLNKADLPK---DVEVVICPPALYVGLAVEQNKQ-----PTV
Giardia_lamblia                 ---------------------------MPARRPFIGGNFKCNGSLDF----IKSHVA-AIAAHKIPD---SVDVVIAPSAVHLSTAIAANTS-----KQL
Trichomonas_vaginalis           -----------------------------MRTFFVGGNWKANPKTVE----EAEKLIEMLNGAKVEGN---VEVVVAAPFIFLPTLQQKLR------KDW
Sphingobacterium_sp             -----------------------------MRKKIVAGNWKMNMDYQE----GISLFSEVANMVKDEVIG-NQQIIVCSPFIHLHSIASLAKESE----NV
Pedobacter_saltans              -----------------------------MRKKIVAGNWKMNKDYSE----GLSLFSEVLNMVKDEVRG-EQQVVVCAPYIHLHSLSALAKGTN----NV
Marivirga_tractuosa             -----------------------------MRQKIVAGNWKMNKTLVE----AQSLTSEIANMVEDEAPS-DVKVVIAPPAPFIYAVENLIPEMG----QI
Cyclobacterium_marinum          -----------------------------MRKKIVAGNWKMNCLLEE----GQKLTSEIVNMIKDEPIK-DVKVILNPPFVHLHGVKKLIAGVD----NI
Leadbetterella_byssophila       -----------------------------MRKKYAAGNWKMNKTFEE----GQILLSEVVNMVKDELTHPNVQVVLGVPFPYLSTFSKLVDTP-----KV
```

```
Haliscomenobacter_hydrossis      ---------------------------MKTRQQIVAGNWKMNKNYGE----GRELAMEIVERLK---PS-NTQVVLCAPYIHLQLVKNIIKDVA----SL
Parabacteroides_distasonis       -----------------------------MRKNIVAGNWKMNTTLAE----GLALAKGLDEALKGKTPN--CDVIIGTPFTHLASVAAAIDTN-----KI
Bacteroides_fragilis             -----------------------------MRKNIVAGNWKMNKTLQE----GIALAKELNEALANEKPN--CDVIICTPFIHLASVTPLVDAA-----KI
Prevotella_ruminicola            -----------------------------MRKKIVAGNWKMNMNLQD----GIALAKELNETLKADKPN--CGVVICTPFIHLASIAQFLDQD-----II
Alistipes_shahii                 -----------------------------MRKKIVAGNWKMNTLPAE----GVELAKGVVAGRGEVCSC--VNFIVCPPFTHLAMVAEALKGS-----DV
Porphyromonas_asaccharolytica    ---------------------------MDKRTLIVAGNWKMNMTLDA----GLRLITDIKEQVAKLSNP--CKVILAPPYIHLGAMKGLLAGS-----DI
Cellulophaga_lytica              -----------------------------MRAKIVAGNWKMNKTLTE----TNALLAELSGKLP----DTDAEVMVAPTYVNLAAAVEDVKSS-----TI
Kordia_algicida                  -----------------------------MRRKIVAGNWKMNNDLSE----TEALLNDLIEQVP----NTDASVMVAPAFVNLFQAFNVLKTF-----NV
Flavobacterium_psychrophilum     -----------------------------MRAKIVAGNWKMNKNSEE----TEDLINELIDKLPT---NSNAKIIIAPTFINLASAVDHTEFT-----NI
Neisseria_gonorrhoeae            ----------------------MYRQIGMWDQKWVIGNWKMNGRLQN----NNALMHRF-RILPTAER---VLIGLAAPTVYLLQLHNAMQIVLNN--RI
Francisella_tularensis           ------------------------------MQKLIMGNWKMNGNSTS----IKELCSGISQVQYDTSR---VAIAVFPSSVYVKEVISQLPEKVG-----
Mycoplasma_genitalium            -----------------------------MRTRYLIGNWKTNKNLKD----AVSFVEQFQQNKLNYN----AKIGIAPVYVHLTEIKKIISDS------L
Ureaplasma_urealyticum           ----------------------------MVKMKYIIANFKMNATEEL-------INHFLNNLISFDE---QKLTIGLAPGDLYLKTFVDLSQTK----KV
Ureaplasma_parvum                -------------------------------MKYIIANFKMNATQEL-------INHFLNNLTLFDE---QKIIIGLAPGDLYLKTFVNLAEIK----KV
Buchnera_aphidicola              -----------------------------MKKFFITANWKLNGNIKM-------ISSFFKYLKLYSSSYLEKNTVIIAPPTIYLERVCKNISNM----NI
Campylobacter_jejuni             --------------------------------MIFAANLKCNHTRAS-------FKIYAEILNKTIG-VKCDDIIVFPP-----SVAFLEKEN-----NF
Helicobacter_pylori              ------------------------------MTKIAMANFKSAMPIFK-------SHAYLKELEKTLKPQHFDMVFVFPD-----FLGLLPNSFL----HF
Orientia_tsutsugamushi           ------------------------------MESIVVSNWKMHFSFSE-------ACNYLNLITSLNSNLNLAKMIFAVPNLYLSGLKLKFNDTY----HF
Wolbachia_sp                     ------------------------------MSFLIVANWKMNGMRSS-------FVDFIGKLNNKSNEITSKLVICPPFT----SFPSSIELNN----NI
Methanocaldococcus_Jannaschii    --------------------------------MVIVINYKTY-NESIG-NRGLEIAKIAEKVSEESG----ITIGVAPQFVDLRMIVENVNIP-------
Methanococcus_voltae             -----------------------------MIKPVVIINYKTY-MESIS-DKGLNIAKCAEKVSEESG----IEISVAPQFTDLKTIVEQTNVK-------
Methanothermococcus_okinawensi   -----------------------------MIKPIIIINYKTY-AESVG-EKGLKIAKAAEKVSEESG----ISIGVCPQFLDLRMISENVNIP-------
Pyrococcus_Woesei                --------------------------MAKLKEPIIAINFKTY-IEATG-KRALEIAKAAEKVYKETG----VTIVVAPQLVDLRMIAESVEIP-------
Pyrococcus_furiosus              --------------------------MAKLKEPIIAINFKTY-IEATG-KRALEIAKAAEKVYKETG----VTIVVAPQLVDLRMIAESVEIP-------
Thermofilum_pendens              ---------------------------MKIGYPLILINFKAY-SEASG-KRGLQLAKVAEKVSKETG----ITIAVAPQLTDLAFIASQVEIP-------
Methanothermobacter_thermautot   ----------------------MLEDLELKDTPIVILNFKTY-LESTG-ERALELASICGDVADETG----VNMAVAPQHMDLHRVSDAVEIP-------
Methanosaeta_thermophila         --------------------MRWSQSLGGMALTLIVLNFKTY-REATG-DSAVALSKICESIASEYG----VDIAVAPQAADIRAVASAVSIP-------
Natronobacterium_gregoryi        --------------------------------MFVLVNLKTY-PCDP-----LEIATAVRDVDETTD----ARLAVAPQAIHIERVAET-GVE-------
Halobacterium_salinarum          --------------------------------MFVLVNLKAY-PCDP-----VAIAEAAADVAETTP----ATIAVAPQPADIGRVADT-GAT-------
Halogeometricum_borinquense      --------------------------------MFILVNLKAY-PCDP-----LEVATAAHEVAEESG----VRIAVAPQAADVRRVADT-GVE-------
Methanosphaerula_palustris       -----------------------------MSSQFILVNLKAY-KEGMG-PRAHMIARAAEEVSEESG----IQIGVAPSFFDLHPLSKHFEIP-------
Thermoproteus_tenax              -----------------------------MRLPILIINFKAY-GEAAG-KRAVELAKAAERAARELG----VNIVVAPNHLELGLVSQSVDIP-------
Pyrobaculum_islandicum           -----------------------------MKFPILIINLKAY-GEAAG-KKALEIAKAAEKVAKELG----VNIAVAPNHLELALVAQSVEIP-------
Cenarchaeum_symbiosum            ---------------------------------MLIINCKNY-KEAAG-GRIDSLAAAAAGAAAKYG----VRIALAPPQHLLGAVKGEDLT--------
Nitrosopumilus_maritimus         ---------------------------------MFVINCKNY-EEISG-EKITKFVKTAEKVSKKFK----VKIAICPPQHLIGVVANSSIP--------
Desulfurococcus_kamchatkensis    ----------------------------MVKP-VLAVNFKAYYPHSFG-ENAYRLAKDAVRVWRETG----VEVILAPPFTELKSVIEAVKDTG-----V
Pyrolobus_fumarii                ----------------------------MRDVYILAVNFKVY-PSAFG-RRALEVVKAAERVAREFEG--TVSVIIAPPHTEIARISSSVER-------V
Acidilobus_saccharovorans        ----------------------------MPRL-VFAVNFKAY-ETAFN-EKSLEIAREASKASSRYG---NVRVILIVPAAIASKVLQIYDD--------
Sulfolobus_islandicus            ----------------------------MKPP-IIVINFKAY-ENSFG-NKAIELGKKIEKISKEYS----VEIILSVPATMIYRMVQEVDLP-------
Clustal Consensus                                                     . *

                                         110       120       130       140       150       160       170       180       190       200
                                 ....|....|....|....|....|....|....|....|....|....|....|....|....|....|....|....|....|....|....|....|
Dictyoglomus_thermophilum        KLGAQNMFWE-KEGAYTGEISPIMLKDLNCTYVIIGHSERRNYFSETNEMINKKIKSAFNYGLI-PIFCVGEKWEERERGKTEEVITKQVREGLEGLEK-
Aquifex_aeolicus                 KLGAQNCHYE-KRGAFTGEISIPMLQEVGCEYVIVGHSERRHIFGESDELIHKKIVACLEMGIR-PILCVGEKKEEREAGMTFKVIETQIKLALTGVE--
Thermodesulfovibrio_yellowston   KLCAQNAFYE-NKGAYTGEVSPAMLKDCGVEYVIIGHSERRKYFYENDDIINKKIHACIKEGLK-VIFCIGETFEDRQNNKTMEILKTQIRNGLLEIN--
Dehalogenimonas_lykanthroporep   QLGAQNMYQE-AKGAFTGEISPVMLQDF-CKYVIIGHSERREIFQETDDMIKLKVRAALKFGLK-PILCVGENLAQREGGKVQEVIEGQITKALDGVN--
Dehalococcoides_sp               KLGAQNLFYQ-EKGAYTGEISPLMLKEL-CQYVIIGHSERRAYFGETGQVVNQKIKAALQAGLL-PIVCVGEKPEENENGQTRQVLETQLKEALDGLN--
Nostoc_punctiforme               QLGAQNIHWE-EFGAYTGEISGPMLTESGVRFVIVGHSERRQYFGETDATVNLRLRTAQRFGLT-PILCVGETKQQRDAGETESLIALQLDKGLVDID--
Anabaena_variabilis              QLGAQNVHWA-ENGAYTGEISGPMLTEIGVRYVIVGHSERRQFFGETDETVNLRLQAAQKYGLT-PILCVGETKQQRDSGETESLIVSQLDKDLINVD--
Trichodesmium_erythraeum         RLGAQNVHWE-DEGAYTGEISGLMLKDVGVSYVIVGHSERRQYFGETDETVNMRLKAAQKHGLT-PILCVGETKQQRDSGETESHIFSQLANDLVDVD--
Crocosphaera_watsonii            RLGAQNVHWE-DKGAYTGEISGPMLTELSVDYVVIGHSERRQYFSETDETANLRVITAQRHGLT-PILCVGESKQQRDAGEAESVIINQLQKGLVDVD--
Synechocystis_sp                 RLGAQNVHWE-ASGAYTGEISAAMLTEIGIHYVVIGHSERRQYFGETDETANLRVLAAQKAGLI-PILCVGESKAQRDAGETEQVIVDQVKKGLVNVD--
Synechococcus_sp                 QVGAQNVHWE-REGAYTGEVSGVMLKELGVRYVVVGHSERRQYFGETDETVNARLKAAQLHGLT-PILCVGETKQQRDADQTESVITGQIKKALVDIN--
Cyanobacterium_UCYN              HLGAQNIHWA-NEGAFTGEISGSMLVDLNANYVIVGHSERRQYFGETDKIVNEKVIAAQYHKIK-PILCVGETKKQRDAGEVEKVIVKQLEQSLVNVD--
Prochlorococcus_marinus          SLSSQNVHWE-DNGAFTAEISPKMLLEHSVSYAIVGHSEPRKYFSESDKQINLRAKSAQANGLI-PIVCVGETIEQRERGEAERVIRRQVEQGLEETD--
Chlamydophila_pneumoniae         WLGAQNVHPE-LSGAFTGEISLPMLKEVGVEFVLVGHSERRHIFGESDAFIASKVKSVAQAGLV-PVLCVGESLEVREEGKAHQVIKKQLLLGLEQMDN-
```

```
Desulfovibrio_vulgaris          -IGGQDVYPA-TEGAYTGEIAPGMLLDAGCGWVLTGHSERRHILGEDDETVARKTAFSLKAGLR-VVLCIGEKLDEREAGRLEDVLAHQLQVGLADVDAT
Mesorhizobium_opportunistum     --GAQNMHWA-DAGAWTGEISPVMLTDCGLDLVELGHSERREHFGETDRTVGLKTAAAVKHGLI-PLICVGETLAERESGEADAVLAKQVEGALQFFEEE
Geobacter_metallireducens       MLSAQDCFWE-EEGAFTGEISPGMLVDAGCSHVIIGHSERRQYFGETDETVNRKIKAAITAGLT-VLFCIGETLAEREADKTFEVLRTQIENGLAGLARG
Desulfobacter_postgatei         RLGAQNIYPG-TEGAFTGEVSGPMIKDAGADYVIIGHSERRQYFGETDESVRLKIRSALDAGLI-PVMCIGETESQRDADETFFILDKQITDGLKGFDLG
Planctomyces_brasiliensis       SVGAQNCYFE-ASGAFTGEVSVDMLKDIGCDSVILGHSERRHVIKECDDMINKKTKAAIEGGLQ-VVLCVGELLEEREADKTEAVLDEQMAGGLKDISA-
Spirochaeta_coccoides           IVAAQNAADH-LSGAYTGEVSLSMLKDLGVGTVILGHSERRAIYGESDALINAKTKLALSEGFE-VILCVGETLEEREAGKLEDVLSRQVKIGLGGVSS-
Denitrovibrio_acetiphilus       SVAAQNISAE-EKGAFTGDVSAAMVFAAGADSTILGHSERRMIFAEGDELINQKVKTALRNNLD-VILCVGETLDERESGVAADRVVYQVGMGLKDIGM-
Prosthecochloris_aestuarii      HLAAQNCHYE-DDGAFTGEVSVRMLDEAGCSYVIVGHSERRQYFGDTNPVVNLKVKKALSAGLN-VILCVGETLDEREKGITTEVVTCQVKEGLEGV---
Chlorobium_phaeobacteroides     CLVAQNCHYE-DEGAYTGEVSVRMLDSLGCSYIIVGHSERRQYFGETNRTVNLRLKKALEGGMR-VILCIGETLEEREAGVTDAIVTAQVREGLADI---
Chlorobium_ferrooxidans         QLVAQNCHYE-NDGAFTGEISTGMLKAVGCSYVIIGHSERRQFFGETNATVNLRIKKALAEGLK-VILCVGETLAERESEVTSKVVTSQVTEGLAGI---
Chloroherpeton_thalassium       KLAAQNCFYE-DQGAYTGEISAAMLRNSGCEYVILGHSERRQYFNETDEIVNKKVKNALSVELD-VIMCVGETLEQRESGVTKSVVETQVRGGLKDLTA-
Treponema_pallidum              LLGAQDVSSE-EWGAHTGEVSVLQLEDLGVQVVIVGHSERRHGRGENDKLINQKVRRVLESGLR-VILCVGERLQEYEAGCTNEVVGTQVRAGMADVCG-
Leptospira_interrogans          IVGAQNCYHS-GLAAFTGETSPDQLKEIGVKVVMVGHSERRQFLGESNFFCNDKIRFLLKNEFT-VLYCVGETLSERESGKTLEVLSSQIREGLKGIDS-
Borrelia_turicatae              LLGAQNMSYE-DSGARTSEISPSMLLEFGVDYVILGHSECRTYLGDTDEVVNKKVLAGLKHPFKYLILCIGETLEERENNKTLDVVLNQIRKGLISVSE-
Thermotoga_maritima             KLGAQNVFYE-DQGAFTGEISPLMLQEIGVEYVIVGHSERRRIFKEDDEFINRKVKAVLEKGMT-PILCVGETLEEREKGLTFCVVEKQVREGFYGLDK-
Fusobacterium_nucleatum         KIAAENVYPK-IEGAYTGEVSPKMLKDIGVTYVILGHSERREYFKESDEFINQKVKAVLEIGMK-PILCIGEKLEDREGGKTLEVLAKQIKEGLVDLSK-
Selenomonas_sputigena           HIGAQNVHWE-KSGAYTGEISTDMLKEIGVDYCVLGHSERREYFGETDEGVNKRAKAAFAAGIT-PIICCGEPLEIREANTYIDFVTKQIKAALDGFSA-
Ruminococcus_sp                 QVGAENMYFE-EKGAYTGEISPAMLVDAGVKYVVLGHSERREYFGETNEDVNKKVLKAFEHGIT-PIMCCGETLTQREQGVTMDFIRQQVKVGFQGVTA-
Clostridium_botulinum           KVGAQNMHFE-ESGAYTGEIAPKMLEELGVHYVIIGHSERRQYFNETDETVNKKVKKAFEHNLI-PIVCCGESLEEREGNITEKVLEGQIKVGLKELSK-
Clostridium_perfringens         KVGAQNMHFE-EKGAFTGEIAPRMLEAMNIDYVIIGHSERREYFNETDETCNKKVKAAFAHNLT-PILCCGETLEQRENGTTNDVIKAQITADLEGLTK-
Thermoanaerobacter_wiegelii     LLGAQDMFWE-EKGAYTGEISPLMLKEIGVKYVVIGHSERRQYFGETDEMVNKKVLSALSHGLS-PIVCVGESLSQREEGKTFEVVLNQTKEALKGVSH-
Brachyspira_murdochii           KLGAQNLYFE-EKGAFTGQISADMLLAVGCEYVIVGHSECRDIFGEKDELINKKVKRALDKNLI-PVLCVGEHLEEREKGITSDIVSSQTKEAFKGLSE-
Bartonella_henselae             LLGGQNCHFD-DYGPYTGDISAFMLKEAGASHVIIGHSERRTVYQESDAIVRAKVQAAWRAGLV-ALICVGETLEERKSNKVLDVLTRQLEGSLP---DG
Brucella_abortus                GLGGQDAHFK-TSGAHTGDISPEMLKEAGATHVILGHSERRTDHHESNKLICAKTEAAWAAGLV-AIVCVGETASERKAERALDVIGDQLSGSLP---DG
Rhizobium_etli                  SIGAQDCHQK-ASGAHTGDISAEMIADSFGTYVIVGHSERRTDHAETDHLVRAKAEAAFAAGLT-AIICIGETADERRAGQALDIIKRQLSASVP---DG
Agrobacterium_tumefaciens       LIGAQDCHQN-DTGAHTGDISAEMIADCFGTHVIVGHSERRTDHAETDHLVRAKASAAHQVDLI-AIVCIGETADERKAGQTLDILKRQLAGSLP---DE
Rhodobacter_sphaeroides         MVGGQDCHPK-TSGAHTGDVSAAMLADAGASHVILGHSERRADHGETDSLVRLKAEAAWGAGLV-AIVCVGETEAQRDAGQTLDVIGAQLAGSVP---DA
Ruegeria_sp                     AIGGQDCHAK-TSGAHTGDLSADMLRDAGATYVIIGHSERRADHGEGDADVRAKTEAAQAAGLV-AVVCIGETLEEREAGTTLEVVGAQLAGSLP---DG
Bradyrhizobium_japonicum        AVGAQDCHPK-ASGAHTGDIAAEMLANAGATAIIVGHSERRADHGEGDALVRQKAEAAWRAGVT-AIVCIGETQAQRDAGQTLDILRGQLDGSLP---DG
Rhodopseudomonas_palustris      AIGAQDCHAN-ATGAHTGDISAEMLADAGARAIIVGHSERRADHGETDAIVRTKAQAAWRAGLV-AIVCVGETQGERNAGHTLDVVAGQLAGSLP---DG
Methylobacterium_radiotolerans  AIGGQNLHAR-PSGAFTGSISAEMLADLGAKYVIVGHSERRAYHHETDDGVHAKALGARRAGLC-GIICVGETIEEREQGRALDIVRAQLAIGLP---KG
Rhodospirillum_rubrum           AVGGQDCHAK-AKGAHTGDVSAPMLADLGCQFVIVGHSERRADHGETDALVKAKAEAALAAGLI-AVICVGETEAQRDAGETLAVVSGQLAGSLP---TH
Zymomonas_mobilis               TFGAQDCHYD-ESGSHTGCVSAPMLREVGASYVIVGHSERRIEQNETNEEIRAKTTTAIANGLV-TIVCIGESRAAHASGQALPIIGAQLDGCIP---PD
Gluconacetobacter_hansenii      ALGAQDCHQD-PCGAHTGDIAASMLRDLGVQYVILGHSERRRDHHELDETVREKAVAAAAAGLI-PIICVGESADQRSNGDSQETVGWQIQGSLP---QG
Thermus_thermophilus            GYGAQDVSAH-KEGAYTGEVSARMLSDLGCRYAIVGHSERRRYHGETDALVAEKAKRLLEEGIT-PILCVGEPLEVREKGEAVPYTLRQLRGSLEG-VEP
Thermus_aquaticus               AYGAQDVSPH-REGAYTGEVSARMLADLGCRYAIVGHSERRRYHGESDALVAEKAKRLLEEGIT-PILCVGEPLEVRERGEAVPYTLAQLLGSLKG-VEP
Oceanithermus_profundus         AWGAQDVSAH-TFGAYTGEVAAEQLADLGCRYVVVGHSERRAYWNESDALVAAKARRAMEAGLV-PILCVGERLEEREAGRAVSFTLEQLAGSLEG-VGL
Deinococcus_deserti             AYGGQDVSQH-ESGAYTGEISAAMLRDVGAKYAIVGHSERREYHGETDAVVAAKARQAQAHGLT-PIVCVGEGLEVRERGEHVQYTLAQLRGSLEG-VG-
Bifidobacterium_bifidum         RYGAQAVSVT-TQGAFTGDVSADMIAHLGCSYVIVGHSERRKYHPEDDVNIVDQVRAVLAAGMQ-PILCVGESFEERRQGIELDFAVGQVHDVTRD-LSD
Bifidobacterium_longum          AYGAQSVSVT-TQGAFTGDVSADMIAHLGCSYVIVGHSERRKYHPEDDANIVDQVRAVLAAGMQ-PILCVGESFEERRQGIELDFAVGQVRDVTRD-LNE
Gardnerella_vaginalis           LYGAQAVSVT-AQGAFTGDVSADMLASLGCSMVIVGHSERRKYHPEDDANIVDQVRAVLAAGMQ-PILCVGESFEERKQGIELDFAVGQVHDVTRD-LDS
Nocardia_farcinica              TYGAQDVSVH-ESGAYTGEISASMLAKLGCTFVVVGHSERRQYHHEDDATVLGKAKKALEHGLT-PIVCIGEGLNVREAGTHVEYNLEQLRGSLKG-LSA
Mycobacterium_tuberculosis      TYGAQDLSPH-DSGAYTGDVSGAFLAKLGCSYVVVGHSERRTYHNEDDALVAAKAATALKHGLT-PIVCIGEHLDVREAGNHVAHNIEQLRGSLAG-LLA
Corynebacterium_glutamicum      TFGAQDVSQH-ESGAYTGEVSASMLAKLNCSWVVVGHSERREYHNESDELVAAKAKAALSNGIS-PIVCVGEPLEIREAGTHVEYVVEQTRKSLAG-LDA
Propionibacterium_acnes         AYGAQDISAH-DDGAYTGEVSGAMLSKLGCRYVVVGHSERREYHNESDELVNAKAKKVIENGMT-PIICCGEALEVRKAGKHVEHTVGQIKADLDG-IPA
Actinomyces_odontolyticus       KYGAQDVSIH-DNGAYTGEISTDMLTKLGVSYVVMGHSERREYHGESDELVGAKARKVFDAGMT-PILCCGEALEVRKAGTYVEFVLGQIRAALAG-WKP
Microbacterium_testaceum        ALGGQDLSAH-DSGAYTGEISGQFLAKLNARYVIIGHSERRQFHNETDEVVAAKTQAALRHGLV-PVICVGETSEDLEKFGASAVPVGQLEVALEG-VAS
Moritella_marina                ILGAQNTDLN-NSGAFTGDMSPAMLKEFGATHIIIGHSERREYHAESDEFVAKKFAFLKENGLT-PVLCIGESDAQNEAGETMAVCARQLDAVINT-QGV
Photobacterium_profundum        ILGAQNSDLN-NSGAFTGDMSPAMLKEFGASHIIIGHSERREYHNESDEFVAKKFAFLKENGLT-PVLCIGESDAQNEAGETMAVCARQLDAVINT-QGV
Vibrio_cholerae                 ILGAQNTDTH-NSGAYTGDMSPAMLKDFGASHIIIGHSERRDYHKESDEFVAKKFAFLKENGLT-PVFCIGETEAQNEAGETEAVCARQINAVIDA-YGV
Shigella_flexneri               MLGAQNVDLN-LSGAFTGETSAAMLKDIGAQYIIIGHSERRTYHKESDELIAKKFAVLKEQGLT-PVLCIGETEAENEAGKTEEVCARQIDAVLKT-QGA
Escherichia_coli                MLGAQNVDLN-LSGAFTGETSAAMLKDIGAQYIIIGHSERRTYHKESDELIAKKFAVLKEQGLT-PVLCIGETEAENEAGKTEEVCARQIDAVLKT-QGA
Salmonella_enterica             MLGAQNVDLN-LSGAFTGETSAEMLKDIGAQYIIIGHSERRTYHKESDELIAKKFAVLKEQGLT-PVLCIGETEAENEAGKTEEVCARQIDTVLKT-QGA
Klebsiella_pneumoniae           HLGAQNVDVN-LSGAFTGETSAEMLKDIGAQYIIIGHSERRTYHKESDELIAKKFAVLKEQGLT-PVLCIGETEAENEAGKTEEVCARQIDAVLKT-QGA
Enterobacter_aerogenes          HLGAQNVDVN-LSGAFTGETSAEMLKDIGAQYIIIGHSERRTYHKESDELIAKKFAVLKEQGLT-PVLCIGETEAENEAGKTEEVCARQIDAVLKT-QGA
Serratia_symbiotica             ALGAQNVDIN-LSGAFTGEISANMLKDVGAQYIIIGHSERRTYHKETDGAISEKFAVLKEAGLI-PVLCIGETEAENEAGKTEEVCARQIDAVLKT-LGA
Yersinia_pestis                 ALGAQNVDVN-LSGAFTGETSAEMLKDIGAQYIIIGHSERRTYHQESDELIAKKFGVLKEIGLI-PVLCIGESEAENEAGQTEAVCAKQLDAVLNT-LGV
```

```
Actinobacillus_pleuropneumonia  ALGAQNVDVN-VQGAFTGDISTEMLKDFGAKYIIIGHSERRTYHKECDTFIAKKFAALKAAGLV-PVLCIGETEAENETGQTEAVCAKQIDAVIDA-LGV
Pseudomonas_aeruginosa          DVGAQNSAVEPMQGALTGETAPSQLADVGCSMVLVGHSERRLILGESDEVVSRKFAAAQSCGLV-PVLCVGETRAEREAGKTLEVVARQLGSVIDE-LGV
Marinomonas_posidonica          VLAAQNLSQY-EKGAYTGEVSTAMLTDFKVTYVLVGHSERRSLYGETDEVVAEKVKMALSAGLK-PILCIGETLEEREAEQTLLVCERQVAAVIDV-VGI
Xylella_fastidiosa              AFGAQNVSHH-DKGAYTGEVSAAMVADVGAHYTLVGHSERREYHHEDSELVARKFAAALSAGLR-PILCVGESLPQREAGQAEVAIAMQLAPVLAL-VGP
Xanthomonas_campestris          AFGAQDVSSN-EKGAYTGEVSASMLVDVGAEYGLVGHSERRQYHQESSELVARKFAAAMHAGLI-PVLCVGESLEQREAGQTEAILRAQLEPVLSL-VGS
Marinobacter_algicola           AVGVQNVACW-SSGAYTGEISAAMAKDCGCGFTLVGHSERRQLFGETDEQVATKVEQALASGLV-AVICVGETLEERDAGKAEDVVSRQVRKAVAD-VGN
Aeromonas_caviae                KLGAQNVSQH-RQGAYTGEISCEMLVEAGCRYVLVGHSERRRLFGETSFIVAEKFAAVRAAGLI-PILCLGESGAARQARRTFEVIAEELDIVIEK-NGA
Pseudoalteromonas_haloplanktis  STGTQTVSEN-TPGAFTGEVDAQLIKELGAQYVLVGHSERRSIYKESNDVVAAKFARAQQVGLT-PILCVGESESEQENGKTEQIVAAQIDAVIEK-LGV
Legionella_pneumophila          VVGAQNVYPK-DYGAYTGELSAPMLKDFDCRYVLVGHSERRQFFHEDENFVAQKFHHVKDHGMI-PILCVGETLSERENGKTEQIIAQQVLAVSAK--GK
Ralstonia_solanacearum          AWGAQDVSAE-TRGAFTGEVAASMLSEFGCGYVIVGHSERRTYHAETDAQVATKALRALEHGIT-PIVCVGETLVQREAGETEQVVARQLDAVLES-LSL
Burkholderia_sp                 VWGVQDVSAF-THGAYTGEVAAPMVTDFGATFAIVGHSERRAYHRESAELVAVKAQRVLDAGLT-PIVCVGETLEEREAGSTEQVVGGQLDEVLAK-LSA
Bordetella_pertussis            GWGAQDVSAH-AKGAYTGEVAAPMLAEFGCRWVLVGHSERRTLHAESDQLVADKARAALEAGLT-PVVCVGESLQEREGGNTLGVIERQLEPVLA--LGR
Nitrosococcus_watsonii          NWGAQNLSHH-EMGAYTGEIAPSMLADLGCRFVIIGHSERRTLYGETDSLVAEKAIAAQKVDII-PIICVGETLQEREQNITEQVVERQLNAVLQL-AGV
Coxiella_burnetii               SWGAQDVSEF-ERGAYTGEVSAAMLRDFHCRYVIVGHSERRQRHGETNEQVAAKVRAALRCGIR-PIICVGETEKQRNANQTLSVIKEQLAVVLQMNDNL
Variovorax_paradoxus            ALGAQDVSAH-PQGAFTGEQSAAMLKDFGVRYAIVGHSERRQYHGETDETVAAKAAAALANGIT-PIVCVGETLAEREAGQTEEVVKRQLAAVIHV--NG
Halomonas_elongata              ALGAQTLSDQ-PSGAFTGEVSGEMLREVGTSLVLVGHSERRTQFGEDDSAVLARVRRALECELT-PVLCLGETLEERDADRGEAVVIGQLEAVLDG-LEA
Sphaerobacter_thermophilus      VLGAQDCAVE-DQGAFTGEVSARMLAPL-CRYIIVGHSERRHIIGETDEVVAAKLRAVLRNGAR-PILCVGELLEERERGEARAVVERQLQSAFAD-VEA
Anaerolinea_thermophila         GMGAQNMHWE-ASGAFTGEVSPKMVAEF-CQYVILGHSERRTYFGETDETVNRKVKSALSTGLT-PIVCVGETLAENEAGKTAEVVWRQITEGLKD-LLP
Myxococcus_xanthus              ALAAQNCHWE-SSGAFTGEISAPMLAELGCAYVIVGHSERRQLFGDTDEQVNKRAKAVRAAGMT-PIICVGETLAEREANQTLAVVERQVRGALEG-FGA
Roseiflexus_castenholzii        ALGAQDVYIE-PQGAFTGAISPVMLRDLGCAYVIVGHSERRAIFGEGDELIGKKVRAALAHDLT-PILCVGETKPQRDAGHAETVVVAQVRAALTG-MTP
Chloroflexus_aurantiacus        GLGAQNLYPE-AQGAFTGEVSPPMLVDIGCRYVIIGHSERRQYFGESDAFVNRKLRAALAHGLR-PIVCVGESKPQRDAGQAEPIVTAQVRAALLE-VPP
Collinsella_tanakaei            KLGAQNVYWE-AAGAYTGESSCDMLKSAGCTYCIIGHSERRDYFHETDEDQNKKAKALVAAGLV-PVFCCGEPLEVREAGTYVEHVVAQVKAGLEG-LEI
Atopobium_vaginae               ALGAQNVYWE-ENGAFTGETAPNMLVSLGATYCIIGHSERRGYFGETDEDINKKAKALLAHNIV-PISCCGESLEVREAGKHVEFVVEQIKADTKG-LTL
Eggerthella_sp                  AIGAQDVYWE-PKGAFTGEISVPMIKEIGCTYCIVGHSERRELFGETNEDVNRKAKALIAGGLA-PIVCVGESLAVRDEGTTNEYVCAQVRAAFAG-IDA
Ostreococcus_lucimarinus        AVGAQNIYFE-ASGAFTGEMSAEMAKSVGCTHVLIGHSERRELFGETDEMTRLKTRKTLDCGMT-AVLCIGETKEEYERGEAKKVCNRQLSAALSG-VEA
Lentisphaera_araneosa           GIGAQNVHFA-ESGAYTGELSAAMLKEAGIELVIIGHSERRQYFGETNETVNLRTKAALAAGIK-PFVCIGETLEEREGGNMESVLKAQIVEGFAD-ITA
Acholeplasma_laidlawii          RIGAQNMHYA-DEGAFTGENSPAQVKTAGAEYILIGHSERRSLFNETDKDVNLKMHAAFKYDLA-PILCIGEQLDTREANKTKEVLDVQLDKAFEG-VSK
Lactobacillus_reuteri           KIIAQNAAAE-YSGAFTGEISLRGLADAGVSYVMLGHIERRHLFHEDNELVNRKVLAALQMGVT-PIICTDETMVQKEVNGEIHYVFQQLMSVLRG-VSL
Bacillus_subtilis               KVGAQNMHFE-ESGAFTGEISPVALKDLGVDYCVIGHSERREMFAETDETVNKKAHAAFKHGIV-PIICVGETLEEREAGKTNDLVADQVKKGLAG-LSE
Bacillus_thuringiensis          QVGAQNMHFE-KNGAFTGEISPVALSDLKVGYVVLGHSERREMFAETDESVNKKTIAAFEHGLT-PIVCCGETLEERESGKTFDLVAGQVTKALAG-LTE
Listeria_monocytogenes          RVSAQNCYFE-DEGAFTGEISPFALADLGVSYVIIGHSERREYFHETDEDINKKAHAIFKHGMT-PIICCGETLDQREAGQTDTWVRGQIRAALAG-LTE
Geobacillus_stearothermophilus  KIGAQTMHFA-DQGAYTGEVSPVMLKDLGVTYVILGHSERRQMFAETDETVNKKVLAAFTRGLI-PIICCGESLEEREAGQTNAVVASQVEKALAG-LTP
Staphylococcus_aureus           EIGAQNTYFE-DNGAFTGETSPVALADLGVKYVVIGHSERRELFHETDEEINKKAHAIFKHGMT-PIICVGETDEERESGKANDVVGEQVKKAVAG-LSE
Enterococcus_faecalis           KLAAQNCYWE-NAGAFTGENSPAAIADLGVDYVIIGHSERREYFHETDEDINKKAKAIFANGMT-PIFCCGETLETYEAGKTAEWIEGQITKGLVG-LSN
Lactococcus_lactis              KLAAENSYFE-NAGAFTGENSPAAIVDLGIEYIIIGHSERREYFHETDEDINKKAKAIFAAGAT-PILCCGETLETFEAGKTAEWVSGQIEAGLAG-LTA
Streptococcus_pneumoniae        KVAAQNCYFE-NAGAFTGETSPQVLKEIGTDYVVIGHSERRDYFHETDEDINKKAKAIFANGML-PIICCGESLETYEAGKAAEFVGAQVSAALAG-LTA
Erysipelotrichaceae_bacterium   VIAAENCHWE-DSGAFTGEISVPMLQEVGVTHCIIGHSERREMFNDTDETVNKKAKRLIDAGIT-PILCIGETEAQYDAGDSEKVIRDQLTGSLAD-MCP
Tenebrio_Molitor                GVAAQNCYKV-PKGAFTGEISPAMIKDVGADWVILGHSERRQIFGESDELIAEKVCHALES-GLKVIACIGETLEEREAGKTEEVVFRQTKAIAAKVND-
Bombyx_mori                     EVAAQNCWKS-PKGAFTGEISPAMIKDVGVNWVILGHSERRTIFGEKDELVAEKVAHALES-GLKVIACIGETLEERESGKTEEVVFRQLKALVSAIGDK
Apis_mellifera                  SIAGQNTYKV-AKGAFTGEISPAMLLDNGIPWVILGHSERRNIFGENDELIAEKVAHALES-GLKVIACIGEKLEEREAGKTDEVVFRQTKAIANKINS-
Aedes_aegypti                   GVAAQNCYKV-AKGAFTGEISPAMLKDLNIGWVILGHSERRAIFGESDELVADKVAHALAE-GLKVIACIGETLQEREAGQTEAVCFRQTKAIADKVKD-
Anopheles_gambiae               GVAAQNCYKV-AKGAFTGEISPAMLKDLGLGWVILGHSERRAIFGETDELIAEKVAHALAE-GLKVIACIGETLQEREAGQTEAVCFRQTKAIAAQVKD-
Drosophila_melanogaster         GLAGQNAYKV-AKGAFTGEISPAMLKDIGADWVILGHSERRAIFGESDALIAEKAEHALAE-GLKVIACIGETLEEREAGKTNEVVARQMCAYAQKIKD-
Rhipicephalus_microplus         ALAAQNCYKV-EQGAFTGEISPGMIKDCGGQWVILGHSERRHVFKEDDVLIGEKIKHALES-GLNVIACIGELLEDREAGRTEEVCFRQIKHIASNVKD-
Ixodes_scapularis               AVSAQNCYKV-EKGAFTGEISPAMIKDCGATWVILGHSERRNVFKESDELIGDKVHHALES-GLNVIACIGELLEEREAGKTEEVVYRQTAAIAAKVTD-
Anolis_carolinensis             GVAAQNCYKV-AKGAFTGEISPAMIKDIGATWVILGHSERRHIFGESDELIGQKVAHALAE-GLGVIACIGEKLDEREAGITEKVVFEQTKAIADNVKD-
Gallus_gallus                   GVAAQNCYKV-PKGAFTGEISPAMIKDIGAAWVILGHSERRHVFGESDELIGQKVAHALAE-GLGVIACIGEKLDEREAGITEKVVFEQTKAIADNVKD-
Canis_lupusfamiliaris           AVAAQNCYKV-TNGAFTGEISPGMIKDCGATWVVLGHSERRHVFGESDELIGQKVAHALAE-GLGVIACIGEKLDEREAGITEKVVFEQTKVIADNVKD-
Oryctolagus_cuniculus           AVAAQNCYKV-TNGAFTGEISPGMIKDCGATWVVLGHSERRHVFGESDELIGQKVAHALSE-GLGVIACIGEKLDEREAGITEKVVFEQTKVIADNVKD-
Pan_troglodytes                 AVAAQNCYKV-TNGAFTGEISPGMIKDCGATWVVLGHSERRHVFGESDELIGQKVAHALAE-GLGVIACIGEKLDEREAGITEKVVFEQTKVIADNVKD-
Homo_sapiens                    AVAAQNCYKV-TNGAFTGEISPGMIKDCGATWVVLGHSERRHVFGESDELIGQKVAHALAE-GLGVIACIGEKLDEREAGITEKVVFEQTKVIADNVKD-
Nomascus_leucogenys             AVAAQNCYKV-TNGAFTGEISPGMIKDCGATWVVLGHSERRHVFGESDELIGQKVAHALAE-GLGVIACIGEKLDEREAGITEKVVFEQTKVIADNVKD-
Pongo_abelii                    AVAAQNCYKV-TNGAFTGEISPGMIKDYGATWVVLGHSERRHVFGESDELIGQKVAHALAE-GLGVIACIGEKLDEREAGITEKVVFEQTKVIADNVKD-
Macaca_mulatta                  AVAAQNCYKV-TNGAFTGEISPGMIKDCGATWVVLGHSERRHVFGESDELIGQKVAHALAE-GLGVIACIGEKLDEREAGITEKVVFEQTKVIADNVKD-
Bos_taurus                      AVAAQNCYKV-ANGAFTGEISPGMIKDLGATWVVLGHSERRHVFGESDELIGQKVAHALAE-GLGVIACIGEKLDEREAGITEKVVFEQTKVIADNVKD-
Sus_scrofa                      AVAAQNCYKV-ANGAFTGEIGPGMIKDLGATWVVLGHSERRHVFGESDELIGQKVAHALAE-GLGVIACIGEKLDEREAGITEKVVFEQTKVIADNVKD-
Mus_musculus                    AVAAQNCYKV-TNGPFTGEISPGMIKDLGATWVVLGHSERRHVFGESDELIGQKVSHALAE-GLGVIACIGEKLDEREAGITEKVVFEQTKVIADNVKD-
```

Rattus_norvegicus	AVAAQNCYKV-TNGAFTGEISPGMIKDLGATWVVLGHSERRHIFGESDELIGQKVNHALSE-GLGVIACIGEKLDEREAGITEKVVFEQTKAIADNVKD-
Xenopus_laevis	ALSAQNCYKV-AKGAFTGEISPAMIKDCGATWVILGHSERRHVFGECDELIGQKVAHALSE-GIGVIACIGEKLDQREAGITEKVVFEQTKAIADNVKD-
Danio_rerio	DVAAQNCYKV-AKGAFTGEISPAMIKDCGVKWVILGHSERRHVFGESDELIGQKVAHALEN-GLGVIACIGEKLDEREAGITEKVVFAQTKFIADNVKD-
Ictalurus_punctatus	GVAAQNCYKV-AKGAFTGEISPAMIKDCGVEWVILGHSERRHVFGESDELIGQKVAHALEN-GLGVIACIGEKLDEREAGITEKVVFAQTKAIADNVKD-
Oreochromis_niloticus	GVAAQNCYKV-PKGAFTGEISPAMIKDCGVNWVILGHSERRHVFGESDELIGQKTAHALEN-GLGVIACIGEKLDEREGGITEKVVFAQTKVIADNVKD-
Nematostella_vectensis	GVAAQNCYKV-ASGAFTGEISPAMIKDIGCEWVIIGHSERRNIFGEKDELIGEKVAHALSA-NVKVIACIGELLSEREAGKTQEVVFRQIKAIADKIS--
Schistosoma_mansoni	HVAAQNCYKV-SKGAFTGEISPAMIKDIGCDWVILGHSERRNIFGESDELIAEKVQHALAE-GLSVIACIGETLSERESNKTEEVCVRQLKAIANKIKSA
Strongylocentrotus_purpuratus	FVAAQNCYKV-PKGAFTAETSPAMLQDLGVGWVILGHSERRHVFGESDELIAQKVAHALSC-KLKVVACIGEKLDEREKGQTNEVVYRQMRALADVITD-
Caenorhabditis_elegans	LVAAQNCYKV-PKGAFTGEISPAMIKDLGLEWVILGHSERRHVFGESDALIAEKTVHALEA-GIKVVFCIGEKLEEREAGHTKDVNFRQLQAIVDKGVS-
Brugia_malayi	EVSAQNCYKV-EKGAFTGEISPAMIKDLGLHWVILGHSERRHIFGESDELIAEKVLHAVDS-GLQTIFCCGEKLDEREAGKTKAVNFRQLQAVIDKKAN-
Ricinus_communis	HVAAQNCWVK-KGGAFTGEVSAEMLVNLSIPWVILGHSERRLLLSESNEFVGDKVAYALSQ-GLKVIACVGETLEQREAGSTMDVVAAQTKAIAERVKD-
Vitis_vinifera	HVAAQNCWVK-KGGAFTGEISAEMLVNLGIPWVIIGHSERRLLLNESNEFVGEKVAYALSK-GLKVIACVGETLEQRESGSTMEVVAAQTKAIADKVSN-
Arabidopsis_thaliana	FVAAQNCWVK-KGGAFTGEVSAEMLVNLDIPWVILGHSERRAILNESSEFVGDKVAYALAQ-GLKVIACVGETLEEREAGSTMDVVAAQTKAIADRVTN-
Zea_mays	HVAAQNCWVK-KGGAFTGEVSAEMLVNLGVPWVILGHSERRALLGESNEFVGDKVAYALSQ-GLKVIACVGETLEQREAGSTMDVVAAQTKAIAEKIKD-
Oryza_sativa	QVAAQNCWVK-KGGAFTGEVSAEMLVNLSIPWVILGHSERRSLLGESNEFVGDKVAYALSQ-GLKVIACVGETLEQRESGSTMDVVAAQTKAISERIKD-
Glycine_max	HVSAQNCWVR-KGGAYTGEVSAEMLVNLGIPWVIIGHSERRQLLNELNEFVGDKVAYALQQ-GLKVIACIGETLEQREAGTTTAVVAEQTKAIAAKISN-
Perkinsus_marinus	QISAQNCSKT-GPGAYTGEITAEMIKDAGLNWTILGHSERRHLFNETNEDLAAKVAKAEAA-GLKIIFCIGELLEEREAGKTEEVCATQMDAIVPVVKD-
Chlamydomonas_reinhardtii	QLSAQNAWIG-GNGAFTGEVSAEQLTDFGVPWVILGHSERRSLFGESNEVVAKKTSHALAA-GLGVIACIGETLEQRNSGSVFKVLDAQMDALVDEVKD-
Trypanosoma_brucei	VIAAQNAIAK-S-GAFTGEVSLPILKDFGVNWIVLGHSERRAYYGETNEIVADKVAAAVAA-GFMVIACIGETLQERESGRTAVVVLTQIAAIAKKLKK-
Trypanosoma_cruzi	QIAAQNAITR-S-GAFTGEVSLQILKDYGIKWVVLGHSERRLYYGETNEIVAEKVAQACAA-GFHVIVCVGETNEEREAGRTAAVVLTQLAAVAQKLSK-
Leishmania_mexicana	VISAENAIAK-S-GAFTGEVSMPILKDIGVHWVILGHSERRTYYGETDEIVAQKVSEACKQ-GFMVIACIGETLQQREANQTAKVVLSQTSAIAAKLTK-
Entamoeba_histolytica	LVSAENAWTK-S-GAYTGEVHVGMLVDCQVPYVILGHSERRQIFHESNEQVAEKVKVAIDA-GLKVIACIGETEAQRIANQTEEVVAAQLKAINNAISK-
Plasmodium_falciparum	STGIQNVSKF-GNGSYTGEVSAEIAKDLNIEYVIIGHFERRKYFHETDEDVREKLQASLKN-NLKAVVCFGESLEQREQNKTIEVITKQVKAFVDLIDN-
Toxoplasma_gondii	HVGLQNCSKT-HNGAFTGEISVEMIKDFGLRWILAGHSERRQYYGESDEVVAEKVNIILQEKDLNVVLCVGEQLKDREANKTNDVVDAQLAACLPKISD-
Cryptosporidium_parvum	KIGSQNISCT-GNGAFTGEVSCEMLKDMDVDCSLVGHSERRQYYSETDQIVNNKVKKGLEN-GLKIVLCIGESLSERETGKTNDVIQKQLTEALKDVSD-
Paramecium_tetraurelia	QVALQNSSLT-KVGAYTGEISVEQVKDLAIPWVILGHSERRQYYGETNEIVGKKTRIALDY-QLSVMACVGEKLAEREAGQTTQVIQAQLDAIKKELTTA
Aspergillus_niger	GVAAQNVFDK-PNGAFTGEISVEQLRDVKIDWTIIGHSERRVILKESDEFIARKVKAAIDG-GLSVIFCIGETLEEREANKTIDVVTKQLNAVAKELTKE
Paracoccidioides_brasiliensis	AVSAQNVFDK-PNGAFTGELSVEQLRDEKITWTLAGHSERRVLLREDDEFVARKTKAAING-GLNVILCIGESLEEREAGKTIDVVTRQLDAVAEEVSPA
Schizosaccharomyces_pombe	GVGAQNVFDK-KNGAYTGENSAQSLIDAGITYTLTGHSERRTIFKESDEFVADKTKFALEQ-GLTVVACIGETLAEREANETINVVVRQLNAIADKVQ--
Saccharomyces_cerevisiae	TVGAQNAYLK-ASGAFTGENSVDQIKDVGAKWVILGHSERRSYFHEDDKFIADKTKFALGQ-GVGVILCIGETLEEKKAGKTLDVVERQLNAVLEEVK--
Kluyveromyces_lactis	KISAQNAYSK-ASGAYTGENSVEQIKDVGAEWVILGHSERRTYFNETDEIVAEKTKFALDS-GVKVILCIGETLEEKQQNITLQVVQRQLQAVLEKVQ--
Candida_dubliniensis	AVGAQNVFDK-ACGAFTGETCASQILDVGATWTLTGHSERRTIIKESDEFIAEKTKFALDN-GVKVILCIGETLEERKGGITLDVCARQLDAVSKIVS--
Giardia_lamblia	RIAAQNVYLE-GNGAWTGETSVEMLQDMGLKHVIVGHSERRRIMGETDEQSAKKAKRALEK-GMTVIFCVGETLDERKANRTMEVNIAQLEALGKELGES
Trichomonas_vaginalis	KVSAENVFTK-PNGAFTGEVTVPMIKSFGIEWTILGHSERRDILKEDDEFLAAKAKFALEN-GMKIIYCCGEHLSEREAGKASEFVSAQIEKMIPAIPAG
Sphingobacterium_sp	SVGAQNIHQE-DAGAYTGEISAKQVRSTGAEYVILGHSERRAYFGESDELLAQKVNIALTH-GLIPIFCIGETREERESGDFFDVIKTQLEKGVFHLSAS
Pedobacter_saltans	FVGAQNAHQE-ASGAYTGEISTGMLKSVGVEYVILGHSERRQYFGENNELLAKKTNTALAN-DLKPIFCIGETLAERESNQHFDVIKTQLAEATFHLSAE
Marivirga_tractuosa	VLAGQNCHQE-DAGAFTGEVSAPMLKSLGAQYVILGHSERREHFSETDEELAKKTDNALKN-DLKVIFCCGEPLEIREADTQNEYVTKQLTNSLFHLTPE
Cyclobacterium_marinum	ALGAQNCSDK-EAGAYTGETSAAMLASFGAEYVIIGHSERRSMFNESNELLTEKTKQALSN-GLTPIFCCGEPLEIREADTHEDYVKAQLTESLFGFSEE
Leadbetterella_byssophila	ALAAQNCYPK-ASGAYTGEVSVPMLKSVGVKYVIIGHSERREYFAESDAFIAEKVDAILAE-GLTPIFCCGETLSQREAGIHFDFVKGQLTNSLFHLSEE
Haliscomenobacter_hydrossis	YLGAQNCHQE-DKGAYTGEISVDMLKSVGVSYVILGHSERREYFGESDELLAKKTDKVLAA-GLLPIFCCGESLDIRDAGTHVAHVQAQIKAGLFHLSPE
Parabacteroides_distasonis	GVAAENCADK-EKGAYTGEVSAAMVASTGAKYVILGHSERRAYYHETPEILKTKVELALAN-GLTPIFCIGEVLEEREAGKHFEVVDAQIAGSLFDLSAE
Bacteroides_fragilis	GVGAENCADK-ESGAYTGEVSAAMVASTGAKYVILGHSERRAYYGETVEILKDKVKLALAN-GLTPIFCIGEVLEEREANKQNEVVAAQLA-SVFDLSAE
Prevotella_ruminicola	GLGAENCADK-EKGAFTGEVSAEMVKSTGAQYVILGHSERREYYKETPEILKEKVLLAQKN-DLKVIFCIGESLEEREAGKQNEVVKAELEGSVFNLSEE
Alistipes_shahii	ALGAQDCATE-AKGAYTGEIAASMIAALGCKYVILGHSERRQYYGETSATLNKKMAQAYAN-GLIPIYCVGENLDEREAGKHFDVVKAQIEEVVYNLTEE
Porphyromonas_asaccharolytica	EYAAQDCSAH-DAGAYTGEVSAEMIRSCGCTYVIIGHSERRAYHKESAELLAQKIDQALAH-GLKVIFCVGEQQAEREGNRYFEVVDEQLRGSLGHLSHE
Cellulophaga_lytica	QVIAQNMHFA-DNGAYTGEISADMLLDIGINTVIIGHSERRAYFGETDEILAKKVIKALEK-NLRVVFCFGEELEDRKSDNHFNIVASQLKNALFNLKPE
Kordia_algicida	EVIAQNMHQE-AKGAYTGEVSARMLKSVGIKTVILGHSERRAYFGETDELLAKKVDAALAN-ELEIIFCFGEELEDRKAGNHFNIVENQLKNALFHLDTA
Flavobacterium_psychrophilum	RVAAQNMHQA-ENGAYTGEISADMLKSIGVNTVILGHSERRAIFHETDAIISFKVDTALRH-EMTVIFCFGEELKDRQNKQHFNIVENQLRDGLFHIDKS
Neisseria_gonorrhoeae	LTCAQDVSRFPDNGAYTGEVSAEMLADTGTDIVLIGHSERSLYFGEKNEIQRRKMENVLNVGLI-PLLCVGESLEERETGKEHEVIAHQLS-ILQGLDTK
Francisella_tularensis	-VGLQNIT-FYDDGAYTGEISARMLEDIGCDYLLIGHSERRSLFAESDEDVFKKLNKIIDTTIT-PVVCIGESLDDRQSGKLKQVLATQLSLILENLSVE
Mycoplasma_genitalium	LLFAQDANFI-ESGSYTGTVSFTQLQDIGVNNSIIGHSERRKYYNETSAVINQKLFACLKASMQ-VVLCIGEAL-----GQEISFLKTDLTNCLDTIDKS
Ureaplasma_urealyticum	KLYAQNPSAY-SKGPYTGQISCLQLLDSNIKNTLVGHSEIRIDCSQS--IIDQKTKICMDL-LDQVIICIGEPLDVYEQK--KSLSFVLSQLANVINYKG
Ureaplasma_parvum	NLYAQNPSLH-NKGPYTGQISCLQLLDINIKNALVGHSEIRIDFSQS--IIDQKIKISMDL-LEQVIICVGETFDAYKQN--KSLNFVLNQLANIINYKG
Buchnera_aphidicola	FLGSQNVDIN-LNGAFTGETSILMLRDIGVKYVIIGHSERRFLHHETDDIIAKKFHLIKKS-NLTPILCVGETEIEKKHN--QTEQVIQRQLNLILKNLG
Campylobacter_jejuni	IQGAQNFYPC-VNGAFTGELGKEHLDEFGIKCVLIGHSERRALGDEE--FIKAKFDFAKEH-GYKIVFCIGENLDTKNSG--KTLEFLKKQLEIIDLNYE
Helicobacter_pylori	TLGVQNAYPR-DCGAFTGEITSKHLEELKIHTLLIGHSERRTLLKESPSFLKEKFDFFKDK-NFKIVYCIGEELTTREKGFKAVKEFLSEQLENIDLNYS
Orientia_tsutsugamushi	SAQDVSMITE-SSGPYTGEISASMLKNLNVNYAIVGHSERRLLFYEDANTIALKVRNCINN-AIVPIVCIGEPIEARKNK--TYLQYLAQQLSSISFSFT

```
Wolbachia_sp                    NIGAQNCHHK-KFGSYTGEISAEMLKELGCTYVILGHSER---ANEKDSEIKLKSEIAIES-GLHPIICVGENSEDYKNE--KTKEVIEYQCKNR-LPTH
Methanocaldococcus_Jannaschii   -VYAQHIDNI-NPGSHTGHILAEAIKDCGCKGTLINHSEKRMLLADIEAVINKCKNLGLET-----IVCTNNINTSKAVAALSPDC--------------
Methanococcus_voltae            -VYSQHMDAI-KPGSNTGKILPEAVKSTGAFGTLINHSEKRLLLSDIEEIINKSKELKLES-----VVCTNNIGVSKAVSALSPDY--------------
Methanothermococcus_okinawensi  -VYAQHFDAV-SPGSHTGHVLAETLKDCGLNGSLLNHSEKRMILADLEKSIEIAKNYNLET-----IVCTNNIGVSKAVAALNPNM--------------
Pyrococcus_Woesei               -VFAQHIDPI-KPGSHTGHVLPEAVKEAGAVGTLLNHSENRMILADLEAAIRRAEEVGLMT-----MVCSNNPAVSAAVAALNPDY--------------
Pyrococcus_furiosus             -VFAQHIDPI-KPGSHTGHVLPEAVKEAGAVGTLLNHSENRMILADLEAAIRRAEEVGLMT-----MVCSNNPAVSAAVAALNPDY--------------
Thermofilum_pendens             -VFSQHVDDV-PPGSYTGHVTLEAVKDAGAVGTMVNHSERRVRADQVDVIVKRARSLGLTT-----VVCTNTPEVTAAMAALGPDM--------------
Methanothermobacter_thermautot  -VLAQHIDAV-DAGGHTGSILAECARDAGAAGTLINHSEKRMQLADIEWVISRMKELEMMS-----VVCTNNVMTTAAAAALGPDF--------------
Methanosaeta_thermophila        -VYAQHVDGV-GFGSFTGHITAASVKAAGASGSLINHSERRLRLADIEASLRACRSEGLTS-----IICTNNVATTRAAAALRPDY--------------
Natronobacterium_gregoryi       -TWAQHVDPI-EHGSNTGHTLAESVADAGAVGTLVNHSERRLKLADIDGAVRAAERADLET-----VVCANNPEQIGAGAALGPDA--------------
Halobacterium_salinarum         -TYAQHVSPT-EHGSHTGSVLAESVADNGAVGTLLNHSEHRRRLADIDGSVAAAERAGLDT-----VVCANNPAQVAAAAALGPDA--------------
Halogeometricum_borinquense     -TWAQHVDPN-GYGSHTGSTLAEAVAEAGADGTLINHSEKRLKLADIDGSVQAAARAGLET-----CVCANNPAQIGAVAALGPDS--------------
Methanosphaerula_palustris      -VFAQHLDGV-VPGAFTGHVTAAAVRQTGAVGTLINHSERRLTLAEIEASVRAATEAGLVT-----VICSNNDMTSAAAAALSPTY--------------
Thermoproteus_tenax             -VYAQGADVE-AGGAHTAHVSLENIKEAGGSGVILNHSEAPLKLNDLARLVAKAKSLGLDV-----VVCAPDPRTSLAAAALGPHA--------------
Pyrobaculum_islandicum          -VYAQGADVE-TPGAYTAHIAVDNIKAVGASGLILNHSEAPLALNQLSKLAARAKSIGLDV-----VICAPDPVTSLAAAALGPHA--------------
Cenarchaeum_symbiosum           -VLAQHIDDK-GVGSTTGYVVPELLGESGVSGALINHSEHRVSADQVASLVPRLRGLDMIS-----VVCVKDSAEAANLSRHRPDY--------------
Nitrosopumilus_maritimus        -IFAQHIDDS-KVGSTTGFVIPELLKKSQVNGSLINHSEHRISSKEITKLVSKLKELKMTS-----IVCVKDVAEARKYAKLNPNY--------------
Desulfurococcus_kamchatkensis   KVFAQHADPV-EPGAITGFIPLEGLKNLGAHGVILNHSEHRLKISDINYLVKKARRLGIET-----LVCADTPETGAAVAVLGPDM--------------
Pyrolobus_fumarii               IVYAQHVDPV-EPGAHTGSVPVEAVKEAGARGSLVNHSERKLRLDEIERVVERLKVVGLEQ-----LVCADTPRAAAAIAVFEPTY--------------
Acidilobus_saccharovorans       -VYIEHADPV-DYGAYTGYLPAKAARLLGVRGILVNHSEHKMIYRDVAKVVETASKDGIET-----MACADTPGEAAGLAYLRPTY--------------
Sulfolobus_islandicus           -IYAEHVDPV-PLGAYTGAVLPEMIKDADAKGTLINHSERRLRADEIDDVLKRTRKLGLSS-----ILCVDRYELVYPFSLLRPDA--------------
Clustal Consensus                            .  *.                 .* *      :                      *

                                       210       220       230       240       250       260       270       280       290       300
                                ....|....|....|....|....|....|....|....|....|....|....|....|....|....|....|....|....|....|....|....|
Dictyoglomus_thermophilum       -ENVEKIVIAYEPVWAIGTGHSAKGEDANEVAG-LIRKIISEMYD--TEVSQKIRIQYGGSVNPQNITEFLSQNEIDGALVGGASLKPQSFWNIVKS---
Aquifex_aeolicus                -EHTDKIDIAYEPVWAIGTGTPATPEDAVEVHT-FIRNLINQLNP--KNE-GKTRILYGGSVNPQNAKEFMKHEEINGLLVGTASLDPESFAKIVYSF--
Thermodesulfovibrio_yellowston  --SPEALTIAYEPVWAIGTGVVATEEQIKQSHL-FIRNQLKEIYG--ERA-NEVRILYGGSVTPENIKSIMAIDNVEGVLVGGASLDPLKFAKIVKY---
Dehalogenimonas_lykanthroporep  ---SPDIYVAYEPVWAIGTGRAATGDIANETAM-LIRNIVGKLYG--NAVSQALPVLYGGSVNPENISDFISRSDIDGALVGGASIKAGQFVEIVRKS--
Dehalococcoides_sp              ---LSCIIIAYEPIWAIGTGKAATASEANSAIG-YIRRVLGDTLG--NAAAQTSPILYGGSVNEKNITEILSQTNIDGALVGGASLKAESFVSICRQAAV
Nostoc_punctiforme              ---QNNLVIAYEPIWAIGTGETCEAVEANRIIG-LIRSQLSNP---------NVSIQYGGSVKPNNIDEIMAQPEIDGVLVGGASLEPESFARIVNFHLV
Anabaena_variabilis             ---QTNLVIAYEPIWAIGTGDTCETTEANRVIG-LIRSQLKNP---------DVPIQYGGSVKPNNIDEIMAQPEIDGVLVGGASLEAASFARIVNYQ--
Trichodesmium_erythraeum        ---QENLVIAYEPIWAIGTGDTCEAKEANRVIG-LIRGKLNNS---------NVTIQYGGSVKPNNVDDIMAQEEIDGALVGGASLNPESFSRLVNYQ--
Crocosphaera_watsonii           ---QSNLVIAYEPIWAIGTGDTCESTEANRIIR-VIRDQLSNK---------NVTIQYGGSVKPNNIDEIMAQSDIDGALVGGASLDPVNFARIINYQ--
Synechocystis_sp                ---QSNLVIAYEPIWAIGTGDTCAATEANRVIG-LIREQLTNS---------QVTIQYGGSVNANNVDEIMAQPEIDGALVGGASLEPQSFARIVNFQP-
Synechococcus_sp                ---QFNLVIAYEPIWAIGTGDTCAADEADAVIG-KIRALLDNK---------EVSIQYGGSVKPENVDEIMAQSEIDGALVGGASLTPDSFARLANYQDQ
Cyanobacterium_UCYN             ---QGNLVIAYEPIWAIGTGETCNVLEANRIIG-IIREQVTNK---------GITIQYGGSVTPKNISEIMTQPNIDGVLVGGASLNPLSFSEIINY---
Prochlorococcus_marinus         ---KKKLIVAYEPIWAIGTGKTCEANEANRICG-LIRQWANCP---------EILIQYGGSVKPGNIDEIMAMSDIDGVLVGGASLDPESFARISNYKVE
Chlamydophila_pneumoniae        ---GSEFLIAYEPVWAIGTGKVAEASDVQDIHM-FCREVVAERFS--EATAEEISILYGGSVKVDNAQRFGQCSDVDGLLVGGASLEGQSFFEVAKNFNV
Desulfovibrio_vulgaris          -YVPQSLVVAYEPVWAIGTGKVAGPAEVVEAHA-LVRSLLEARYG--RD-GAAIRILYGGSVKPDNAAELLSLDNVDGLLVGGASLQAVSFSRIILA---
Mesorhizobium_opportunistum     -VKGATILFAYEPVWAIGDKGIPASSDYADKQ----QGLIKAVAG--SLLPSVPSVLYGGSVNPGNAAELIGQPNVDGLFIGRSAWQAQGYIDILGRASA
Geobacter_metallireducens       --DLAKIVIAYEPVWAIGTGKTATDEQAQIAHA-FIRKVVGELFT--VQIAETIRILYGGSVKPENVRGLMNQPDIDGALVGGASLKADSFGAIVNYKA-
Desulfobacter_postgatei         --DLDTLILAYEPVWAIGTGKTAGPEQVKEVHG-FLRHLLKEKYA--EGLAATIRILYGGSVKPGNIKDLMQLEDVDGALVGGASLNPEDFNEIIRF---
Planctomyces_brasiliensis       -EQMTNVVIAYEPVWAIGTGKTASPEQAEQAHA-HLRKWLADRYT--SEVAEQTRILYGGSVKPANAKELLGQQNVDGALVGGASLTVDNFGPIIDAGVE
Spirochaeta_coccoides           -ADMSRVVIAYEPVWAIGTGKTATPDDADNAHA-YIRGLINGLYG--KDVAEQLIIQYGGSVKADNVKELMAKPNVDGALVGGASLSIDKFLPIIEFGL-
Denitrovibrio_acetiphilus       -DRINRVTLAYEPVWAIGTGKTATPADAEDIHG-KIRTQLSKMYG--PVVAEKIRILYGGSVKPDNVSALMMRENIDGALVGGASLDATSFAKLVNFNG-
Prosthecochloris_aestuarii      -ADIGDIVIAYEPVWAIGTGKTASSLQAQEVHA-SIRATVAGLYS--EPAAAGVRIQYGGSVKPSNAEELFAMPDIDGGLIGGASLNAEDFVAIVKAAEK
Chlorobium_phaeobacteroides     -EDLGNVVLAYEPVWAIGTGKTATPEQADAVHA-SIRATISDMYG--EADAEDIRIQYGGSVKPSNAVELFGMPNIDGGLIGGASLKADDFVAIVNAAG-
Chlorobium_ferrooxidans         -SDISTVVIAYEPVWAIGTGKTASSAQAEEVHL-LIRNTITKLYG--DAAAGKLRIQYGGSVKPSNAAELFAMPNIDGGLIGGASLNAADFAAIIKAAS-
Chloroherpeton_thalassium       -EDMKSVVIAYEPVWAIGTGKTATPEQAQEVHA-FIRGIVKDMFG--EEVANELRIQYGGSVKTSNAKELFGMPDIDGGLIGGASLNAEDFVEIIKSAE-
Treponema_pallidum              -SLMHNVTVAYEPVWAIGTGKTATPAQANAVHA-HIRSVVREMYG--AAIAEALCIQYGGSMKAENARALLAEEHIDGGLIGGASLEAASFVPIARSV--
Leptospira_interrogans          -VFFSNLILAYEPVWAIGTGKVATPSQAQEVHS-FIRKEISGLFVGASSISESISILYGGSVKPDNIQDLLKEKDIDGGLVGGASQKISSFAELF-----
Borrelia_turicatae              -SDLKRIILAYEPVWAIGTGKTATKEEAQEVHR-AIRHEVELLYS--VSAANNIIIQYGGSVNIDNVKGLMGESDIDGALIGGASLKADSFLNIVNKVAK
Thermotoga_maritima             -EEAKRVVIAYEPVWAIGTGRVATPQQAQEVHA-FIRKLLSEMYD--EETAGSIRILYGGSIKPDNFLGLIVQKDIDGGLVGGASLK-ESFIELARIMRG
Fusobacterium_nucleatum         -EDAEKTIVAYEPVWAIGTGKTATPEMAQETHK-EIRNVLAEMFG--KDVADKMIIQYGGSMKPENAKDLLSQEDIDGGLVGGASLKADSFFEIIKAGN-
Selenomonas_sputigena           -DEAAQLVIAYEPIWAIGTGKTATFEQAEEVCA-AIRATVAEVFN--KDAAEGIRIQYGGSVKPATIAGLMEKPNVDGALVGGASLKAKDFSEIVKF---
Ruminococcus_sp                 -DQAKTAVIAYEPIWAIGTGKTATTEQAQEVCA-GIRACIAEIYD--EATAEAIRIQYGGSVNPATAPDLFVQNDIDGGLVGGASLKA-DFGKIVNYK--
```

```
Clostridium_botulinum              -EQVEKLVIAYEPIWAIGTGKTATDEQANETIG-YIRTVVKAMYG--ESVADKIRIQYGGSVKPGTIKAQMAKEEIDGALVGGASLKAEDFAAIVNY---
Clostridium_perfringens            -EQAEKVVIAYEPIWAIGTGKTATSDQANETIA-AIRAMVAEMFG--QEVADKVRIQYGGSVKPNTIAEQMAKSDIDGALVGGASLVAADFAQIVNY---
Thermoanaerobacter_wiegelii        -DDIVNVVIAYEPIWAIGTGKTATAKDANEVIK-AIRNTIASLYG--KGKASLVRIQYGGSVKPENISELMAESDIDGALVGGASLVASDFAKIVNY---
Brachyspira_murdochii              -AEAKKVVIAYEPVWAIGTGKTATPQDADEVHK-TIRETLKSLYN--ESVAEGMIILYGGSVNEKNADDLLNMPNIDGALVGGASLVADKFARIVNYIAK
Bartonella_henselae                -ATAENIIIAYEPVWAVGTGNTATSADVAEVHA-FIHHKMHSRF---GDEGAKIRLLYGGSVKPSNAFELLSTAHVNGALIGGASLKAIDFLTICDVYRK
Brucella_abortus                   -VTAENTIIAYEPVWAIGTGLTPTVQDVRAAHA-FMREQLIERF---GAKGAHLRLLYGGSVKPSNAAELLGVADVDGALVGGASLKAADFLAICETYRN
Rhizobium_etli                     -ATAENTVIAYEPIWAIGTGVTPTSGDVEKAHA-FMRAEMAARF---GEEGRKMRLLYGGSVKPANAGELMGIANVDGALIGGASLKAADFLAIYRAYEA
Agrobacterium_tumefaciens          -ATAENTVIAYEPVWAIGTGLTPTTQDVREAHA-FMRDELVKRF---GDAGKTMRILYGGSVKPANALELMGVENVDGALIGGASLKAADFLSIYAAYEQ
Rhodobacter_sphaeroides            -ATAANTVIAYEPVWAIGTGRTPTTAEIAEVHA-FLRARLAERF---SDA-EGFRLLYGGSVKPSNAAEIFAVPNVDGALVGGASLKAADFGGIVAALSA
Ruegeria_sp                        -VTAENTVVAYEPVWAIGTGKVPTLDQIAEVHD-ALRADLVARF---GAAGKDLPLLYGGSVKPGNAAEIFGVSNVDGALVGGASLKAADFGPIIAALAA
Bradyrhizobium_japonicum           -STAANLVVAYEPVWAIGTGLTPTVQDVEQIHG-FIREFLTSRF---SVDGAKMRILYGGSVKPSNAAELMAVKNVNGALVGGASLKAADFLAIAKGCP-
Rhodopseudomonas_palustris         -ATAANLVVAYEPVWAIGTGMTPTAADVEEVHG-FIRQTLTQRF---NAEGDQMRLLYGGSVKPSNARELMSVPHVNGALVGGASLKASDFLAIAAGCP-
Methylobacterium_radiotolerans     -ATAADTVIAYEPVWAIGSGRTPTPRDIAEVHA-SLREMLDKLV---GDEAQKIRILYGGSVKPGNAKELLSVDNVDGALVGGASLVAEDFLGICAAYA-
Rhodospirillum_rubrum              -ADSGKVVIAYEPVWAIGTGRVPTTEDVAKVHG-ALRKDLVGAL---GADGNGVRLLYGGSVKPDNAAELMALENVDGALVGGASLKAEDFWAIAQSCA-
Zymomonas_mobilis                  -ATSDNLVVAYEPIWAIGTGNTPPPESIAEMHA-SIRDRLCLLL---GSEGKKVRILYGGSVTGKNAEELMAIPDVNGTLVGGASLTAEQFVPIIEAGGR
Gluconacetobacter_hansenii         -FTG---IVAYEPVWAIGSGTAASQQDIADMTQ-FIREELVRQF---GDAGKTIKILYGGSVNGRNAADILPIADVGGALVGNASLAAETFMPIVRAAVD
Thermus_thermophilus               -PGPEALVIAYEPVWAIGTGKNATPEDAEAMHQ-EIRKALSERYG--EAFASRVRILYGGSVNPKNFADLLSMPNVDGGLVGGASLELESFLALLRIAG-
Thermus_aquaticus                  -ESPDRLVIAYEPVWAIGTGKNATPEDAEAMHQ-AIRQALAERYG--EAFASRVRVLYGGSVNPKNFADLLSMPNVDGGLVGGASLELESFLALLRMAG-
Oceanithermus_profundus            -ETGEELVIAYEPVWAIGTGKTASADDAQAMAA-AIRGFLAERYG--ESVAGRTRILYGGSMKPANTTEILAGPDVDGGLVGGASLEVASFSAMVEAAG-
Deinococcus_deserti                ----TDVVIAYEPVWAIGTGKTATAEDAEELAE-AIRGALTEQYG--EAAG-TLRILYGGSVKPDNIASICAKPNVNGALVGGASLKVADVVGMNDALK-
Bifidobacterium_bifidum            -KEAAKLIIAYEPVWAIGTGMVATPDTAQQAAK-AIRDDLGEMFG--PAVADTVRILYGGSVSSKNATHLIGEPDVDGFLIGGASLDPDELAKIARLTLK
Bifidobacterium_longum             -EQAAKLIVAYEPVWAIGTGMVATPQSAQDAAN-AIRNDLKTTFG--TKVSDSVRILYGGSVTSKNAAELISQPDVDGFLIGGAALDVEELAKIARLALK
Gardnerella_vaginalis              -KQAERLIIAYEPVWAIGTGMVATAQSAQDAAK-AIRDDLQETFG--DTVSQKVRILYGGSVTSSNAASLICEPDVDGFLIGGASLDIEELATIVQLSSY
Nocardia_farcinica                 -EQIAKVVIAYEPVWAIGTGKVASAADAQEVCG-AIRAELAELAG--PEVAAQVRVLYGGSVNAKNVGELVAQPDVDGALVGGASLKGDEFATLSAIAAG
Mycobacterium_tuberculosis         -EQIGSVVIAYEPVWAIGTGRVASAADAQEVCA-AIRKELASLAS--PRIADTVRVLYGGSVNAKNVGDIVAQDDVDGGLVGGASLDGEHFATLAAIAAG
Corynebacterium_glutamicum         -AELANTVIAYEPVWAIGTGKVASAADAQEVCK-AIRGLIVELAG--DEVAEGLRILYGGSVKAETVAEIVGQPDVDGGLVGGASLDGEAFAKLAANAAS
Propionibacterium_acnes            -EQVAKLVIAYEPIWAIGTGETATADDAQEVCQ-AIREAVKELYD--APTAEAVRIQYGGSVKPANVAEIMAKPDVDGALVGGASLKAGDFSKIVTFYEA
Actinomyces_odontolyticus          -EEVAKIVIAYEPIWAIGTGETASAEDAQEVCG-AIRAALAEDFG--AETAESTRILYGGSAKPDNIKELMAQPDVDGGLVGGASLKADSFAAMATFYA-
Microbacterium_testaceum           ---AAEIVVAYEPVWAIGSGQAATPEQAQEVCA-KLRAVVAEKLG--ADAAARTRVLYGGSVKANNIAAFMREPDVDGALVGGASLVVDEFAAIIRYQKH
Moritella_marina                   -EALEGAIIAYEPIWAIGTGKAATAEDAQRIHA-QIR-AHIAEKS--EAVAKNVVIQYGGSVKPENAAAYFAQPDIDGALVGGAALDAKSFAAIAKAAAE
Photobacterium_profundum           -EALEGAIIAYEPIWAIGTGKAATAEDAQRIHA-QIR-AHIAEKS--EEVAKNVVIQYGGSVKPENAAAYFAQPDIDGALVGGAALDAKSFAAIAKAAAE
Vibrio_cholerae                    -EALNGAIIAYEPIWAIGTGKAATADDAQRIHA-SIR-ALIAAKD--AAVAEQVIIQYGGSVKPENAASYFAQPDIDGALVGGASLDAKGFAAIAKAAAE
Shigella_flexneri                  -AAFEGAVIAYEPVWAIGTGKSATPAQAQAVHK-FIR-DHIAKVD--ANIAEQVIIQYGGSVNASNAAELFAQPDIDGALVGGASLKADAFAVIVKAAEA
Escherichia_coli                   -AAFEGAVIAYEPVWAIGTGKSATPAQAQAVHK-FIR-DHIAKVD--ANIAEQVIIQYGGSVNASNAAELFAQPDIDGALVGGASLKADAFAVIVKAAEA
Salmonella_enterica                -AAFEGAVIAYEPVWAIGTGKSATPAQAQAVHK-FIR-DHIAKAD--AKIAEQVIIQYGGSVNASNAAELFAQPDIDGALVGGASLKADAFAVIVKAAEA
Klebsiella_pneumoniae              -AAFEGVVIAYEPVWAIGTGKSATPAQAQAVHK-FIR-DHIAKAD--AKIAEQVIIQYGGSVNAGNAAELFTQPDIDGALVGGASLKADAFAVIVKAAEA
Enterobacter_aerogenes             -AAFEGVVIAYEPVWAIGTGKSATPAQAQAVHK-FIR-DHIAKAD--AKIAEQVIIQYGGSVNAGNAAELFTQPDIDGALVGGASLKADAFAVIVKAAEA
Serratia_symbiotica                -PAMKSAVIAYEPVWAIGTGKSATPAQAQAVHK-FIR-DHIAKHD--TAIAAEVIIQYGGSVNDKNAAELFAQPDIDGALVGGASLKADAFAAIVKAAGA
Yersinia_pestis                    -KAFEGAVIAYEPIWAIGTGKSATPAQAQAVHK-FIR-DHIAKQD--AAVAAQVIIQYGGSVNDKNAAELFTQPDIDGALVGGASLKADAFAVIVKAAAK
Actinobacillus_pleuropneumonia     -EAFNGAVIAYEPIWAIGTGKSATPAQAQAVHA-FIR-GHIAAKS--QAVADQVIIQYGGSVNDANAAELFTQPDIDGALVGGASLKAPAFAVIVKAAEK
Pseudomonas_aeruginosa             -GAFARAVVAYEPVWAIGTGLTASPAQAQEVHA-AIR-AQLAAEN--AEVAKGVRLLYGGSVKAASAAELFGMPDIDGGLVGGASLNADEFGAICRAAGS
Marinomonas_posidonica             -QAFENIVIAYEPVWAIGTGLSASAQQAQDVHQ-AIR-ANLASLS--TPVSEKVQILYGGSVKASTSAELFTMPDVDGALVGGASLDAKEFLDIVKAAG-
Xylella_fastidiosa                 -QGVARGLIAYEPVWAIGTGRHADPSQVQAMHA-FIR-GEIARQD--ARIGDSLLILYGGGIKPCNAAELFSQQDVDGGLIGGASLVADDFLAIARATV-
Xanthomonas_campestris             -AGFARAVVAYEPIWAIGTGRTATPDQAQAVHA-FIR-GEVAKAD--ARIADSLPILYGGSVKPDNASELFSQPDVDGGLVGGASLVAEDFLAIARAAAA
Marinobacter_algicola              -GLWQQVVIAYEPVWAIGTGKTATADDAQAMHA-AIR-AVLMSLG--A-PADEVSLLYGGSVKADNAAALFAQPDIDGGLIGGASLDASDFVSICRAVPG
Aeromonas_caviae                   -MAFDNAIIAYEPIWAVGTGKCATPEQAQEVHS-FIR-SRLAEDT--PVIGEKVRIIYGGSVTPANAQDLFAQSDIDGGLIGRASLDGEAFLGIVEAAKG
Pseudoalteromonas_haloplanktis     -AALKDSVIAYEPVWAIGTGKTASPEQAQSVHK-FIR-DKIASLN--SDLAQGLTILYGGSVNEKNSELLFAQTDIDGGLIGGASLKADSFTAICNSAKG
Legionella_pneumophila             -DCFRDCVVAYEPVWAIGTGKTATPEQAQKIHQ-FIR-DLVGEIN--DSDAKHLTLIYGGSVNENNAKALFSMPDIDGGLVGGASLNAKQFVEIVKCIN-
Ralstonia_solanacearum             -EQLGRIVVAYEPVWAIGTGKTATSEQAQAVHA-FLR-GRVAACD--AGVAQRMPILYGGSVKPDNAAELFSMADIDGGLIGGASLKAEDFLVIGRA---
Burkholderia_sp                    -QEAARLVVAYEPVWAIGTGKSASAAQAQAVHA-FLR-ARLAAKG-----AADVPLLYGGSVKPDNAEELFRQNDIDGGLIGGASLKDQDFLAICKAAAA
Bordetella_pertussis               -DALVRMVLAYEPVWAIGTGRTASPEQAQEVHS-AIR-VALDGLQ-----ASQVRVLYGGSVKGANAASLFAMPDIDGGLVGGASLVAEEFLRIAAA---
Nitrosococcus_watsonii             -NALKKAVIAYEPIWAIGTGRTATPEQAQEVHA-FIR-GHVAVQN--SGIAEELLVLYGGSVKGSNAAQLLAMPDIDGGLIGGASLNAKEFLTICQAAV-
Coxiella_burnetii                  -ASLEGMVVAYEPIWAIGTGKNATPSQAEEVHA-ALR-DQLHRQD--ATLAESTRLLYGGSVKPDNAAALFEMPNIDGALVGGASLEAEQFLKIGQQCNQ
Variovorax_paradoxus               -HCISEIVVAYEPVWAIGTGKTASPEQAQAVHA-VLR-AQLHHAA--SEHAAGISILYGGSMNAANAAELLAQADIDGGLIGGASLKAPDFLQIISATAR
Halomonas_elongata                 -GQRSRLVIAYEPVWAIGTGRTATPEQAQAIHA-AIR-RRLARYG--DGLAEGTQLLYGGSMKADNAADLLAQPDIDGGLVGGASLKIDDFLAICQSAG-
Sphaerobacter_thermophilus         -ADVLRTVIAYEPVWAIGTGRAATPEDAR-EMANWIREVLARHVG--AEAANEIRVQYGGSVNADNAAGFLSLPEIDGALVGGASLKADQFTQIVERAAA
Anaerolinea_thermophila            -EEGLKLVIAYEPVWAIGTGRAATAEVAQSVHANVVRPALAHLFG--QEVAGQIRIQYGGSVTAKNAPELFAMPDIDGALVGGASLKAGEFAAIVQAAAE
```

```
Myxococcus_xanthus                -KEVAGFVLAYEPVWAIGTGRNATAAQAQEVHA-AIRGLVERLYD--GETAGRVRIQYGGSVKPDNAAELLGQPDVDGALVGGASLKAGDFAAIVKAAT-
Roseiflexus_castenholzii          -EQIGRIVIAYEPVWAIGTGDTATPADAQAMHE-TIRRILGDMAG--SDTAATINILYGGSVKPDNIDDLMAQPDIDGALVGGASLKADSFLRIVHFLSP
Chloroflexus_aurantiacus          -DQMANVVIAYEPIWAIGTGDTATPADAQAMHA-AIRATLAELYG--SEIAATVRIQYGGSVKPDNIDELMAQPDIDGALVGGASLQAASFLRIIHYQ--
Collinsella_tanakaei              -ADPAQLVVAYEPIWAIGTGMTATADDAQEVCA-AVREALADLFG--AELASGIRVLYGGSAKPGNIAELTAKPDVDGALVGGASLKAADFSQMVVKSAE
Atopobium_vaginae                 -TSPSQYVVAYEPIWAIGTGKTATAEDAQEVCS-AIRATLRDIFG--AELANGIRVLYGGSAKPENIAGFLAEEDVDGALVGGASLKADSFAAMVKAAM-
Eggerthella_sp                    -TDAKVCVVAYEPIWAIGTGRTATPEQAEAVCA-AIRATLAELYG--ADVSEAMRVLYGGSMNPGNVEGLMAQPNIDGGLVGGASLDATSFAELVKACL-
Ostreococcus_lucimarinus          -DEMSKVIIAYEPVWAIGTGLTATPAIAQSVHA-GIRQWFRTNYG--ESVAAKIQIQYGGSVKPETVDELMQAPDVDGCLVGGASLNAEQF---------
Lentisphaera_araneosa             -EQMTNLVVAYEPVWAIGTGVTASPEQAQDTHA-YIRSEFAALYG--QEVADKVIIQYGGSVKPANVAELMSCEDIDGALVGGASLKADDFSALILNAAA
Acholeplasma_laidlawii            -EQALKTIVAYEPVWAIGTGKTATPQMANDTIK-DVRAKLSDLYG--AEVAAQVRILYGGSVKPDNIESLLQESDIDGALIGGAALDHKNFLTFTKAAKN
Lactobacillus_reuteri             -DQIKNVVVSYEPSWAVGYGQHANPVLAEEGCR-QIRRTIADNYT--YEIADKIRILYGGSVNPDNIGMIMNKPDVDGVLIGRASLDVDNFLRMVNYLKN
Bacillus_subtilis                 -EQVAASVIAYEPIWAIGTGKSSTAKDANDVCA-HIRKTVAESFS--QEAADKLRIQYGGSVKPANIKEYMAESDIDGALVGGASLEPQSFVQLLEEGQY
Bacillus_thuringiensis            -EQVKATVIAYEPIWAIGTGKSSSSADANEVCA-HIRKVVAEAVS--PEAAEAVRIQYGGSVKPENIKEYMAQSDIDGALVGGASLEPASFLGLLGAVK-
Listeria_monocytogenes            -EQVIKSVIAYEPIWAIGTGKSSTSADANETCA-VIRAEVADAVS--QKAADAVRIQYGGSVKPENIADYLAESDIDGALVGGASLEPASFLALLEAVK-
Geobacillus_stearothermophilus    -EQVKQAVIAYEPIWAIGTGKSSTPEDANSVCG-HIRSVVSRLFG--PEAAEAIRIQYGGSVKPDNIRDFLAQQQIDGPLVGGASLEPASFLQLVEAGRH
Staphylococcus_aureus             -DQLKSVVIAYEPIWAIGTGKSSTSEDANEMCA-FVRQTIADLSS--KEVSEATRIQYGGSVKPNNIKEYMAQTDIDGALVGGASLKVEDFVQLLEGAK-
Enterococcus_faecalis             -EQVASMVIAYEPIWAIGTGKSADANIADEICG-VVRSTVEKLYG--KEVSEAVRIQYGGSVKPENIAEYMAKENVDGALVGGASLEADSFLALLDAVK-
Lactococcus_lactis                -EQVSNLVIAYEPIWAIGTGKTATNEIADETCG-VVRSTVEKLYG--KEVSEAVRIQYGGSVKPETIEGLMAKENIDGALVGGASLEADSFLALLEMYK-
Streptococcus_pneumoniae          -EQVAASVIAYEPIWAIGTGKSASQDDAQKMCK-VVRDVVAADFG--QEVADKVRVQYGGSVKPENVASYMACPDVDGALVGGASLEAESFLALLDFVK-
Erysipelotrichaceae_bacterium     -KCVGNMVIAYEPIWAIGTGKSASVEIAENCCR-IVRDQVRVMYG--DEAAENVRVQYGGSVKPNNIVEYMAQPDIDGALIGGASLKADSFIEIIEKTK-
Tenebrio_Molitor                  ---WSNVVIAYEPVWAIGTGKTATPQQAQDVHK-ALRQWICENIDA--KVGNSIRIQYGGSVTAANCKELASQPDIDGFLVGGASLK-PEFVDIINARQL
Bombyx_mori                       ---WENIVLAYEPVWAIGTGKTATPQQAQDVHH-ALRNWLSANVSG--SVSDAVRIQYGGSVTAANAKELASCKDIDGFLVGGASLK-PEFVEIVNANQ-
Apis_mellifera                    ---WDNVVVAYEPVWAIGTGKTATPQQAQEVHE-KLRNWFSKNVNQ--TVAETVRIIYGGSVTAGNAKDLAKEKDIDGFLVGGASLK-PDFVQIVNAKQ-
Aedes_aegypti                     ---WSNVVIAYEPVWAIGTGKTATPEQAQEVHA-ALRKWFTENVSA--DVSGAIRIQYGGSVTAANCRELAAKPDIDGFLVGGASLK-PEFIQIVNARQ-
Anopheles_gambiae                 ---WSNVVIAYEPVWAIGTGKTATPEQAQEVHA-ALRKWFTENVSA--DVSNAIRIQYGGSVTAANCRELAAKPDIDGFLVGGASLK-PEFIQIVNARQ-
Drosophila_melanogaster           ---WKNVVVAYEPVWAIGTGQTATPDQAQEVHA-FLRQWLSDNISK--EVSASLRIQYGGSVTAANAKELAKKPDIDGFLVGGASLK-PEFVDIINARQ-
Rhipicephalus_microplus           ---WSKVVIAYEPVWAIGTGKTATPDQAQEVHS-KVRNWLSTNVSA--DVASKVRIQYGGSVNAGNCKELGRKPDIDGFLVGGASLK-PEFVQIINAMQG
Ixodes_scapularis                 ---WNRVVLAYEPVWAIGTGKTASPEQA-EVHA-QLRQWLSKNVSP--DVAKKVRIQYGGSVTAANCQELAKKPDVDGFLVGGASLK-PEFVEIINARQ-
Anolis_carolinensis               ---WSKVVLAYEPVWAIGTGKTATPQQAQEVHL-KLRGWLKSHVSD--AVSQSTRIIYGGSVTGATAKELASQPDLDGFLVGGASLK-PEFVDIINAKH-
Gallus_gallus                     ---WSKVVLAYEPVWAIGTGKTATPQQAQEVHE-KLRGWLKSHVSD--AVAQSTRIIYGGSVTGGNCKELASQHDVDGFLVGGASLK-PEFVDIINAKH-
Canis_lupusfamiliaris             ---WSKVVLAYEPVWAIGTGKTATPQQAQEVHE-KLRGWLKSNVSD--AVAQSTRIIYGGSVTGATCKELASQPDVDGFLVGGASLK-PEFVDIINAKQ-
Oryctolagus_cuniculus             ---WSKVVLAYEPVWAIGTGKTATPQQAQEVHE-KLRGWLKSNVSD--AVAQSTRIIYGGSVTGATCKELASQPDVDGFLVGGASLK-PEFVDIINAKQ-
Pan_troglodytes                   ---WSKVVLAYEPVWAIGTGKTATPQQAQEVHE-KLRGWLKSNVSD--AVAQSTRIIYGGSVTGATCKELASQPDVDGFLVGGASLK-PEFVDIINAKQ-
Homo_sapiens                      ---WSKVVLAYEPVWAIGTGKTATPQQAQEVHE-KLRGWLKSNVSD--AVAQSTRIIYGGSVTGATCKELASQPDVDGFLVGGASLK-PEFVDIINAKQ-
Nomascus_leucogenys               ---WSKVVLAYEPVWAIGTGKTATPQQAQEVHE-KLRGWLKSNVSD--AVAQSTRIIYGGSVTGATCKELASQPDVDGFLVGGASLK-PEFVDIINAKQ-
Pongo_abelii                      ---WSKVVLAYEPVWAIGTGKTATPQQAQEVHE-KLRGWLKSNVSD--AVAQSTRIIYGGSVTGATCKELASQPDVDGFLVGGASLK-PEFVDIINAKQ-
Macaca_mulatta                    ---WSKVVLAYEPVWAIGTGKTATPQQAQEVHE-KLRGWLKSNVSE--AVAQSTRIIYGGSVTGATCKELASQPDVDGFLVGGASLK-PEFVDIINAKQ-
Bos_taurus                        ---WSKVVLAYEPVWAIGTGKTATPQQAQEVHE-KLRGWLKSNVSD--AVAQSARIIYGGSVTGATCKELASQPDVDGFLVGGASLK-PEFVDIINAKQ-
Sus_scrofa                        ---WNKVVLAYEPVWAIGTGKTATPQQAQEVHE-KLRGWLKTHVPE--AVAHSTRIIYGGSVTGATCKELASQPDVDGFRVSGASLK-PEFVDIINAK--
Mus_musculus                      ---WSKVVLAYEPVWAIGTGKTATPQQAQEVHE-KLRGWLKSNVND--GVAQSTRIIYGGSVTGATCKELATPADVDGFLVGGASLK-PEFVDIINAKQ-
Rattus_norvegicus                 ---WCKVVLAYEPVWAIGTGKTATPQQAQEVHE-KLRGWLKCNVSE--GVAQCTRIIYGGSVTGATCKELASQPDVDGFLVGGASLK-PEFVDIINAKQ-
Xenopus_laevis                    ---WSKVVLAYEPVWAIGTGKTATPEQAQEVHK-KLREWVKTNVSE--GVAQSVRIIYGGSVTGGTCRELAGQPDIDGFLVGGASLK-PEFIEIINAKH-
Danio_rerio                       ---WSKVVLAYEPVWAIGTGKTASPQQAQEVHD-KLRQWLKTNVSE--AVANSVRIIYGGSVTGGTCKELASQKDLDGFLVGGASLK-PEFIDIINAKA-
Ictalurus_punctatus               ---WSKVVVAYEPVWAIGTGKTASPQQAQEVHD-KLRQWMSANISE--AVANSVRIIYGGSVTGATCKELASQKDVDGFLVGGASLK-PEFIDIINAKV-
Oreochromis_niloticus             ---WSKVVLAYEPVWAIGTGKTASPQQAQEVHD-KLREWLKTNVSE--AVANSVRIIYGGSVTGGTCKELASQKDVDGFLVGGASLK-PEFIDIINAKA-
Nematostella_vectensis            --DWSKVVIAYEPVWAIGTGKTATPQQAQEVHN-QLRAWLKENVSV--DVAESTRIIYGGSVNAKNCRELAAEGDIDGFLVGGASLK-PEFVQIVNARL-
Schistosoma_mansoni               -DEWKRVVVAYEPVWAIGTGKVATPQQAQEVHN-FLRKWFKTNAPN--GVDEKIRIIYGGSVTAANCKELAQQHDVDGFLVGGASLK-PEFTEICKARQR
Strongylocentrotus_purpuratus     ---WGNVVIAYEPVWAIGTGKTATPQQAQEVHQ-NLRSWLTEHVNA--AVAANTRIVYGGSVNGGNCCDIGQQPDVDGFLVGGASLK-PEFKQIIAAGSA
Caenorhabditis_elegans            ---WENIVIAYEPVWAIGTGKTASGEQAQEVHE-WIRAFLKEKVSP--AVADATRIIYGGSVTADNAAELGKKPDIDGFLVGGASLK-PDFVKIINARS-
Brugia_malayi                     ---WNKIVIAYEPVWAIGTGKTASPEQAQEVHG-WIREFLKEKVSA--DVAQKTRILYGGSVTAENAAELAKKPDIDGFLVGGASLK-PDFIKIIHARD-
Ricinus_communis                  ---WADVVLAYEPVWAIGTGKVATPAQAQEVHF-ELRKWLKENTSS--QVAATTRIIYGGSVTATNCKELAAQPDVDGFLVGGASLK-PEFIEIIKSAEV
Vitis_vinifera                    ---WANVVLAYEPVWAIGTGKVATPAQAQEVHS-ELRNWFQANASP--EVAATIRIIYGGSVSGANCKELAAKPDVDGFLVGGASLK-PEFIDIIKSAEV
Arabidopsis_thaliana              ---WSNVVIAYEPVWAIGTGKVASPAQAQEVHD-ELRKWLAKNVSA--DVAATTRIIYGGSVNGGNCKELGGQADVDGFLVGGASLK-PEFIDIIKAAEV
Zea_mays                          ---WSNVVVAYEPVWAIGTGKVATPAQAQEVHA-SLRDWLKTNASP--EVAESTRIIYGGSVTAANCKELAAQPDVDGFLVGGASLK-PEFIDIINAATV
Oryza_sativa                      ---WTNVVVAYEPVWAIGTGKVATPDQAQEVHD-GLRKWLAANVSA--EVAESTRIIYGGSVTGANCKELAAKPDVDGFLVGGASLK-PEFIDIINSATV
Glycine_max                       ---WDNVVLAYEPVWAIGTGKVATPAQAQEVHA-DLRKWVHDNVSA--EVAASVRIIYGGSVNGGNCKELAAQPDVDGFLVGGASLK-AEFVDIINAATV
Perkinsus_marinus                 ---WNNIVIAYEPVWAIGTGKVATTEQAQETHA-SIRAYLKSKVST--EVAENTRILYGGSVNPGNCGELIKCADVDGFLVGGASLK-PQFTEIIAAAAQ
Chlamydomonas_reinhardtii         ---WTKVVLAYEPVWAIGTGVVASPEQAQEVHA-YLRQYCAKKLGA--AVADKLRIIYGGSVSDTNCKDLSKQEDIDGFLVGGASLKGAAFVTICNAAGP
```

```
Trypanosoma_brucei                 -ADWAKVVIAYEPVWAIGTGKVATPQQAQEAHA-LIRSWVSSKIGA--DVAGELRILYGGSVNGKNARTLYQQRDVNGFLVGGASLK-PEFVDIIKATQ-
Trypanosoma_cruzi                  -EAWAHVVIAYEPVWAIGTGKVATPQQAQEVHE-LLRRWVRSKLGA--DIAAQLRILYGGSVTAKNARTLYQMRDINGFLVGGASLK-PEFVEIIEATK-
Leishmania_mexicana                -DAWNQVVLAYEPVWAIGTGKVATPEQAQEVHL-LLRKWVSENIGT--DVAAKLRILYGGSVNAANAATLYAKPDINGFLVGGASLK-PEFRDIIDATR-
Entamoeba_histolytica              -EAWKNIILAYEPVWAIGTGKTATPDQAQEVHQ-YIRKWMTENISK--EVAEATRIQYGGSVNPANCNELAKKADIDGFLVGGASLDAAKFKTIINSVSE
Plasmodium_falciparum              ---FDNVILAYEPLWAIGTGKTATPEQAQLVHK-EIRKIVKDTCGE--KQANQIRILYGGSVNTENCSSLIQQEDIDGFLVGNASLK-ESFVDIIKSAM-
Toxoplasma_gondii                  ---WDRVVIAYEPVWAIGTGKVATPAQAQEVHE-HIREFLKAKVSE--DVANKVRIVYGGSVNASNSTELILQPDLDGFLVGGASLK-KDFLDIIASGMK
Cryptosporidium_parvum             ---LSNLVIAYEPIWAIGTGVVATPGQAQEAHA-FIREYVTRMYNP--QVSSNLRIIYGGSVTPDNCNELIKCADIDGFLVGGASLK-PTFAKIIESAQ-
Paramecium_tetraurelia             --LWSKVVVAYEPVWAIGTGRTATPEQAQEVHA-FIRGWLKSQIGT--QAEQATRIIYGGSVTEKNAADLIKQPDLDGFLVGGAALK-PGFADIVAAND
Aspergillus_niger                  --QWAKVVIAYEPVWAIGTGKVATTAQAQEVHA-AIRKWLVDAISA--EAADNTRIIYGGSVSEKNCRDLAKEADVDGFLVGGASLK-PAFVDIINARL-
Paracoccidioides_brasiliensis      --EWNKVVIAYEPIWAIGTGKVATTEQAQEVHA-SIRKWLNEKISP--EAAENIRVIYGGSVTENNCRDLAAQPDVDGFLVGGASLK-PAFVDIINARL-
Schizosaccharomyces_pombe          --NWSKIVIAYEPVWAIGTGKTATPEQAQEVHA-EIRKWATNKLGA--SVAEGLRVIYGGSVNGGNCKEFLKFHDIDGFLVGGASLK-PEFHNIVNVHSL
Saccharomyces_cerevisiae           --DWTNVVVAYEPVWAIGTGLAATPEDAQDIHA-SIRKFLASKLGD--KAASELRILYGGSANGSNAVTFKDKADVDGFLVGGASLK-PEFVDIINSRN-
Kluyveromyces_lactis               --DWTNVVVAYEPVWAIGTGLAATAEDAQDIHH-SIREFLAEKLSR--DVADSVRILYGGSANGKNAVTFKDKADVDGFLVGGASLK-PEFVDIINSRV-
Candida_dubliniensis               --DWTNIVVAYEPVWAIGTGLAATPDDAEETHK-GIRAHLAKTIGA--EQAEKTRILYGGSVNGKNAKDFKDKANVDGFLVGGASLK-PEFVDIIKSRL-
Giardia_lamblia                    KMLWKEVVIAYEPVWSIGTGVVATPEQAEEVHV-GLRKWFAEKVCA--EGAQHIRIIYGGSANGSNCEKLGQCPNIDGFLVGGASLK-PEFMTMIDILTK
Trichomonas_vaginalis              --KWDDVVIAYEPIWAIGTGKVASTQDAQEMCK-VIRDILAAKVGA--DIANKVRILYGGSVKPNNCNELAACPDVDGFLVGGASLE-PGFINIVNSNVH
Sphingobacterium_sp                --DFSKIILAYEPVWAIGTGLTASPEQAQEVHA-FIRKQIEAKYGA--AVADETSVLYGGSCNPKNAPDLFSQPDIDGGLIGGASLKSRDFTDIVKVFN-
Pedobacter_saltans                 --DFSKLVIAYEPVWAIGTGVTASSAQAQEIHA-FIRSEVAAKYGA--EVAENTTILYGGSCNPGNAAELFAQKDIDGGLIGGASLKSRDFVDIVKTFNN
Marivirga_tractuosa                --QFKNIVIAYEPIWAIGTGKTASSQQAQDMHK-TIREHIAAKFGQ--DAANKTSILYGGSCKPDNAKELFSQPDVDGGLIGGASLKSRDFTDIIKSF--
Cyclobacterium_marinum             --EIKKLVIAYEPIWAIGTGKTASSEQAQDMHA-AIRKHLSSKYGE--AVAEEISILYGGSCKPSNAKEIFSKADVDGGLIGGASLKSRDFVDIAESF--
Leadbetterella_byssophila          --AIQQVVIAYEPIWAIGTGVTASSDQAQEMHK-VLRDHLASKYGA--AVAEEISILYGGSVNAGNAEELFSQPDVDGGLVGGASLKSRDFTDISKSFPA
Haliscomenobacter_hydrossis        --EFQKVVIAYEPIWAIGTGRTASPEQAQDMHA-AIRALLTDQYGA--EIADATTILYGGSVNGGNAAVLFSQPDVDGGLVGGASLKAEEFITIVEATKK
Parabacteroides_distasonis         --DFGKIILAYEPVWAIGTGKTASAEQAEEIHA-HIRATLAAKYGN--EVADNCTILYGGSCNAGNAKELFAKPNVDGGLIGGASLAVDKFMPIIEAF--
Bacteroides_fragilis               --DFSKIVLAYEPVWAIGTGKTASPAQAQEIHA-FIRSAVAEKYGK--EIADNTSILYGGSCKPSNAKELFANPDVDGGLIGGAALKVADFKGIIDAFN-
Prevotella_ruminicola              --DFRKIVIAYEPIWAIGTGKTATAEQAEEIHA-YIRSIIAEKYGQ--AVADDTTILYGGSCKASNAPELFAKPDIDGGLIGGASLKAADFKGIIDAWKK
Alistipes_shahii                   --QYKNLVIAYEPVWAIGTGKTASAEQAQEIHA-YIRQVLTAKFG---AAAQETAILYGGSCKPSNAAEIFAKEDVDGGLIGGAALKAEDFLAIGKGFSK
Porphyromonas_asaccharolytica      --QMEQIVIAYEPVWAIGTGLTATPDQAQEMHQ-HIRQTVASLFDQ--KLADLTTILYGGSCKASNAESLFSQPDVDGGLIGGAALKADTFLPIITANK-
Cellulophaga_lytica                --AWSNIVLAYEPVWAIGTGETASPEQAQEMHA-FIRKTIADEYTT--EIAEAVSILYGGSVKPANAREIFSKADVDGGLIGGAALKADDFIAIIDAI--
Kordia_algicida                    --AWKNIVLAYEPVWAIGTGETASPEQAQEMHA-FIRKTVSEKFGD--ETGNGVSILYGGSVKPANAAEIFAKEDVDGGLIGGASLKAADFVAIINAI--
Flavobacterium_psychrophilum       --AWANIILAYEPVWAIGTGETASPEQAQEMHE-FIRETVRKVFGS--DIAEDVSILYGGSVKPDNAKEIFSKPDVDGGLIGGAALKADDFVVIVNGI--
Neisseria_gonorrhoeae              --NIA---VAYEPVWAIGTGKVATVEQIADMHA-FIYKEILSLCGS---DVK-IRVLYGGSVKADNAADIFAVPYVDGALVGGASLSYDSFTAIISAAQN
Francisella_tularensis             --QLAKVVIAYEPVWAIGTGVVASLEQIQETHQ-FIRSLLAKVDER---LAKNIKIVYGGSLKAENAKDILSLPDVDGGLIGGASLKAAEFNEIINQANK
Mycoplasma_genitalium              --LIKNLVIAYEPLWAIGTGKTATPEVANQTIK-TIREYINDLYD--ENVANNISILYGGSVDHNNIQKLAIMEQIDGFLVGKASLEIKNFLEMARVYA-
Ureaplasma_urealyticum             ---LKKIIIAYEPIWAIGTNLTLDLKHINHMIE-GIKTYLYNCTGL--N----IPILYGGSVNANNIKELCTQKLIDGFLIGNASLDVNNFNQIINACK-
Ureaplasma_parvum                  ---LKKIIIAYEPIWAIGTDLELDFKHINYMIE-GIKTYLYNCTGI--N----IPILYGGSVNDNNINELCNQKLIDGFLIGNASLDVNVFNKIIDKCK-
Buchnera_aphidicola                TSAFKNIIIAYEPIWAIGTGVSADPEHVQLIHV-FIKNYILKYSSI--NR-NDIIIQYGGSINHTNVKKFIEQPDINGLLIGNSSLSAKEFLEIIKIAHE
Campylobacter_jejuni               K-----LIIAYEPIYSIGTGVSAQSTDIAKVLE-FLASLT------------KAPLLYGGSVNENNIKEILSVNHCGGVLIGSAALKVENFIKLIKG---
Helicobacter_pylori                N-----LIVAYEPIWAIGTKKSASLEDIYLTHG-FLKQILN----------QKTPLLYGGSVNIQNAKEILGIDSVDGLLIGSASWELENFKTIISFL--
Orientia_tsutsugamushi             KN----VIIAYEPIWSIGSDMIPTIDDIYEVVT-MIREIQNRYIPH--NIENSVKIVYGGSVSANNIHQILTAG-VDGVLIGKASLKLESLTTIIKTVQG
Wolbachia_sp                       GE----YTVAYEPIWAIGTGHVPNNDAIAKVIE-VIKLCTS-----------KKHIIYGGSVSSENIENLLNISNLSGVLIGSASLDFDHFYKIIQQVEK
Methanocaldococcus_Jannaschii      --------IAVEPPELIGTGIPVSKANPEVVEG------TVRAVK---EINKDVKVLCGAGISKGEDVKAALDLGAEGVLLASGVVKAKNVEEAIRELIK
Methanococcus_voltae               --------IAVEPPELIGSGIPVSKANPEVVEG------TVSAVH---DINKDVKVLCGAGISKGEDVKSALELGAEGVLLASGVVKAKDVEQSIRDLIS
Methanothermococcus_okinawensi     --------IAIEPPELIGTGIPVSKANPEVVEG------TVKEVR---GINKDVKILCGAGISKGEDVSSALELGACGVLLASGVVKSKDVEGSIQELIK
Pyrococcus_Woesei                  --------VAVEPPELIGTGIPVSKAKPEVITN------TVELVK---KVNPEVKVLCGAGISTGEDVKKAIELGTVGVLLASGVTKAKDPEKAIWDLVS
Pyrococcus_furiosus                --------VAVEPPELIGTGIPVSKAKPEVITN------TVELVK---KVNPEVKVLCGAGISTGEDVKKAIELGTVGVLLASGVTKAKDPEKAIWDLVS
Thermofilum_pendens                --------VAIEPPELIGTGIPVSKAKPEVVTS------SVELVK---KVNPNVKVLCGAGITVGEDVAAALRLGTVGVLLASGVVKAKDWEKAILDLIS
Methanothermobacter_thermautot     --------VAVEPPELIGSGIPVSRAEPEVITG------SVDAVK---KVNPEVSVLCGAGISTGDDMKAAVDLGAEGVLLASGIILADSPRDALLDLVS
Methanosaeta_thermophila           --------VAVEPPELIGSGIPVSKADPEVVRG------SVEAVR---AIEKDVGVLCGAGITHGDDLRAAIELGAVGVLLASGIVKAKDQRKALEDLVT
Natronobacterium_gregoryi          --------VAVEPPELIGTGTPVSQADPGVVEN------AVVAAE---NVDDDVSVLCGAGISTGEDVTAADDLGTEGVLLASGVAKADNPRAALDDLVE
Halobacterium_salinarum            --------VAVEPPALIGTGTPVSQADPDIVSD------AVAAAE---AVDPSVDVYCGAGITTGEDVVSAGDLGASGVLLASGVAKADDPRAALADLVA
Halogeometricum_borinquense        --------VAVEPPELIGGDDSVATADPDIVRD------AVEAAA---NVDESVEVFCGAGISSGDDVDAAGDLGATGILLASGVAKADDPRAALEDLVE
Methanosphaerula_palustris         --------VAIEPPELIGSGVSVAKADPGIIQR------SVAAVH---AVNPKVKVLTGAGIQSGECVKIARDLGTDGVLLASSVVKVEDPAIVLRDLVS
Thermoproteus_tenax                --------VAVEPPELIGTGRAVSRYKPEAIVE------TVGLVS---RHFPEVSVITGAGIESGDDVAAALRLGTRGVLLASAAVKAKDPYAKIVELAK
Pyrobaculum_islandicum             --------VAVEPPELIGTGKAVSKYKPETIIE------TVRLVT---KHFPNVVVITGAGIETGEDVEAALKLGTKGVLLASAAVKAKDHYQKIFELAK
Cenarchaeum_symbiosum              --------IAIEPPELIGSGRSVSSERPELIGE------AAEAIR---GADG-TKLLCGAGITSGADVRKALELGSKGILVASGVVKSSDPAAAIAELAQ
Nitrosopumilus_maritimus           --------IAIEPPELIGSGKAVSTERPELITK------AANAVK---SANNNTKLLCGAGIVSGQDVSKAVELGSKGILVASGIIKAKNWDKIISEFAK
Desulfurococcus_kamchatkensis      --------VAVEPPELIGTGVSVSKAKPEVVTN------SVDMIR---RVNKDVAILTGAGITTGEDAYIAVKLGTIGVLVASGIVKAKDPYQVMKEMAE
```

```
Pyrolobus_fumarii                 --------VAVEPPELIGTGIAVSRAKPEVVTK------AVESVT---RVSPNVPVLVGAGIVSREDARRSVELGARGVLVASAVMKAADPYAKMRELAE
Acidilobus_saccharovorans         --------IAIEPPELIGTGVSVSKARPEVITE------GVKAVK---AVA-DIPVLAGAGITYREDVVRAVQLGASGILLASAVMKAADPDKALSEFVD
Sulfolobus_islandicus             --------ILIEPPELIGTGISVSKAKPEVITK------AVDEIR---KSE-GIYLIAGAGITTGEDVYKALKLGAHGIGVASAVMKAKEPEKVVEDFIT
Clustal Consensus                         .  **   :*                                     :  *..                *  :. .

                                          310
                                  ....|....|.
Dictyoglomus_thermophilum         -----------
Aquifex_aeolicus                  -----------
Thermodesulfovibrio_yellowston    -----------
Dehalogenimonas_lykanthroporep    VQS--------
Dehalococcoides_sp                IQNKH------
Nostoc_punctiforme                -----------
Anabaena_variabilis               -----------
Trichodesmium_erythraeum          -----------
Crocosphaera_watsonii             -----------
Synechocystis_sp                  -----------
Synechococcus_sp                  -----------
Cyanobacterium_UCYN               -----------
Prochlorococcus_marinus           -----------
Chlamydophila_pneumoniae          -----------
Desulfovibrio_vulgaris            -----------
Mesorhizobium_opportunistum       AI---------
Geobacter_metallireducens         -----------
Desulfobacter_postgatei           -----------
Planctomyces_brasiliensis         LSA--------
Spirochaeta_coccoides             -----------
Denitrovibrio_acetiphilus         -----------
Prosthecochloris_aestuarii        S----------
Chlorobium_phaeobacteroides       -----------
Chlorobium_ferrooxidans           -----------
Chloroherpeton_thalassium         -----------
Treponema_pallidum                -----------
Leptospira_interrogans            -----------
Borrelia_turicatae                -----------
Thermotoga_maritima               VIS--------
Fusobacterium_nucleatum           -----------
Selenomonas_sputigena             -----------
Ruminococcus_sp                   -----------
Clostridium_botulinum             -----------
Clostridium_perfringens           -----------
Thermoanaerobacter_wiegelii       -----------
Brachyspira_murdochii             -----------
Bartonella_henselae               L----------
Brucella_abortus                  L----------
Rhizobium_etli                    LLA--------
Agrobacterium_tumefaciens         LTA--------
Rhodobacter_sphaeroides           A----------
Ruegeria_sp                       S----------
Bradyrhizobium_japonicum          -----------
Rhodopseudomonas_palustris        -----------
Methylobacterium_radiotolerans    -----------
Rhodospirillum_rubrum             -----------
Zymomonas_mobilis                 LA---------
Gluconacetobacter_hansenii        I----------
Thermus_thermophilus              -----------
Thermus_aquaticus                 -----------
```

```
Oceanithermus_profundus             -----------
Deinococcus_deserti                 -----------
Bifidobacterium_bifidum             ATRR-------
Bifidobacterium_longum              STKSRN-----
Gardnerella_vaginalis               KNRI-------
Nocardia_farcinica                  GPLP-------
Mycobacterium_tuberculosis          GPLP-------
Corynebacterium_glutamicum          VA---------
Propionibacterium_acnes             -----------
Actinomyces_odontolyticus           -----------
Microbacterium_testaceum            VGV--------
Moritella_marina                    AKA--------
Photobacterium_profundum            AKA--------
Vibrio_cholerae                     AKKA-------
Shigella_flexneri                   AKQA-------
Escherichia_coli                    AKQA-------
Salmonella_enterica                 AKQA-------
Klebsiella_pneumoniae               AKKA-------
Enterobacter_aerogenes              AKKA-------
Serratia_symbiotica                 AKKA-------
Yersinia_pestis                     AKKA-------
Actinobacillus_pleuropneumonia      AKA--------
Pseudomonas_aeruginosa              -----------
Marinomonas_posidonica              -----------
Xylella_fastidiosa                  -----------
Xanthomonas_campestris              C----------
Marinobacter_algicola               KS---------
Aeromonas_caviae                    AS---------
Pseudoalteromonas_haloplanktis      TV---------
Legionella_pneumophila              -----------
Ralstonia_solanacearum              -----------
Burkholderia_sp                     TTVAD------
Bordetella_pertussis                -----------
Nitrosococcus_watsonii              -----------
Coxiella_burnetii                   SF---------
Variovorax_paradoxus                -----------
Halomonas_elongata                  -----------
Sphaerobacter_thermophilus          LRL--------
Anaerolinea_thermophila             AKA--------
Myxococcus_xanthus                  -----------
Roseiflexus_castenholzii            QE---------
Chloroflexus_aurantiacus            -----------
Collinsella_tanakaei                -----------
Atopobium_vaginae                   -----------
Eggerthella_sp                      -----------
Ostreococcus_lucimarinus            -----------
Lentisphaera_araneosa               LS---------
Acholeplasma_laidlawii              K----------
Lactobacillus_reuteri               DQEK-------
Bacillus_subtilis                   E----------
Bacillus_thuringiensis              -----------
Listeria_monocytogenes              -----------
Geobacillus_stearothermophilus      E----------
Staphylococcus_aureus               -----------
Enterococcus_faecalis               -----------
Lactococcus_lactis                  -----------
Streptococcus_pneumoniae            -----------
```

```
Erysipelotrichaceae_bacterium   -----------
Tenebrio_Molitor                -----------
Bombyx_mori                     -----------
Apis_mellifera                  -----------
Aedes_aegypti                   -----------
Anopheles_gambiae               -----------
Drosophila_melanogaster         -----------
Rhipicephalus_microplus         -----------
Ixodes_scapularis               -----------
Anolis_carolinensis             -----------
Gallus_gallus                   -----------
Canis_lupusfamiliaris           -----------
Oryctolagus_cuniculus           -----------
Pan_troglodytes                 -----------
Homo_sapiens                    -----------
Nomascus_leucogenys             -----------
Pongo_abelii                    -----------
Macaca_mulatta                  -----------
Bos_taurus                      -----------
Sus_scrofa                      -----------
Mus_musculus                    -----------
Rattus_norvegicus               -----------
Xenopus_laevis                  -----------
Danio_rerio                     -----------
Ictalurus_punctatus             -----------
Oreochromis_niloticus           -----------
Nematostella_vectensis          -----------
Schistosoma_mansoni             -----------
Strongylocentrotus_purpuratus   -----------
Caenorhabditis_elegans          -----------
Brugia_malayi                   -----------
Ricinus_communis                KKSV-------
Vitis_vinifera                  KKNC-------
Arabidopsis_thaliana            KKSA-------
Zea_mays                        KSA--------
Oryza_sativa                    KSA--------
Glycine_max                     KKN--------
Perkinsus_marinus               SA---------
Chlamydomonas_reinhardtii       KAKP-------
Trypanosoma_brucei              -----------
Trypanosoma_cruzi               -----------
Leishmania_mexicana             -----------
Entamoeba_histolytica           KF---------
Plasmodium_falciparum           -----------
Toxoplasma_gondii               KNE--------
Cryptosporidium_parvum          -----------
Paramecium_tetraurelia          AR---------
Aspergillus_niger               -----------
Paracoccidioides_brasiliensis   -----------
Schizosaccharomyces_pombe       -----------
Saccharomyces_cerevisiae        -----------
Kluyveromyces_lactis            -----------
Candida_dubliniensis            -----------
Giardia_lamblia                 TRT--------
Trichomonas_vaginalis           SK---------
Sphingobacterium_sp             -----------
Pedobacter_saltans              -----------
```

```
Marivirga_tractuosa                -----------
Cyclobacterium_marinum             -----------
Leadbetterella_byssophila          -----------
Haliscomenobacter_hydrossis        -----------
Parabacteroides_distasonis         -----------
Bacteroides_fragilis               -----------
Prevotella_ruminicola              -----------
Alistipes_shahii                   -----------
Porphyromonas_asaccharolytica      -----------
Cellulophaga_lytica                -----------
Kordia_algicida                    -----------
Flavobacterium_psychrophilum       -----------
Neisseria_gonorrhoeae              A----------
Francisella_tularensis             ICTE-------
Mycoplasma_genitalium              -----------
Ureaplasma_urealyticum             -----------
Ureaplasma_parvum                  -----------
Buchnera_aphidicola                HYS--------
Campylobacter_jejuni               -----------
Helicobacter_pylori                -----------
Orientia_tsutsugamushi             LD---------
Wolbachia_sp                       KFSLINSKISN
Methanocaldococcus_Jannaschii      FI---------
Methanococcus_voltae               EL---------
Methanothermococcus_okinawensi     HI---------
Pyrococcus_Woesei                  GI---------
Pyrococcus_furiosus                GIIKE------
Thermofilum_pendens                PIIKK------
Methanothermobacter_thermautot     KV---------
Methanosaeta_thermophila           GV---------
Natronobacterium_gregoryi          PL---------
Halobacterium_salinarum            PL---------
Halogeometricum_borinquense        PL---------
Methanosphaerula_palustris         LL---------
Thermoproteus_tenax                PLSELR-----
Pyrobaculum_islandicum             PLTVAAEPP--
Cenarchaeum_symbiosum              AMS--------
Nitrosopumilus_maritimus           ALV--------
Desulfurococcus_kamchatkensis      AMLKGLA----
Pyrolobus_fumarii                  GLAPRR-----
Acidilobus_saccharovorans          ALSSVA-----
Sulfolobus_islandicus              SALKAISS---
Clustal Consensus
```
